# New Lipophenol Antioxidants Reduce Oxidative Damage in Retina Pigment Epithelial Cells

**DOI:** 10.3390/antiox7120197

**Published:** 2018-12-19

**Authors:** Espérance Moine, Philippe Brabet, Laurent Guillou, Thierry Durand, Joseph Vercauteren, Céline Crauste

**Affiliations:** 1 Institute of Biomolecules Max Mousseron (IBMM), UMR 5247-CNRS-UM-ENSCM, Faculty of Pharmacy, 15 av. Charles Flahault, 34093 Montpellier, France; esperance.moine@umontpellier.fr (E.M.); thierry.durand@umontpellier.fr (T.D.); joseph.vercauteren@umontpellier.fr (J.V.); 2 Institute for Neurosciences of Montpellier, INSERM U1051-UM, Hospital St Eloi, 80 rue Augustin Fliche, 34091 Montpellier, France; philippe.brabet@inserm.fr (P.B.); laurent.guillou@inserm.fr (L.G.)

**Keywords:** AMD, oxidative stress, lipophenol, PUFA

## Abstract

Age-related macular degeneration (AMD) is a multifactorial pathology and its progression is exacerbated by oxidative stress. Oxidation and photo-oxidation reactions modify lipids in retinal cells, contribute to tissue injury, and lead to the formation of toxic adducts. In particular, autofluorescent pigments such as *N*-retinylidene-*N*-retinylethanolamine (A2E) accumulate as lipofuscin in retinal pigment epithelial cells, contribute to the production of additional reactive oxygen species (ROS), and lead to cell degeneration. In an effort to develop efficient antioxidants to reduce damage caused by lipid oxidation, various natural polyphenols were structurally modified to increase their lipophilicity (lipophenols). In this study, resveratrol, phloroglucinol, quercetin and catechin were selected and conjugated to various polyunsaturated fatty acids (PUFAs) using classical chemical strategies or enzymatic reactions. After screening for cytotoxicity, the capacity of the synthesized lipophenols to reduce ROS production was evaluated in ARPE-19 cells subjected to H_2_O_2_ treatment using a dichlorofluorescein diacetate probe. The positions of the PUFA on the polyphenol core appear to influence the antioxidant effect. In addition, two lipophenolic quercetin derivatives were evaluated to highlight their potency in protecting ARPE-19 cells against A2E photo-oxidation toxicity. Quercetin conjugated to linoleic or α-linolenic acid were promising lipophilic antioxidants, as they protected ARPE-19 cells from A2E-induced cell death more effectively than the parent polyphenol, quercetin.

## 1. Introduction

Nowadays, the interest in natural or synthetic substances to counteract damages produced by oxidative stress (OS) has risen sharply. Especially for cosmetic, agricultural food or pharmaceutical purposes, hydrophobic antioxidants are developed, either to protect lipid food matrices, for better absorption by the skin or to protect cell membranes against lipid peroxidation. In particular, in retina pathologies such as age-related macular degeneration (AMD, the leading cause of vision loss in the elderly), OS plays a significant role in the development and aggravation of the pathology. AMD is a multifactorial, chronic and age-related disease that is influenced by both environmental and genetic factors. In the dry form of the pathology [1,2], oxidative stress is involved in the toxification mechanisms leading to *N*-retinylidene-*N*-retinylethanolamine (A2E) accumulation and degradation. A2E is a toxic *bis*-retinoid derivative, synthesized from vitamin A (*trans*-retinal), condensation, and oxidation. Over the lifespan, A2E and other complex lipids accumulate to form lipofuscin in the retinal pigment epithelium (RPE), ultimately resulting in photoreceptor degeneration [3,4].

Retinal tissue is particularly predisposed to produce reactive oxygen species (ROS) [2] due to its high metabolic rate and high oxygen consumption. ROS can also be produced by A2E photo-oxidation generating singlet oxygen [5]. As A2E displays numerous conjugated double bonds in the structure, it is prone to oxidation and releases toxic metabolites such as endoperoxides and epoxides [5]. In addition, as performed on polyunsaturated fatty acids (mainly present in the membrane of photoreceptors), the lipid peroxidation process is also responsible for A2E cleavage, liberating cytotoxic carbonyl species. These reactive aldehydes, when formed in toxic concentrations will affect the lipid membranes fluidity, and damage DNA and cellular proteins [6,7]. It is hypothesized that toxic A2E, its oxidized metabolites and lipofuscin accumulate when the cellular antioxidant system fails to combat the oxidative-damaged lipids in the photoreceptor cells [8]. Accumulation and photo-oxidation of A2E are known to be one of the critical causes of AMD. Therefore, the reduction of lipid oxidation using lipophilic antioxidants is a promising approach to prevent the progression of AMD [9], similar to the potency of alpha-tocopherol to reduce A2E degradation [3]. Numerous reports uses ARPE-19 cell lines to investigate pharmacologic solutions to AMD and for the identification of new compounds to protect RPE cells against A2E oxidation [10,11,12].

In the quest to limit the OS involved in AMD and to reduce the progression of this pathology, many antioxidants have been proposed. Among them, natural plant polyphenol, which are secondary metabolites that protect plants from microbial pathogens, herbivores as well as solar radiation and, thus, against OS have been well investigated. An example is resveratrol, a phytoalexin that belongs to the stilbene family (3,4′,5-trihydroxystilbene) mainly found in *Vitis vinifera* (*Vitaceae*) stalks and in the roots of *Fallopia japonica* var. *japonica* (*Polygonaceae*), showing antioxidant properties [11] and also anti-neovascularization activity [13] in retinal cells [14]. Also, phloroglucinol, a natural monomer of phlorotannins abundantly present in *Ecklonia cava* (edible brown algae, *Lessoniaceae*) has shown anti-oxidative and anti-carbonyl stress potency in primary RPE cells [15]. Furthermore, numerous flavonoids present in high concentrations in fruits, vegetables and other plant-derived food has been investigated for their antioxidant properties in ARPE-19 and primary human RPE cells [12]. Among the flavonoids, quercetin, a polyphenol of the flavonol family, has received particular attention by the scientific community in the field of ocular diseases [16,17,18], and in protecting RPE cells from A2E oxidation [19,20].

However, no official consent (FDA or EFSA) has been approved for the use of natural polyphenols for the treatment of ocular diseases. Despite promising in vitro efficiency for its bioactivity, the main drawback of polyphenols remains their lack of validation in vivo. Most of the polyphenols have moderate bioavailability due to their physical and chemical properties, which drive the processes of absorption, distribution, metabolism and elimination. For the ones that are water soluble due to their high polarity, it lacks lipophilic properties that could restrict cell penetration and effectiveness in protecting lipophilic system against oxidation. In contrast, the use of polyphenols such as quercetin that show poor solubility in water (a major factor in drug absorption) is affected by low bioavailability and high systemic metabolism during oral administration. Quercetin solubility issues in water or organic solvents questions the efficacy in in vivo administration and hence the validation of its therapeutic effectiveness and potential application. Data suggest that increasing the lipophilicity of (natural) polyphenols may be a useful way to increase their protective effect on lipid matrices, improve their absorption, and ease their formulation for in vivo administration [21,22].

In search for efficient lipophilic antioxidants to reduce OS and A2E oxidation in RPE cells, we prepared a library of new polyphenolic compounds, conjugated to PUFAs. Unlike several reports using lipophilization of polyphenols [23,24], in this study, only one phenolic function of the polyphenol core is used in the conjugation, to preserve the antioxidant properties of interest while increasing the affinity and protection of the lipid molecules such as A2E. PUFAs are preferred to saturated ones, due to their observed benefits in AMD clinical trial [25]. This strategy has already shown promising results with acyl-phloroglucinol [26] and resveratrol [27] derivatives when coupled with docosahexaenoic (C22:6 ω-3, DHA) or linoleic (C18:2 ω-6, LA) acids, for their respective anti-carbonyl and anti-MMP9 activities in cell culture assays. However, only a few examples in the literature reported an improved antioxidant effect of polyphenol lipophilization with PUFA [23] compared to the native polyphenol [28].

Therefore, the purpose of this study was to synthesize novel lipophenol derivatives starting from known antioxidants (resveratrol, phloroglucinol, quercetin and catechin) and to evaluate the effect of the polyunsaturated lipid chain on their capacity to maintain ROS scavenging properties. Both chemical or enzymatic reactions were used to produce mono-acylated lipophenols. The toxicity of the synthesized lipophenols was determined in ARPE-19 cells at various concentrations using the MTT test. The ability of lipophenols to block the accumulation of intracellular reactive oxygen species was examined using a dichlorofluorescein (DCF) probe after H_2_O_2_ treatment. Finally, the protective effects of two quercetin lipophenolic derivatives, that showed no toxicity and the greatest reduction in ROS production were evaluated at various concentrations in cells stressed with A2E and exposed to light (photo-oxidation). Two lipophenolic quercetin derivatives were potent antioxidants that reduced A2E-induced cell death and were more efficient than quercetin itself.

## 2. Materials and Methods

### 2.1. Synthesis of PUFA Lipophenols

#### 2.1.1. General Methods

All solvents were anhydrous reagents from commercial sources. Unless otherwise noted, all chemicals and reagents were obtained commercially and used without purification. Commercial quercetin or (+)-catechin were dried at 100 °C for 48 h under vacuum before use. The reactions were monitored by using TLC on plates that were pre-coated with silica gel 60 (Merck, Kenilworth, New Jersey, USA). The reaction components were visualized by using a 254 nm UV lamp, stained with acidic *p*-anisaldehyde solution followed by gentle heating. Purifications of the synthesized compounds were performed by column chromatography on silica gel 40–63 μm. Melting points (Mp) were determined on a Stuart capillary apparatus and are uncorrected. High resolution mass spectrometry (HRMS) was recorded using electrospray ionization (ESI) or atmospheric solids analysis probe (ASAP) techniques on Q-TOF mass spectrometer.

#### 2.1.2. NMR Characterization

NMR spectra were recorded at 300 or 500 MHz (^1^H) and 75 or 125 MHz (^13^C) using Bruker spectrometers. Chemical shifts are reported in parts per million (ppm, δ) relative to residual deuterated solvent peaks. The NMR spectra were assigned with the help of 2D NMR analyses (COSY, HSQC, and HMBC). The multiplicities reported are as follows: bs = broad singlet, m = multiplet, s = singlet, d = doublet, t = triplet, q = quadruplet, qt = quintuplet, or combinations thereof. For the peak assignments, the following abbreviations were used: Ar = aromatic, TIPS = triisopropylsilyl, CH=CH = aliphatic alkene, *t*Bu = *tert*-butyl. Proton numbering was assigned according to IUPAC nomenclature.

#### 2.1.3. UV Spectroscopy

The UV spectra of compounds was performed in methanol LC/MS grade purchased from Fisher Scientific (France) and were recorded on a UV spectrometer Jasco V-630 apparatus. All spectra were recorded in the range from 500 nm to 230 nm.

#### 2.1.4. Experimental Session

Experimental procedures of chemical/enzymatic synthesis of all intermediates and final lipophenols (phloro-LA (**3**), resv-LA (**8**), cat-3-LA (**9**), quer-3-LA (**13**), quer-3-ALA (**14**), quer-3-DHA (**15**) and quer-7-ALA (**18**)) are fully described in the SI. HRMS analysis, ^1^H-NMR and ^13^C-NMR description and spectrum (Figure S2) are described in SI.

### 2.2. Cell Viability, Cytotoxicity and Antioxidant Activity

#### 2.2.1. Chemicals

All lipophenols were dissolved in dimethylsulfoxide (DMSO) to prepare a stock solution of 80 mM. Hydrogen peroxide solution (H_2_O_2_, 30 wt. % in H_2_O) and 3-(4,5-dimethylthiazol-2-yl)-2,5-diphenyl tetrazolium bromide (MTT) were purchased from Sigma-Aldrich. *N*-retinylidene-*N*-retinylethanolamine (A2E) was synthesized as previously described by Parish et al. in 1998 [29]. 2′,7′-dichlorofluorescin diacetate (DCFDA) was purchased from SIGMA-Aldrich (Saint-Quentin, France) and dissolved in DMSO to prepare stock solution at 20 mM. All stock solutions of lipophenols, A2E and probe were stored at −20 °C in the dark.

#### 2.2.2. Cell Culture

ARPE-19 cells were obtained from ATCC (USA) and were grown in Dulbecco’s Modified Eagle’s Medium (DMEM)/Ham F12 (GIBCO) containing 10% *v*/*v* fetal bovine serum (FBS) and 1% *v*/*v* penicillin/streptomycin under atmospheric humidified air (95%)/CO_2_ (5%) at 37 °C. For experimental cell seeding and sub-culturing, the cells were dissociated with 0.25% trypsin-EDTA, resuspended in the culture medium and then plated at 1–3 × 10^5^ cells/mL. ARPE-19 cells were cultured and used up to 15 passages maximum.

#### 2.2.3. Cell Viability

Cell viability was determined by MTT colorimetric assay. The cells were incubated for 2 h with MTT reagent (0.5 mg/mL). During this incubation time, dehydrogenases of the living cells reduce the MTT to insoluble purple formazan. The absorbance at 570 nm and 655 nm of individual wells of the cell was measured using a microplate reader (BioRad 550, USA). The absorbances of the compounds tested does not interfere with the absorbance at 570 and 655 nm. The percentage of the viable cells was calculated as [(OD570 sample − OD655 sample)/(OD570 control − OD655 control)] × 100%.

#### 2.2.4. Cytotoxicity of Lipophenols

ARPE-19 cells were plated into 96-well plates (4 × 10^4^ cells/well) and cultured for 24 h to reach confluence before lipophenol treatment. The cell cultures were treated with serum free medium containing the lipophenols at different concentrations (0–160 µM) for 24 h. Control cells were incubated with DMSO (0.2%). The viability of the cells was determined using MTT colorimetric assay as described above and expressed in percentage of viable cells normalized with control conditions in the absence of lipophenols. When a dose-dependent toxicity was observed, CC_50_ was calculated.

#### 2.2.5. Protection of Lipophenols against ROS Production

ROS activity was measured in ARPE-19 cells with dichlorofluorescein diacetate (DCFDA) reagent. DCFDA is deacetylated by cellular esterases to dichlorofluorescein (DCFH), which can then be oxidized by ROS into the fluorophore 2′,7′–dichlorofluorescein (DCF). ARPE-19 cells were plated into black, optically clear bottom 96-well plates (4 × 10^4^ cells/well) and cultured for 24 h to reach confluence before the drug treatment. The cell cultures were incubated with 2 µM of DCFDA for 45 min in DMEM/F12 medium without phenol red + 1% FBS. The cells were rinsed and incubated with the medium containing lipophenols at different concentrations (0–80 µM) for 1 h. Then, H_2_O_2_ was added to a final concentration of 600 µM for 4 h, followed by the measurement of DCF production by fluorescence spectroscopy with excitation wavelength at 485 nm and emission wavelength at 535 nm. The fluorescence of the compounds tested does not interfere with DCFDA signal. Control cells were incubated with DMSO (0.2%) ± DCFDA ± H_2_O_2_. The percentage of ROS produced was calculated as [(fluorescence of sample)/(fluorescence of control)] × 100%. The results are expressed in percentage of ROS produced normalized with control conditions in the absence of lipophenol and presence of stressor. When a dose-dependent inhibition was observed, IC50 was calculated.

#### 2.2.6. Protection of Lipophenols against Photo-Oxidized A2E Toxicity

ARPE-19 cells were plated into 96-well plates (4 × 10^4^ cells/well) and cultured for 24 h to reach confluence before lipophenol treatment. The cell cultures were treated with serum free DMEM/F12 medium without phenol red containing lipophenols at different concentrations (0–80 µM) for 1 h. Then A2E was added to a final concentration of 20 µM for 6 h before rinsing with medium. Control cells were incubated with DMSO (0.2%) with or without A2E. The cells were exposed to intense blue light (4600 LUX) for 30 min to induce phototoxicity of A2E and incubated at 37 °C. The cell viability was determined 16–20 h later using a MTT colorimetric assay. Results are expressed in percentage of viable cells normalized with control conditions in the absence of lipophenols and stressor. When a dose-dependent efficiency was observed, IC_50_ was calculated.

#### 2.2.7. Statistical Analysis

The data are presented as means ± SD determined from at least three independent experiments. In each experiment, all conditions were done at least in quadruplicate. Statistical analysis was performed by Student’s *t*-test for Gaussian distributions or non-parametric Mann-Whitney’s test for non-normal distributions (the normality of distributions was highlighted with a Shapiro-Wilk test) and differences with *p*-values < 0.05 were considered as statistically significant. CC_50_ and IC_50_ were calculated using GraphPad Prism version 5.03 and linear regression.

## 3. Results

### 3.1. Chemical and Enzymatic Synthesis of PUFA Lipophenols

Seven lipophenols were synthesized using natural phenolic compounds, phloroglucinol, resveratrol, quercetin and catechin. First, linoleic acid (C18:2, ω-6; LA) was selected to compare different family of phenolic compound. To better appreciate the impact of the lipid chain, additional fatty acid such as linolenic (C18:3, ω-3; ALA) and docosahexaenoic (C22:6, ω-3; DHA) acids were linked to the quercetin series and compared to the LA analogue. Finally, the impact of the PUFA position on the quercetin core was evaluated by the synthesis of both quercetin-7-ALA (quer-7-ALA) and quercetin-3-ALA (quer-3-ALA).

#### 3.1.1. Phloroglucinol and Resveratrol Conjugates

##### Synthesis of Phloroglucinol-LA

**Figure 1:**
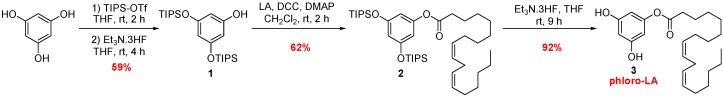
Synthetic pathway to access phloroglucinol-LA.

Phloroglucinol-LA derivative was synthesized following the strategy developed by Crauste et al. [26] (Figure 1) using silylated protecting group for a single PUFA coupling. Briefly, hydroxyl groups of the phloroglucinol are protected by triisopropylsilyl (TIPS) groups using triflate reagent (TIPS-OTf) and diisopropylethylamine (DIPEA) as a base to obtain a tri-protected derivative. One TIPS function was deprotected by carefully following the reaction on TLC using triethylamine trihydrofluoride (Et_3_N.3HF) and afforded the mono deprotected compound **1**. The coupling reactions between the protected phloroglucinol and the linoleic acid (LA) was initiated using dicyclohexylcarbodiimide and dimethylaminopyridine (DCC/DMAP) as coupling reagents to access to protected lipophenol **2**. Final deprotection of TIPS protecting groups by Et_3_N.3HF in dry tetrahydrofuran (THF) yielded final lipophenol compound without ester degradation (**3**, phloro-LA).

##### Synthesis of Resveratrol-4′-LA

**Figure 2:**
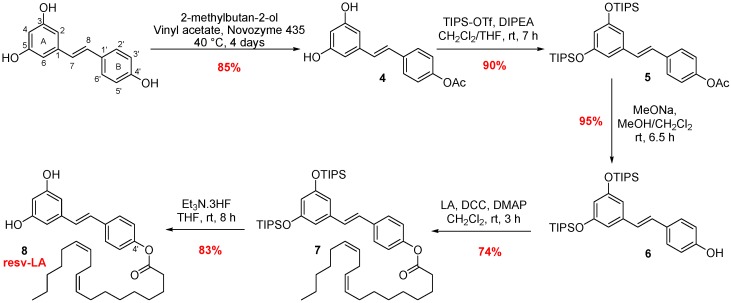
Synthetic pathway to access resveratrol-4′-LA.

The work of Vlachogianni et al. [30] show better radical scavenging activity of 4′-acetylated resveratrol compared to 3-acetylated analogues; we decided to link the fatty acid at the 4′ position of resveratrol. Another argument for choosing this coupling position was to keep the resorcinol moiety accessible, which appears to be involved in trapping steps of toxic aldehyde in phloroglucinol or flavonoid structures [15,31]. The synthesis of resveratrol-4′-LA was performed as previously described [27] (Figure 2) using an enzymatic chemical strategy allowing to isolate only the 4′-acylated analogue. Using a freshly (and not recycled one) supported lipase *Candida antartica* (CALB, Novozyme 435), a first acetyl group was regio-selectively introduced at the 4’ position in good yield (85%) without any acetyl derivatives in 3 or 5 positions. The resv-LA (8) was then obtained in four steps; hydroxyl silyl protection, 4′-acetate enzymatic deprotection, fatty acid coupling and a final silyl deprotection, with 52% overall yield in 4 steps.

#### 3.1.2. Catechin and Quercetin Conjugates

##### Synthesis of Catechin-3-LA

**Figure 3:**
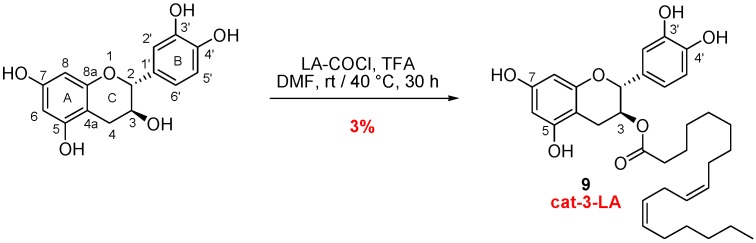
Synthetic pathway to access catechin-3-LA.

In catechin structure, the position of the fatty acid was selected according to the study of Hong et al. [32]. Radical scavenging properties seem to be less affected by acylation at the 3 or 7 position compared to acylation of the catechol moiety. Position 3 was thus selected to design catechin lipophenol. In order to avoid multiple step synthesis, including protection/deprotection strategy [33,34] and access quickly to the desired catechin-3-LA, we investigate the preferential reactivity of aliphatic hydroxyl under acidic conditions, compared to the phenolic hydroxyls. Under undissociated form, aliphatic hydroxyl should be more nucleophilic than the phenolic ones [32]. Coupling step was thus realized according to Uesato et al. [35] using TFA and linoleoyl chloride freshly prepared (Figure 3). Unfortunately, mono-acylated cat-3-LA (**9**) was isolated in weak yield using this process, but in sufficient amount to evaluate its antioxidant potency. Confirmation of the structure and verification of introduction of the LA moiety in position 3 has been validated according to Park et al. [36] by a ^1^H NMR shift of proton H3 (from 3.8 ppm for commercial (+)-catechin to 5.2 ppm for 3-R-derivatives).

##### Synthesis of Quercetin-3-LA, 3-ALA, 3-DHA and 7-ALA

Quercetin is rapidly metabolized after administration, and the 3-conjugates are commonly found as main metabolites (3-glucuronide) [37]. Selecting the position 3 to link the PUFA may affect the metabolism of quercetin leading to its elimination. A chemical/enzymatic strategy that avoids multistep synthesis was investigated to access the quercetin-3-LA derivative. To evaluate the impact of the PUFA chains during in vitro assays, DHA (the main PUFAs present in photoreceptor membrane) and ALA analogues were also synthesized.

**Figure 4:**
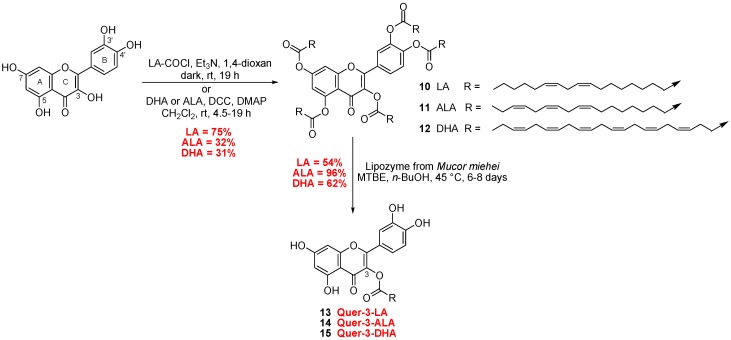
Synthetic pathway to access quercetin-3-LA, 3-ALA and 3-DHA.

To access 3-*O*-acyl-quercetin derivatives, two esterification methods were performed (Figure 4). A first penta-*O*-acylation was realized either using fatty acyl chloride (for quer-3-LA strategy, compound **10**, 75%) or a less fatty acid consuming method using Steglich esterification (for ALA and DHA coupling compound **11** and **12**). Then, the regioselective deprotection of PUFA at the 5, 7, 3’ and 4’ position was performed using supported lipase from *Mucor miehei* (immobilised, Lipozyme^®^ IM). This process has been previously described for the synthesis of saturated 3-*O*-acyl-quercetin [38], but has never been applied for the synthesis of unsaturated analogues. This enzymatic process is completed over 6 to 8 days depending on the lipid chain and afforded desired compounds quer-3-LA (**13**), quer-3-ALA (**14**) and quer-3-DHA (**15**) with moderate to good yields.

**Figure 5:**
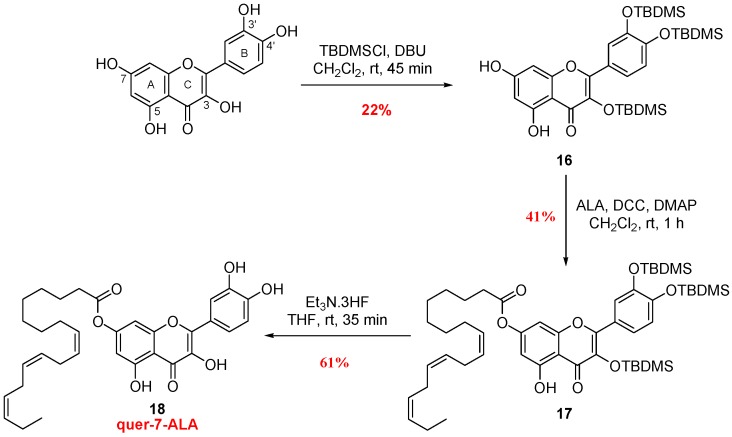
Synthetic pathway to access quercetin-7-ALA.

To end, we investigated the effect of acylation of the PUFA (using ALA) at the 7 position compared to the 3 position, since both positions should be less invested in radical scavenging properties. The synthesis of quer-7-ALA was performed in three steps from the commercial quercetin (Figure 5). Protection of positions 3, 3′ and 4′ with *tert*-butyl dimethylsilyl ethers (**16**) allowed the introduction of ALA chain via a Steglich esterification (**17**) on position 7, without touching the 5 position involved in hydrogen bond. Final deprotection of the silyl groups afforded quer-7-ALA (**18**) with 61% yield.

**Figure 6:**
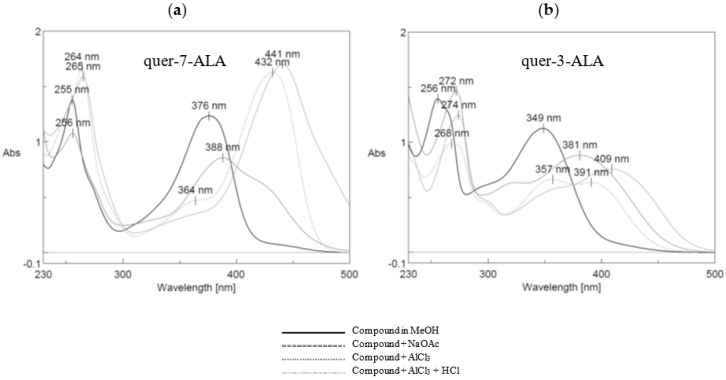
UV spectra of quer-7-ALA (**a**) and quer-3-ALA (**b**) under different conditions: in MeOH, with NaOAc, with AlCl_3_ and with AlCl_3_ + HCl.

To carefully validate the position of ALA derivative on both 7- and 3-isomers (**18** and **14**), ^1^H and ^13^C NMR shift between both compounds and natural quercetin (Table 1) were first analyzed using quercetin as reference. Higher chemical shift variations of H6, H8 and C7 signals were obtained with 7-ALA isomer compared to the 3-ALA derivative, whereas shielding was observed for C3 signal only in quer-3-ALA analysis. Since position of the fatty acid could not be deeply confirmed by Heteronuclear Multiple Bound Connectivity (HMBC) NMR analysis, characteristic UV spectrum of flavonoid was used to confirm the position of acylation [39]. The UV spectrum of quer-7-ALA exhibits two absorption maxima at 255 and 376 nm (band II and I Figure 6a). A bathochromic shift of the first maximum (band II—characteristic of the hydroxyl benzoic group) upon the addition of sodium acetate (AcONa) was not observed in contrast with the case of quer-3-ALA (Figure 6b, from 256 nm to 272 nm). Since the weak base AcONa is able to deprotonate the more acidic 7-OH [40], this experiment confirmed the presence of a substituent at the 7-position on the quer-7-ALA and its absence on the quer-3-ALA. The absence of catechol substitution was then confirmed in both structures using first AlCl_3_ treatment (bathochromic shift of band I characteristic of the cinnamic system is observed due to aluminum chelation with the catechol diol) and then upon addition of HCl (cleavage of the unstable complex leading to a hypsochromic shift of the same band). To end, the 5-position of both structures was not acylated according to AlCl_3_ complex formation (band II, bathochromic effect), which is stable after addition of HCl.

### 3.2. Toxicity of Lipophenols in ARPE-19 Cells

First, the toxicity of synthesized lipophenols was evaluated in a RPE cell line (ARPE-19). ARPE-19 cell line is widely used in AMD studies for testing potential new drug candidates [10,26,42]. Even if they do not express the exact cellular characteristics of primary RPE cells, they display similar physiological properties than RPE cells in vivo [43]. All the compounds were tested at various concentrations in the range of 0–160 µM (Table 2, ARPE-19 CC_50_). All phloroglucinol and resveratrol derivatives showed no toxicity up to 160 µM in this cell line. Natural catechin showed no apparent toxicity, whereas its lipid derivative (**9**, entry 6) displayed a slight mortality at high concentrations (CC_50_ = 148.63 µM). Natural quercetin showed a moderate toxicity (entry 7) that was increased or decreased by the presence of the lipid component, depending on the nature of the PUFA and the position in the polyphenol moiety. For quer-3-LA (entry 8), no toxicity was observed up to 160 µM, whereas a slight toxicity was observed for the quer-3-DHA (entry 9) and even a high toxicity (IC_50_ = 69.44 µM) was displayed by quer-3-ALA (entry 10). Surprisingly, the toxicity disappeared with the same lipid (ALA), when introduced in position 7 instead of 3 (entry 11).

### 3.3. Inhibition of ROS Production by Lipophenol Pre-Treatment in ARPE-19 Cells

The antioxidant potential of the lipophenols was evaluated with a ROS inhibition test. Briefly, lipophenols were incubated 1 h in cells before addition of H_2_O_2_ stressor at a high concentration (600 µM). The level of produced ROS was measured with a fluorescent probe DCFDA [44]. The results are presented in Table 2 (ROS production IC_50_) and expressed as the concentration of lipophenol required to reduce 50% of the ROS produced in H_2_O_2_ stress conditions. For the phloroglucinol lipophenol, (entry 2) the antioxidant potency was lower than its natural analogue. As with the resveratrol-4′-LA (entry 3), antioxidant properties were not conserved since IC_50_ was higher than 80 µM. For the catechin analogue (entry 6), ROS inhibition seems to be in the same range than the natural catechin (IC_50_ = 10.61 µM versus 5.93 µM). When considering the quercetin analogues, the antioxidant potency of the 3-LA, 3-DHA and 3-ALA decreased by the presence of the lipid with equivalent potency, regardless of the lipid moiety introduced (IC_50_ from 52 to 62 µM). Finally, quer-7-ALA (entry 11) displayed the best antioxidant activity of the quercetin analogues with IC_50_ = 9.44 µM, and was more potent than its homologue 3-ALA.

### 3.4. Lipophenols Protected RPE Cells from A2E Photo-Oxidation Toxicity

**Figure 7:**
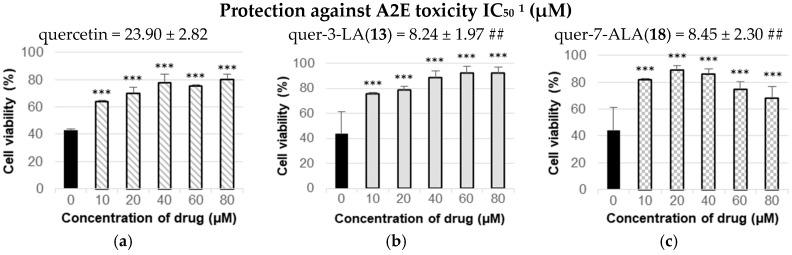
Protecting effect of quercetin (**a**), quer-3-LA (**b**) and quer-7-ALA (**c**) against photo-oxidized A2E, represented as cell viability (%) in function of the used drug concentration (µM) i.e., cells exposed to A2E and non-treated. Results are expressed in mean ± SD and are from at least n = 3 independent experiments. Results are normalized with 100% viability for non-treated and non-exposed to A2E toxicity control cells. ^1^ Concentration of lipophenol needed to inhibit 50% of the toxicity induced by photo-oxidized A2E. Mann-Whitney’s test, # *p* < 0.05, ## *p* < 0.01, ### *p* < 0.001, versus natural quercetin. Mann-Whitney’s test, * *p* < 0.05, ** *p* < 0.01, *** *p* < 0.001, versus non-treated cells exposed to photo-oxidized A2E.

It is well known that when human RPE is irradiated at an excitation maximum in the blue region of the spectrum, A2E generates various reactive forms of oxygen in addition to endoperoxides, epoxides and furanoid moieties [45]. Since this phenomenon plays a crucial role in the progression of AMD, two candidates were selected to evaluate their ability to protect ARPE-19 cells from toxicity of photo-oxidized A2E, based on their absence of toxicity and their efficiency to reduce ROS production. Although catechin-3-LA displayed a good antioxidant activity, it has not been selected due to its cytotoxicity on ARPE-19 starting from 80 µM and its poor accessibility. Among quercetin lipophenols that did not show any toxicity up to 160 µM, quer-3-LA and quer-7-ALA were further evaluated and compared to quercetin, as they showed the most interesting antioxidant properties in the ROS inhibition assay. In the test, the drugs were pre-incubated for 1 h prior addition of A2E. After 6 h of incubation, the medium was rinsed and the cells were exposed to intensive blue light for 30 min before overnight incubation. Results are shown in Figure 7. The two quercetin derivatives (compounds **13** and **18**) showed similar protective effect on ARPE-19 cells when exposed to photo-oxidized A2E. They were able to decrease the toxic effect of A2E from 50% at 8 µM only and were able to protect up to 90% of cells at higher concentrations. The protection of both flavonoid lipophenols was higher than the one produced by quercetin treatment.

## 4. Discussion

Oxidative stress is one of the major toxic mechanisms responsible for age-related RPE damages. Many studies have demonstrated that accumulation of lipid deposit called lipofuscin, generates reactive oxygen species through phototoxicity in RPE cells [3]. Oxidative stress caused by photo-oxidation of *bis*-retinoid A2E (a component of lipofuscin) is known to be a critical cause not only in AMD progression, but also in genetic macular degeneration such as Stargardt disease. In this context, the reduction of lipid peroxidation using lipophilic antioxidants derived from natural antioxidant molecules was a logical choice to reduce deleterious oxidative stress. Four natural phenolic derivatives were selected in this study for their potent antioxidant properties or for their previous evaluation in RPE cell assays: phloroglucinol [15], resveratrol [14], catechin, and quercetin [46,47]. Among them, natural quercetin has been largely evaluated in the field of retina dystrophies for its direct or indirect antioxidant properties: Cao et al. [18] demonstrated the ability of quercetin to protect human RPE cells from oxidative stress in vitro via suppression of pro-inflammatory cytokines and inhibition of apoptosis pathway. Hytti et al. [48] performed in vitro evaluation of quercetin after exposure of cells to the lipid peroxidation end product 4-hydroxynonenal (HNE) and found that quercetin was able to increase cell viability under these conditions but was also able to reduce inflammatory mediators. Recently Wang et al. [20] demonstrated the ability of quercetin and cyanidine-3-glucoside to protect RPE cells against light damage by inhibiting the photo-oxidation and photo-degradation of *bis*-retinoid.

To increase their protection of lipid molecules, and to protect them from polyphenol loss through metabolic and excretion process, the four selected phenolic derivatives were esterified at one phenolic position with different PUFAs. In this investigation, a single PUFA was incorporated in the phenolic backbone to minimize the interference of the antioxidant properties. The potency to limit ROS production and protect cells against A2E photo-oxidation was evaluated in ARPE-19 cells.

### 4.1. Chemical and Enzymatic Synthesis of Lipophenols

The preferred position of the lipid substitution was position 4’ for the resveratrol backbone, since some studies showed the best antioxidant activities when the phenols at the 3 and 5 positions were not functionalized [30]. When considering (+)-catechin backbone, position 3 was linked to the lipid moiety since phenolic functions in position 3 was found to be the less important for scavenging activity of ROS [32]. Furthermore, the same choice was made for quercetin derivatives. Position 3 was esterified by different PUFAs, either ω-3 or ω-6 (LA, ALA and DHA), to compare their contribution to the antioxidant activity. Position 7 was also substituted by ALA chain to investigate the impact of the fatty acid position on the quercetin series.

To incorporate only one lipid part, chemical strategies were chosen either using protection/deprotection steps of the phenolic functions or using stereo-selective enzymatic reactions. Using a previous chemical strategy [26] and by TIPS protection of the phenolic functions, the synthesis of phloroglucinol derivative was performed in four steps to access desired compound phloro-LA (**3**) with 34% overall yield. When considering resveratrol series, the synthesis performed as described by Shamseddin et al. [27] allowed to isolate the desired resv-4′-LA (**8**). The global yield of the strategy was improved due to the amelioration of the 4′-enzymatic acetylation using a non-recycled supported lipase *Candida antartica* (CALB, Novozyme 435). For the catechin series, a single step synthesis was performed, but had weak yield. Finally, two different pathways were developed to access 3- or 7-quercetin derivatives substituted with different fatty acids. The synthetic pathway used to access 3-PUFA quercetins, quer-3-LA (**13**), quer-3-ALA (**14**) and quer-3-DHA (**15**), starts with a full penta-acylation of the phenolic functions of the quercetin followed by a stereoselective enzymatic cleavage using the supported lipozyme from *Mucor miehei*. This process was previously described for the synthesis of saturated 3-*O*-acyl-quercetins [38], which showed the selectivity of the enzyme (avoiding the 3-position) using saturated fatty acids. We validated for the first time the methodology using PUFA (LA, ALA and DHA). Only recently, Carullo et al. [49] described another enzymatic pathway to access quercetin-3-oleate derivative in a single step from quercetin and oleic acid (C18: 1, ω-9) by using pancreatic porcine lipase. This methodology is a promising work and may be adapted in future to our derivatives using polyunsaturated fatty acids such as DHA.

### 4.2. How Does PUFA Introduction Influence Toxicity of Phenols in ARPE-19 Cells?

**Figure 8:**
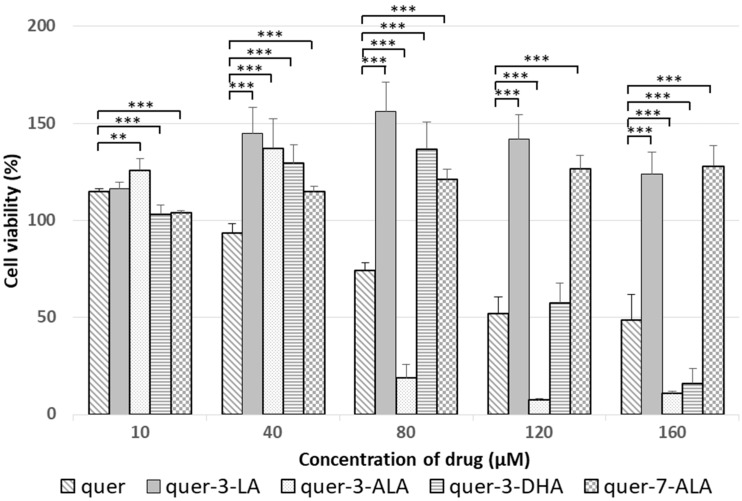
Cytotoxicity assay: representation of ARPE-19 cell viability in presence of PUFA quercetin derivatives; natural quercetin (**quer**), quer-3-LA (**13**), quer-3-ALA (**14**), quer-3-DHA (**15**) and quer-7-ALA (**18**) expressed as percentage of viability in function of drug concentration introduced on cells. Results are expressed in mean ± SD and are from at least n = 3 independent experiments. Results are normalized with 100% viability for non-treated control cells. Student’s *t*-test or Mann-Whitney’s test, * *p* < 0.05, ** *p* < 0.01, *** *p* < 0.001, versus natural quercetin (**quer**) at equivalent concentration.

Cytotoxicity evaluation of the derivatives in this study showed that the LA introduced in phloroglucinol and resveratrol series had no enhanced toxicity in ARPE-19 cells compared to their natural homologue. Concerning the catechin derivative, a small toxic effect due to the LA introduction at position 3 was noted. For quercetin derivatives, it was found that the nature of the lipid moiety was significant. When substituted with LA (position 3), an important protective effect of the lipophenol was observed in the cells (150% survival at 80 µM, Figure 8), whereas natural quercetin displayed a CC_50_ of 111.45 µM and showed no protective effect. When substituted with DHA at the same position, the protective effect of lipophenol was reduced but still present at lower concentration before showing toxicity signs resulting in shifted CC_50_ (134.10 µM) compared to natural quercetin (Figure 8). These protective effects of lipophilic quercetins could be explained by an enhanced cell penetration of the compounds due to the association of a lipid moiety to the flavonoid [50] and/or the action of the PUFA itself. The mechanism by which lipophenol may increase cell viability could be through enhancement of the cell integrity and/or the induction of cell proliferation. Indeed, beneficial effects of DHA as an antioxidant and protective agent have been already described [51,52] in RPE cells, in patients, and rats. Surprisingly, when substituted with ALA at position 3, an enhanced toxicity was observed with a CC_50_ = 69.44 µM and an acute toxicity up to 80 µM. This result shows that the nature of the lipid is important to observe a protective effect of 3-PUFA quercetin derivatives on RPE cells. The different toxicity profiles obtained using ALA (ω-3) derivative compared to LA (ω-6) and DHA (ω-3) may be highlighted by the different roles and functions of each PUFA and PUFA metabolites, which are represented as cell mediators and signaling molecules in the cell [53,54]. One can hypothesize that spatial conformation of the lipophenol may also play a role resulting in different stability in the cells. Finally, toxicity evaluation of quer-3-ALA versus quer-7-ALA brings to light the importance of the substituent position on the backbone. Indeed, the toxic effect observed with quer-3-ALA (CC_50_ = 69.44 µM) disappears for the 7-ALA isomer. Furthermore, quer-7-ALA displays a protective effect on cells with no evidence of toxicity and increased viability (128%, Figure 8) up to 160 µM.

### 4.3. Antioxidant Properties of Lipophenols: ROS Scavenging and Protection against Oxidized A2E

For most of the series (phloroglucinol, resveratrol and quercetin), an important loss of ROS scavenging properties was observed when one lipid is associated to the phenolic backbone. For the phloroglucinol derivative, phloro-LA is nine times less potent than its natural homologue. Regarding the resveratrol series, this loss is even more important as only 40% of ROS is scavenged by resv-LA at 80 µM (Figure S1). The same result was observed by Oh et al. [55] in non-cellular DPPH and ABTS assays, where substitution of hydroxyl group(s) appeared to negatively affect the antioxidant activity of the resveratrol derivatives. Natural quercetin displayed a high antioxidant activity with an IC_50_ in the same range compared to phloroglucinol, resveratrol and (+)-catechin. When position 3 was substituted by one LA moiety (quer-3-LA), ROS scavenging properties were highly decreased but still more potent than its homologues phloro-LA and resv-LA. Mainini et al. [24] evaluated fatty acid-quercetin derivatives using either stearic, oleic, linoleic or linolenic acids to form penta-(3,5,7,3′,4′), tetra-(3,7,3′,4′) or tri-(7,3′,4′) esters and also demonstrated, in non-cellular assay, a systematic decrease of antioxidant properties of the lipophenol derivatives associated with the number of phenolic function esterified.

This subsiding activity has been frequently observed in DPPH or ABTS non-cellular antioxidant assays performed on lipophilized polyphenols [24,56,57,58]. However, different conclusions have been described from experiments carried out in a bulk oil system, where, for example, resveratrol ester is able to outperform the antioxidant capacity of the original resveratrol [59]. By performing ROS inhibition assay in a cellular media, one could anticipate that the increase in cell penetration (due to increased lipophilicity) confers to the lipophilic derivatives a more potent effect than their native polyphenol. However, this was not observed. This decreased ROS scavenging potency could be explained by the loss of a phenol function due to introduction of the fatty acid but could not be the only explanation since lipophilized phlroridzin, rutin or isoquercitrin have shown reduced antioxidant capacity even when the lipid part was localized on the sugar hydroxyl, which does not participate in the ROS scavenging mechanism [57,58].

Interestingly for the catechin series, lipophenol cat-3-LA displays the same range of antioxidant activity as the natural compound, suggesting that aliphatic hydroxyl in position 3 was not essential for ROS scavenging activity. The same conclusion was highlighted by Jin et al. in 2005 [60] by evaluating radical scavenging activity of 3-lauroyl; 3′,4′-dilauroyl- and 3,3′,4′-trilauroyl-(+)-catechins in a DPPH test. They showed a conserved antioxidant potential of the 3-lauroyl derivative compared to natural catechin. Perera et al. [61] recently evaluated the antioxidant properties of epigallocatechin gallate (EGCG) tetra-esters substituted with different fatty acid chains and observed the highest DPPH scavenging activity for EGCG-DHA derivative. The group also evaluated the protective effect of their derivatives in various food model and biological systems and concluded on the major role of the lipophilicity and chain length of fatty acids in the antioxidant capacity of the derivatives.

Not taking into account their toxicity profile, the most interesting antioxidant lipophenol conjugated to LA was cat-3-LA > quer-3-LA > phloro-LA >> resv-4′-LA. In spite of this observation, cat-3-LA displayed a mild toxicity at concentrations higher than 80 µM compared to quer-3-LA, which showed a protective effect on cells even at high concentrations (up to 160 µM) on toxicity assay (Figure 8). The choice of the fatty acid was also evaluated using quercetin backbone leading to 3-LA, 3-DHA and 3-ALA derivatives. By comparing their antioxidant profile, it can be concluded that their efficiency is in the same range (IC_50_ approximately 60 µM), regardless of the type of lipid part introduced. The presence of a PUFA moiety in position 3 systematically reduced ROS scavenging activity of quercetin independently of the nature of the PUFA (LA, ALA or DHA). Finally, the comparison of quer-3-ALA with quer-7-ALA led to the conclusion that position 3 is more important than the 7 position to display a high antioxidant effect of the flavonoid. Quer-7-ALA was 6 times more potent to scavenge ROS than its analogue 3-ALA. This result is in line with the literature reporting that positions 5 and 7 are less important for ROS scavenging [23,24].

The two most promising compounds in terms of non-toxicity and ROS scavenging properties were evaluated for their capacity to protect cells from photo-oxidized A2E and compared to native quercetin efficiency. Quercetin was able to rise a maximum of 78% of cell viability when used at 40 µM. This result is in line of what was observed by Sparrow’s group, who used quercetin in the same cell model [20]. In our investigation, quer-3-LA and quer-7-ALA were able to efficiently increase cell viability in the presence of toxic A2E and displayed similar protective effect with IC_50_ in the same range (8.24 and 8.45 µM, respectively). A maximum of 88–89% of cell viability was observed between 20 and 40 µM concentrations. Same level of protection was observed previously using resveratrol or piceatannol at 60 µM in ARPE-19 cells treated by photo-oxidized A2E [11]. However, compared to our procedure (1 h of lipophenol pretreatment), cells were treated twice with resveratrol or piceatannol for a duration of 3 days before A2E stress. As recently observed by Kim et al. [19] with 3-quercetin glycoside, the esterification of the phenol function in position 3 was compatible with the protection against A2E induced toxicity. Kim’s group showed efficiency of 3-quercetin glycoside to protect RPE cells (~90% of cell viability at 100 µM) and Balb-c male mice photoreceptors against blue light damages. In our study, the efficiency of quercetin lipophenols was also comparable with the report using Norbixin [62]. This natural lipid diapocarotenoid shown high protection (~90–100% at 20 µM) against A2E illuminated in porcine primary RPE cells, and was also able to reduce A2E in Abca4 −/− Rdh8 −/− mice treated orally for three months. The protection we highlighted with quer-3-LA and quer-7-ALA appears to be slightly more significant than those of curcumin derivatives that showed a maximum rise of 60–70% of cell viability (3 days incubation at 20 µM) in ARPE-19 cells subjected to photo-oxidized A2E [63].

Interestingly, in our study, quer-3-LA and quer-7-ALA both are three times more potent than the natural quercetin (IC_50_ = 23.90 µM). This result contrasts with the ROS inhibition test; indeed, in A2E photo-oxidized assay, natural quercetin displayed a lower antioxidant activity than the two lipophenolic derivatives. Moreover, even if quer-3-LA was less potent than its analogue quer-7-ALA in the ROS test, it displayed the same protecting efficiency towards cells exposed to light-induced A2E. Oh et al. [59] observed similar contrasting results, where Resv-DHA displayed no hydrogen peroxide scavenging activity whereas it showed similar inhibition of DNA scission induced by hydroxyl radical compared to natural resveratrol. This result confirms the importance of selecting the test to evaluate the potency of antioxidants. More than one specific assay is needed to evaluate the antioxidant properties of a newly found compound, as highlighted by Oh et al. [59] on lipophilized resveratrol derivatives or by Viskupicova and Maliar [57] on lipophenolic rutin derivatives. Moreover, the antioxidant activity of polyphenols undergo different mechanisms, including direct action of drug by oxygen radical scavenging but also indirect action through enzymes induction [64] (i.e., Catalase, ERK pathway, NRF2-KEAP-1) as mentioned by Kang et al. for phloroglucinol [65] or de la Lastra et al. for resveratrol [66]. In this study, the ability of a derivative to protect RPE cells against oxidative stress induced by light exposure should be more significant than the most common non-cellular assays (i.e., DPPH or ABTS) for the selection of new antioxidant derivatives for the development of pharmacological solution to reduce progression of AMD and Stargardt pathology [4].

Taken together, the different evaluations performed in this work highlight quer-7-ALA as a promising compound for further evaluations against AMD oxidative stress. Nevertheless, it has been demonstrated that a double carbonyl and oxidative stress (COS) stress is involved in toxic mechanisms, leading to photoreceptor death in AMD and Stargardt disease. As previously described using lipophenolic phloroglucinol, the resorcinol moiety of the A ring of the flavonoid backbone might be important to display protection against carbonyl stress [26]. As a consequence, quer-3-LA, having free hydroxyls on the A ring, might also be an interesting lipophenolic derivative to confer both antioxidant and anti-carbonyl stress properties in a single molecule.

## 5. Conclusions

Most of the ROS inhibition tests performed on lipophenol derivatives as described in the literature are performed on non-cellular assay and frequently showed a decrease in the antioxidant properties of lipophenols. In this work, the test was performed in ARPE-19 cells, where the polyphenol ROS scavenging activity was mostly reduced following the PUFA introduction in the structure. However, we further highlighted the beneficial effect of lipophenol in increasing lipid protection during A2E photo-oxidation of the RPE cells. The importance of the position of the PUFA was demonstrated in the flavonoid backbone that influenced either toxicity or activity. Among the lipophenols tested, quer-7-ALA displayed substantial protection against A2E toxicity in ARPE-19 cells. The levels of cellular protection were comparable to the one of a lipid antioxidant showing A2E reduction in a mouse model (Norbixin) [62] or a flavonoid demonstrating in vivo protection against retinal degeneration induced by blue light exposure [19]. Quer-3-LA also possess interesting antioxidant properties, which combined with its anti-carbonyl stress activity would be interesting to further evaluate in a mouse model of retina pathology. We hypothesize the process by which these flavonoid lipophenols improved their antioxidant activities compared to quercetin, which must pass through cell penetration or through membrane incorporation. To better address this question, it is essential to investigate the stability and pharmacokinetics of such derivatives in future. In addition, the mechanisms of the lipophenol protection must be explored to identify the real target of these new lipophilic antioxidants and determine their future potential in other retina cell models. In closing, this study highlights the importance of implementing a variety of cellular assays to evaluate antioxidant potency.

## 6. Patents

A part of the molecule presented in this work are described in the patent: Lipophenolic flavonoid derivatives useful to reduce carbonyl and oxidative stresses (COS); EP18305957.5, July 2018.

## Figures and Tables

**Table:** 

Compound	*δ* (H-6)	*δ* (H-8)	*δ* (H-2′)	*δ* (H-5′)	*δ* (H-6′)
quer	6.18	6.39	7.73	6.89	7.63
quer-3-ALA (14)	6.23	6.42	7.33	6.88	7.27
quer-7-ALA (18)	**6.46**	**6.81**	7.77	6.88	7.65

**Table:** 

Compound	*δ* (2-C)	*δ* (3-C)	*δ* (4-C)	*δ* (4a-C)	*δ* (6-C)	*δ* (7-C)	*δ* (8-C)
quer	148.03	137.20	177.33	104.50	99.28	165.73	94.45
quer-3-ALA (14)	158.42	**131.45**	177.21	105.29	100.14	166.25	95.04
quer-7-ALA (18)	149.31	138.02	177.62	108.49	104.82	**157.19**	101.86

**Table 2:** Cytotoxicity and antioxidant potential of lipophenols against induced ROS production.

Entry	Compound	ARPE-19 CC_50_^1^	ROS production IC_50_^2^
1	Phloroglucinol	>160	10.25 ± 3.44
2	Phloro-LA (**3**)	>160	>80
3	Resveratrol	>160	7.40 ± 0.51
4	Resv-4′-LA (**8**)	>160	>>80
5	(+)-catechin	>160	5.93 ± 0.06
6	Cat-3-LA (**9**)	148.63 ± 6.61	10.61 ± 4.41 ◆◆◆
7	Quercetin	111.45 ± 4.92	6.82 ± 0.73
8	Quer-3-LA (**13**)	>160	52.25 ± 10.84 ***
9	Quer-3-DHA (**15**)	134.10 ± 8.09 *; °°°	59.42 ± 18.80 ***
10	Quer-3-ALA (**14**)	69.44 ± 3.46 ***	61.72 ± 7.43 ***
11	Quer-7-ALA (**18**)	>160	9.44 ± 2.37 **; ###

Results are expressed in concentrations (µM) ± SD and are mean of at least n = 3 independent experiments. ^1^ Concentration of lipophenol killing 50% of ARPE-19 cells. ^2^ Concentration of lipophenol needed to inhibit 50% of the ROS induced by H_2_O_2_. Student’s *t*-test was applied for CC_50_ and Mann-Whitney’s test was applied for ROS production IC_50_. * *p* < 0.05, ** *p* < 0.01, *** *p* < 0.001, versus natural quercetin (entry 7). ° *p* < 0.05, °° *p* < 0.01, °°° *p* < 0.001, versus quer-3-ALA (entry 10). ◆ *p* < 0.05, ◆◆ *p* < 0.01, ◆◆◆ *p* < 0.001, versus (+)-catechin (entry 5). # *p* < 0.05, ## *p* < 0.01, ### *p* < 0.001, versus quer-3-ALA (entry 10).

## Supplementary Materials

The following are available online at http://www.mdpi.com/2076-3921/7/12/197/s1, Figure S1: Graphical representation of in vitro evaluations of lipophenols (toxicity and ROS inhibition), Experimental description of lipophenols synthesis and full NMR spectra characterization of derivatives from 2 to 18 (^1^H and ^13^C, Figure S2).
