# Supplementary material for: New Lipophenol Antioxidants Reduce Oxidative Damage in Retina Pigment Epithelial Cells
Source: Antioxidants (Basel). 2018 Dec 19;7(12):197. doi: 10.3390/antiox7120197 (PMC6315395; doi:10.3390/antiox7120197)

New lipophenol antioxidants reduce oxidative damage in retina pigment epithelial cells.

Espérance Moine [1], Philippe Brabet [2], Laurent Guillou [2], Thierry Durand [1], Joseph Vercauteren [1] and Céline Crauste [1,*]

[1] Institute of Biomolecules Max Mousseron (IBMM), UMR 5247-CNRS-UM-ENSCM, Faculty of Pharmacy, 15 av. Charles Flahault, 34093 Montpellier, France, celine.crauste@umontpellier.fr
[2] Institute for Neurosciences of Montpellier, INSERM U1051-UM, Hospital St Eloi, 80 rue Augustin Fliche, 34091 Montpellier, France, philippe.brabet@inserm.fr
* Correspondence: celine.crauste@umontpellier.fr; Tel.: +33-4-11-75-95-66

Supplementary materials

Experimental procedures for lipophenols synthesis.

Figure S1: Graphical representation of *in vitro* evaluations of lipophenols.

Figure S2: Full NMR spectra characterization of derivatives from 2 to 18 (^{1}H and ^{13}C).

Experimental procedures for lipophenols synthesis.

3,5-bis(triisopropylsilyloxy)phenol (1): was synthesized according to the procedure described by Crauste et *al.* 2014 [26].

(9,12*Z*)-3,5-bis(triisopropylsilyloxy)phenyl-octadeca-9,12-dienoate (2): To a solution of linoleic acid (77 mg, 0.27 mmol), dicyclohexylcarbodiimide (62 mg, 0.30 mmol) and 4-dimethylaminopyridine (10 mg, 0.08 mmol) in anhydrous CH_2Cl_2 (2 mL) stirred under N_2, was added a solution of compound **1** (120 mg, 0.27 mmol) in anhydrous CH_2Cl_2 (2 mL). The resulting mixture was stirred at room temperature for 2 h and cooled to 4 °C for 1 h to afford filtration of the precipitate. The filtrate was washed with water and brine, dried over $MgSO_4$, filtered and evaporated under reduced pressure. Purification of the residue was performed by silica column chromatography using hexane/EtOAc (99:1 v/v) as eluent to give compound **2** (120 mg, 62%) as colorless oil. R_f (hexane/EtOAc, 99:1) = 0.26. ^{1}H NMR (500 MHz, $CDCl_3$) δ 6.28 (t, *J* = 1.9 Hz, 1H, H4), 6.24 (d, *J* = 1.9 Hz, 2H, H2, H6), 5.44 – 5.27 (m, 4H, 2 × CH=CH), 2.78 (t, *J* = 6.6 Hz, 2H, H11′), 2.50 (t, *J* = 7.5 Hz, 2H, H2′), 2.12 – 1.99 (m, 4H, H8′, H14′), 1.66 (qt, *J* = 7.5 Hz, 2H, H3′), 1.43 – 1.17 (m, 20H, H4′, H5′, H6′, H7′, H15′, H16′, H17′, 6 × CHTIPS), 1.10 – 0.95 (m, 36H, 12 × CH_3TIPS), 0.89 (t, *J* = 6.8 Hz, 3H, H18′). ^{13}C NMR (125 MHz, $CDCl_3$) δ 171.9, 157.1 (2C), 151.8, 130.2, 130.0, 128.1, 127.9, 109.3, 106.9, 34.4, 31.5, 29.6 (2C), 29.4 (2C), 29.2, 29.2, 29.1, 27.2, 25.6, 24.9, 22.6, 17.9 (12C), 14.1, 12.6 (6C). HRMS (ESI+I): calculated for $C_{42}H_{77}O_4Si_2$ $[M + H]^+$ 701.5355; found 701.5366.

(9Z,12Z)-3,5-dihydroxyphenyl octadeca-9,12-dienoate (phloro-LA, 3): To a solution of compound **2** (59 mg, 0.08 mmol) in anhydrous THF (4 mL) stirred under N_2, was added dropwise triethylamine trihydrofluoride (41 μL, 0.24 mmol). The resulting mixture was stirred at room temperature for 9 h, diluted in EtOAc and washed with water and brine. The organic layer was dried over $MgSO_4$, filtered and evaporated under reduced pressure. Purification of the residue was performed by silica column chromatography using hexane/EtOAc (80:20 v/v) as eluent to give compound **3** (30 mg, 92%) as colorless oil. R_f (hexane/EtOAc, 70:30) = 0.30. ^{1}H NMR (500 MHz, $CDCl_3$) δ 6.06 (s, 3H, H2, H4, H6), 5.45 – 5.27 (m, 4H, 2 × CH=CH), 2.78 (t, *J* = 6.8 Hz, 2H, H11′), 2.57 (t, *J* = 7.5 Hz, 2H, H2′), 2.10 – 2.00 (m, 4H, H8′, H14′), 1.75 (qt, *J* = 7.5 Hz, 2H, H3′), 1.46 – 1.22 (m, 14H, H4′, H5′, H6′, H7′, H15′, H16′, H17′), 0.89 (t, *J* = 6.8 Hz, 3H, H18′). ^{13}C NMR (125 MHz, $CDCl_3$) δ 174.6, 157.4 (2C), 151.7, 130.3, 130.0, 128.1, 127.9, 102.0, 101.5, 34.4, 31.5, 29.6 (2C), 29.4 (2C), 29.2, 29.1, 29.1, 27.2, 25.7, 24.9, 22.6, 14.1. HRMS (ESI-I): calculated for $C_{24}H_{35}O_4$ $[M - H]^-$ 387.2535; found 387.2534.

(*E*)-4-(3,5-dihydroxystyryl)phenyl acetate (4): resveratrol (2,88 g, 12.61 mmol) was dissolved in 2-methylbutan-2-ol (280 mL) and vinyl acetate (72.40 mL, 756.70 mmol) in presence of the supported lipase *Candida Antarctica* (Novozyme 435, CalB, 14.40 g). The mixture was stirred with a rotary evaporator at 40°C during 4 days, protected from sunlight by aluminium foil. The lipase was then filtered off and washed with EtOAc and diethyl ether. The filtrate obtained was concentrated under reduced pressure and the residue obtained was purified by chromatography on silica gel using solid deposit using CH_2Cl_2/MeOH (99/1 to 98/2 v/v) as eluent to give the 4′-*O*-acetyl resveratrol **4** (2.89 mg, 85%) as white solid. R_f (CH_2Cl_2/MeOH, 95:5) = 0.3. Mp 199.1 °C. ^{1}H NMR (500 MHz, MeOD) δ 7.54 (d, *J* = 7.6 Hz, 2H, H2′, H6′), 7.07 (d, *J* = 7.6 Hz, 2H, H3′, H5′), 7.04 (d, *J* = 16.2 Hz, 1H, H8), 6.97 (d, *J* = 16.2 Hz, 1H, H7), 6.49 (s, 2H, H2, H6), 6.21-6.19 (m, 1H, H4), 2.27 (s, 3H, CH_3OCO). ^{13}C NMR (125 MHz, MeOD) δ 171.1, 159.7 (2C), 151.5, 140.6, 136.6, 130.2, 128.3, 128.3, 122.9 (2C), 122.9, 106.1 (2C), 103.2, 20.9. HRMS (ESI+I): calculated for $C_{16}H_{15}O_4$ $[M + H]^+$ 271.0964; found 271.0972.

(*E*)-4-(3,5-bis((triisopropylsilyl)oxy)styryl)phenyl acetate (5): 4′-*O*-acetyl resveratrol **4** (3.18 g, 11.78 mmol) was dissolved in dry THF (160 mL). DIPEA (4.20 mL, 24.70 mmol) and TIPS-OTf (6.70 mL, 24.70 mmol) were added dropwise to the solution and the reaction mixture was stirred at room temperature during 4.5h. Additional amount of DIPEA (1.0 mL, 5.90 mmol) and TIPS-OTf (1.6 mL,

5.90 mmol) were added to reach completion of the reaction. After 2.5 additional hours of reaction, the solvent was evaporated under reduced pressure. The residue obtained was dissolved in EtOAc and washed with water (x2) and brine. The organic phase was dried over $MgSO_4$ and concentrated under vacuum. The residue obtained was purified by chromatography on silica gel using pentane/EtOAc (99:1 to 70:30 v/v) as eluent to give the protected resveratrol **5** (6.85 g, 90%) as a colorless oil. R_f (pentane/EtOAc, 95:5) = 0.5. ^{1}H NMR (500 MHz, $CDCl_3$) δ 7.51 (d, *J* = 8.0 Hz, 2H, H2′, H6′), 7.09 (d, *J* = 8.0 Hz, 2H, H3′, H5′), 6.98 (d, *J* = 16.3 Hz, 1H, H8), 6.92 (d, *J* = 16.3 Hz, 1H, H7), 6.65 (s, 2H, H2, H6), 6.37 – 6.36 (m, 1H, H4), 2.31 (s, 3H, CH_3OCO), 1.26 (m, 6H, 6 × CHTIPS), 1.12 (d, *J* = 7.6 Hz, 36H, 12 × CH_3TIPS). ^{13}C NMR (125 MHz, $CDCl_3$) δ 169.7, 157.3 (2C), 150.2, 139.0, 135.3, 129.3, 127.8, 127.7 (2C), 122.0 (2C), 111.6 (2C), 111.5, 21.4, 18.2 (6C), 12.9 (12C). HRMS (ESI+I): calculated for $C_{34}H_{55}O_4Si_2$ $[M + H]^+$ 583.3633; found 583.3640.

(*E*)-4-(3,5-bis((triisopropylsilyl)oxy)styryl)phenol (6): The protected resveratrol **5** (6.85 g, 10.59 mmol) was dissolved in dry MeOH (58 mL) and CH_2Cl_2 (28 mL). Sodium methoxide (191 mg, 3.53 mmol) was added to the solution and the reaction mixture was stirred at room temperature during 4.5h. Further, 0.3 eq of NaOMe (191 mg, 3.53 mmol) was added to drive the reaction to completion. After additional 2h, the solvent was evaporated under reduced pressure. The residue obtained was purified by chromatography on silica gel using pentane/EtOAc (96:4 to 90:10 v/v) as eluent to give the 4′-deprotected resveratrol **6** (6.04 g, 95%) as a colorless oil. R_f (hexane/EtOAc, 90:10) = 0.41. ^{1}H NMR (500 MHz; MeOD) δ 7.38 (d, *J* = 8.5 Hz, 2H, H2′, H6′), 6.95 (d, *J* = 16.2 Hz, 1H, H8), 6.84 (d, *J* = 16.2 Hz, 1H, H7), 6.77 (d, *J* = 8.5 Hz, 2H, H3′, H5′), 6.64 – 6.63 (m, 2H, H2, H6), 6.30 – 6.29 (m, 1H, H4), 1.30 – 1.22 (m, 6H, 6 × CHTIPS), 1.14 (d, *J* = 7.5 Hz, 36H, 12 × CH_3TIPS). ^{13}C NMR (125 MHz; MeOD) δ 158.5, 158.3 (2C), 141.3, 130.1, 129.9, 129.0 (2C), 126.5, 116.5 (2C), 112.1 (2C), 111.4, 18.4 (6C), 13.9 (12C). HRMS (ESI+I): calculated for $C_{32}H_{53}O_3Si_2$ $[M + H]^+$ 541.3527; found 541.3536.

(9,12*Z*)-4-((*E*)-3,5-bis((triisopropylsilyl)oxy)styryl)phenyl octadeca-9,12-dienoate (7): Compound **6** (1.00 g, 1.67 mmol) and linoleic acid (623 mg, 2,22 mmol) were dissolved in anhydrous CH_2Cl_2 (40 mL). Dicyclohexylcarbodiimide (573 mg, 2.78 mmol) and 4-dimethylaminopyridine (113 mg, 0.93 mmol) were added to the reaction and the resulting mixture was stirred at room temperature under under N_2 for 3 h before cooling to 4 °C for 1 h to afford filtration of the precipitate. The filtrate was washed with water and brine, dried over $MgSO_4$, filtered and evaporated under reduced pressure. Purification of the residue was performed by silica column chromatography using pentane/EtOAc (99.5:0.5 to 99:1 v/v) as eluent to give compound **6** as a colorless oil (1.10 g, 74%). *Rf* (pentane/EtOAc, 95:5) = 0.7. ^{1}H NMR (500 MHz, $CDCl_3$) δ 7.79 (d, *J* = 8.5 Hz, 2H, H2′, H6′), 7.06 (d, *J* = 8.5 Hz, 2H, H3′, H5′), 6.97 (d, *J* = 16 Hz, 1H, H8), 6.91 (d, *J* = 16 Hz, 1H, H7), 6.64 (d, *J* =1.5 Hz, 2H, H2, H6), 6.3 (t, *J* = 1.5 Hz, 1H, H4), 5.41 – 5.33 (m, 4H, 2 × CH=CH), 2.78 (t, *J* = 6.5 Hz, 2H, H11′′), 2.55 (t, *J* = 7.5 Hz, 2H, H2′′), 2.07 – 2.03 (m, 4H, H8′′, H14′′), 1.75 (qt, *J* = 7.5 Hz, 2H, H3′′), 1.42 – 1.22 (m, 20H, H4′′, H5′′, H6′′, H7′′, H15′′, H16′′, H17′′, 6 × CHTIPS), 1.11 (d, *J* = 7.5 Hz, 36 H, 12 × CH_3TIPS), 0.89 (t, *J* = 7 Hz, 3H, H18′′). ^{13}C NMR (125 MHz, $CDCl_3$) δ 172.6, 157.4 (2C), 150.4, 139.1, 135.3, 130.6, 130.3, 129.3, 128.4, 128.2, 127.9, 127.7 (2C), 122.1 (2C), 111.7 (2C), 111.6, 34.8, 31.8, 29.9, 29.7, 29.5, 29.4, 29.4, 27.5, 27.5, 26.0, 25.3, 22.9, 18.2 (6C), 14.4, 13.0 (12C). HRMS (ASAP+I): calculated for $C_{50}H_{83}O_4Si_2$ $[M + H]^+$ 803.5830; found 803.5815.

(9,12*Z*)-4-((*E*)-3,5-dihydroxystyryl)phenyl octadeca-9,12-dienoate (Resv-4′-LA, 8): To a solution of compound **7** (1.08 g, 1.35 mmol) in anhydrous THF (60 mL) stirred under N_2, was added dropwise triethylamine trihydrofluoride (1.32 mL, 8.08 mmol). The reaction was stirred at room temperature and further equivalents of triethylamine trihydrofluoride (2×0.66 mL, 2×4.04 mmol) were added at four and six hours of reaction time. Reaction was terminated after another two hours. The resulting mixture was evaporated and the residue was dissolved in EtOAc. Organic phases were washed with water and brine, dried over $MgSO_4$, filtered and evaporated under reduced pressure. Crude product was purified by column chromatography on silica gel using pentane/EtOAc (70:30 to 60:40 v/v) as eluent to obtain the desired compound **8** (546 mg, 83%) as a white solid. R_f (pentane/EtOAc, 70:30) =

0.3. ^{1}H NMR (500 MHz, $CDCl_3$) δ 7.34 (d, *J* = 8.5 Hz, 2H, H2′, H6′), 7.00 (d, *J* = 8.5 Hz, 2H, H3′, H5′), 6.79 (d, *J* = 16.5 Hz, 1H, H8), 6.70 (d, *J* = 16.5 Hz, 1H, H7), 6.40 (d, *J* = 2.0 Hz, 2H, H2, H6), 6.23 (t, *J* = 2.0 Hz, 1H, H4), 5.99 (bs, 2H, 2 × OH), 5.41 – 5.32 (m, 4H, 2 × CH=CH), 2.77 (t, *J* = 6.5 Hz, 2H, H11′′), 2.56 (t, *J* = 7.5 Hz, 2H, H2′′), 2.06 – 2.03 (m, 4H, H8′′, H14′′), 1.75 (qt, *J* = 7.5 Hz, 2H, H3′′), 1.42 – 1.25 (m, 14H, H4′′, H5′′, H6′′, H7′′, H15′′, H16′′, H17′′), 0.88 (t, *J* = 7 Hz, 3H, H18′′). ^{13}C NMR (125 MHz, $CDCl_3$) δ 173.5, 157.0 (2C), 150.1, 139.7, 135.1, 130.4, 130.2, 128.4, 128.2, 128.2, 128.0, 127.7 (2C), 121.8 (2C), 106.3 (2C), 102.6, 34.6, 31.7, 29.7, 29.5, 29.3, 29.3, 29.2, 27.3, 27.3, 25.7, 25.0, 22.7, 14.2. HRMS (ASAP+I): calculated for $C_{32}H_{43}O_4$ $[M + H]^+$ 491.3161; found 491.3161.

(9Z,12*Z*)-(2*R*,3*S*)-2-(3,4-dihydroxyphenyl)-5,7-dihydroxy-chroman-3-yl octadeca-9,12-dienoate (Cat-3-LA, 9): To a suspension of freshly prepared linoleyl chloride (103 mg, 0.34 mmol) in anhydrous CH_2Cl_2 (5 mL) stirred under N_2, was added trifluoroacetic acid (26 µL, 0.34 mmol) and dropwise a suspension of dried (+)-catechin (100 mg, 0.34 mmol) in anhydrous DMF (2 mL). The resulting mixture was stirred at room temperature for 22 h and at 40 °C for 6 h, diluted in EtOAc/MeOH (3:1 v/v) and evaporated under reduced pressure. The crude product was dissolved in EtOAc, washed with water, dried over $MgSO_4$, filtered and evaporated under reduced pressure. Purification of the residue was performed by silica column chromatography using CH_2Cl_2/MeOH (98:2 to 96:4 v/v) as eluent to give compound **9** (4.9 mg, 2.6%) as colorless oil. R_f (CH_2Cl_2/MeOH, 80:20) = 0.80. ^{1}H NMR (500 MHz, MeOD) δ 6.80 (d, *J* = 2.0 Hz, 1H, H2'), 6.73 (d, *J* = 8.1 Hz, 1H, H5'), 6.68 (dd, *J* = 8.1, 2.0 Hz, 1H, H6'), 5.95 (d, *J* = 2.2 Hz, 1H, H8), 5.89 (d, *J* = 2.2 Hz, 1H, H6), 5.42 – 5.27 (m, 4H, 2 × CH=CH), 5.20 (dd, *J* = 12.4, 7.0 Hz, 1H, H3), 4.85 (d, *J* = 7.0 Hz, 1H, H2), 2.84 – 2.74 (m, 3H, H4β, H11''), 2.60 (dd, *J* = 16.3, 7.0 Hz, 1H, H4α), 2.20 (td, *J* = 7.3, 1.8 Hz, 2H, H2''), 2.06 (q, *J* = 6.9 Hz, 4H, H8'', H14''), 1.50 – 1.40 (m, 2H, H3''), 1.40 – 1.11 (m, 14H, H4'', H5'', H6'', H7'', H15'', H16'', H17''), 0.90 (t, *J* = 6.9 Hz, 3H, H18''). ^{13}C NMR (125 MHz, MeOD) δ 174.61, 158.17, 157.61, 156.60, 146.45, 146.31, 131.15, 130.98, 130.93, 129.09, 129.04, 119.53, 116.10, 114.79, 99.72, 96.49, 95.56, 79.68, 70.97, 35.22, 32.65, 30.68, 30.47, 30.17, 30.10, 29.90, 28.16, 28.14, 26.54, 26.00, 25.13, 23.61, 14.41. HRMS (ESI-I): calculated for $C_{36}H_{49}O_7$ $[M-H]^-$ 593.3478; found 593.3477.

(9Z,12*Z*)-2-(3,4-((9Z,12*Z*)-dioctadeca-9,12-dienoyloxy)phenyl)-4-oxo-4*H*-chromen-3,5,7-triyl trioctadeca-9,12-dienoate (10): To a solution of dried quercetin (0.1 g, 0.33 mmol) in 1,4-dioxan (8 mL) stirred under N_2, was added distilled triethylamine (0.35 mL, 2.48 mmol) and a suspension of freshly prepared linoleyl chloride (0.74 g, 2.48 mmol) in 1,4-dioxan (2 mL). The resulting mixture was stirred in the dark at room temperature for 19 h and evaporated under reduced pressure. The crude product was dissolved in CH_2Cl_2, washed with aqueous saturated $NaHCO_3$ solution and water, dried over $MgSO_4$, filtered and evaporated under reduced pressure. Purification of the residue was performed by silica column chromatography using CH_2Cl_2/MeOH (100:0 to 99:1 v/v) as eluent to give compound **10** (236 mg, 75%) as colorless oil. R_f (CH_2Cl_2/MeOH, 99:1) = 0.67. ^{1}H NMR (500 MHz, $CDCl_3$) δ 7.70 (dd, *J* = 8.6, 2.1 Hz, 1H, H6'), 7.66 (d, *J* = 2.1 Hz, 1H, H2'), 7.32 (d, *J* = 8.6 Hz, 1H, H5'), 7.31 (d, *J* = 2.1 Hz, 1H, H8), 6.84 (d, *J* = 2.1 Hz, 1H, H6), 5.47 – 5.25 (m, 20H, 10 × CH=CH), 2.81 – 2.75 (m, 10H, 5 × H11''), 2.73 (t, *J* = 7.6 Hz, 2H, H2''), 2.62 – 2.53 (m, 8H, 4 × H2''), 2.10 – 2.01 (m, 20H, 5 × H8'', 5 × H14''), 1.85 – 1.65 (m, 10H, 5 × H3''), 1.47 – 1.22 (m, 70H, 5 × H4'', 5 × H5'', 5 × H6'', 5 × H7'', 5 × H15'', 5 × H16'', 5 × H17''), 0.92 – 0.85 (m, 15H, 5 × H18''). ^{13}C NMR (125 MHz, $CDCl_3$) δ 172.01, 170.77, 170.62, 170.58, 170.53, 169.97, 156.84, 154.27, 153.60, 150.53, 144.45, 142.27, 134.07, 130.23, 130.22, 130.21, 130.19, 130.18, 130.07, 130.03, 129.94, 129.89, 129.88, 128.11, 128.10, 128.08, 127.98, 127.97, 127.90, 127.88, 127.85, 127.82 (2C), 127.75, 126.37, 123.83, 123.77, 114.79, 113.86, 108.85, 34.35, 34.09, 34.05, 33.99, 33.73, 31.49 (5C), 29.63 (2C), 29.61, 29.59 (2C), 29.56, 29.32 (4C), 29.31, 29.24, 29.23, 29.19, 29.16, 29.15, 29.12, 29.11 (2C), 29.10, 29.09, 29.08, 29.04, 28.98, 28.97, 27.21, 27.20, 27.17 (3C), 27.16 (3C), 27.15, 27.14, 25.62 (5C), 24.86, 24.83, 24.66, 24.56, 24.41, 22.56 (5C), 14.07 (5C).

(9Z,12*Z*,15*Z*)-2-(3,4-((9*Z*,12*Z*,15*Z*)-dioctadeca-9,12,15-trienoyloxy)phenyl)-4-oxo-4*H*-chromen-3,5,7-triyl trioctadeca-9,12,15-trienoate (11): To a solution of α-linolenic acid (ALA, 244 mg, 0.88 mmol), dicyclohexylcarbodiimide (181 mg, 0.88 mmol) and 4-dimethylaminopyridine (10 mg, 0.08 mmol) in

anhydrous CH_2Cl_2 (7 mL) stirred under N_2, was added a suspension of dried quercetin (50 mg, 0.17 mmol) in anhydrous CH_2Cl_2 (8 mL). The resulting mixture was stirred at room temperature for 4.5 h and cooled to 4 °C for 2 h to afford filtration of the precipitate. The filtrate was washed with water and brine, dried over $MgSO_4$, filtered and evaporated under reduced pressure. Purification of the residue was performed by silica column chromatography using CH_2Cl_2 as eluent to give compound **11** (84 mg, 32%) as yellow oil. R_f (CH_2Cl_2/MeOH, 99:1) = 0.87. ^{1}H NMR (500 MHz, $CDCl_3$) δ 7.73 (dd, *J* = 8.5, 2.1 Hz, 1H, H6'), 7.70 (d, *J* = 2.1 Hz, 1H, H2'), 7.34 (d, *J* = 8.5 Hz, 1H, H5'), 6.84 (d, *J* = 2.0 Hz, 1H, H8), 6.58 (d, *J* = 2.0 Hz, 1H, H6), 5.47 – 5.23 (m, 30H, 15 × CH=CH), 2.85 – 2.77 (m, 20H, 5 × H11'', 5 × H14''), 2.63 (t, *J* = 7.5 Hz, 2H, H2''), 2.60 – 2.54 (m, 8H, 4 × H2''), 2.13 – 2.02 (m, 20H, 5 × H8'', 5 × H17''), 1.81 – 1.69 (m, 10H, 5 × H3''), 1.48 – 1.28 (m, 40H, 5 × H4'', 5 × H5'', 5 × H6'', 5 × H7''), 1.02 – 0.92 (m, 15H, 5 × H18''). ^{13}C NMR (125 MHz, $CDCl_3$) δ 176.26, 171.08, 170.64 (2C), 170.60, 170.51, 161.67, 156.41, 155.91, 155.47, 144.71, 142.30, 132.19, 131.93 (2C), 131.92 (2C), 130.24 (2C), 130.17 (2C), 130.09 (2C), 130.08 (2C), 128.29 (2C), 128.25 (2C), 128.24 (2C), 128.21 (2C), 128.19 (2C), 128.15 (2C), 127.80 (2C), 127.75 (2C), 127.69, 127.47, 127.07, 126.46, 123.95, 123.93, 108.71, 105.44, 101.12, 34.37, 34.05 (2C), 34.00, 33.70, 29.60 (2C), 29.55 (2C), 29.23 (2C), 29.20 (2C), 29.12 (2C), 29.10 (2C), 29.08 (2C), 29.05 (2C), 29.00 (2C), 28.97 (2C), 27.20 (2C), 27.18 (3C), 25.59 (5C), 25.49 (5C), 24.84 (2C), 24.83, 24.73, 24.63, 20.52 (5C), 14.26 (5C).

(4Z,7Z,10Z,13Z,16Z,19*Z*)-2-(3,4-((4Z,7Z,10Z,13Z,16Z,19*Z*)-didocosa-4,7,10,13,16,19-hexaenoyloxy)phenyl)-4-oxo-4*H*-chromen-3,5,7-triyl tridocosa-4,7,10,13,16,19-hexaenoate (12): To a solution of *cis*-4,7,10,13,16,19-docosahexaenoic acid (300 mg, 0.91 mmol), dicyclohexylcarbodiimide (188 mg, 0.91 mmol) and 4-dimethylaminopyridine (11 mg, 0.09 mmol) in anhydrous CH_2Cl_2 (7 mL) stirred under N_2, was added a suspension of dried quercetin (52 mg, 0.17 mmol) in anhydrous CH_2Cl_2 (8 mL). The resulting mixture was stirred at room temperature for 19 h and cooled to 4 °C for 2 h to afford filtration of the precipitate. The filtrate was washed with water and brine, dried over $MgSO_4$, filtered and evaporated under reduced pressure. Purification of the residue was performed by silica column chromatography using CH_2Cl_2 as eluent to give compound **12** (98 mg, 31%) as yellow oil. R_f (CH_2Cl_2) = 0.78. ^{1}H NMR (300 MHz, $CDCl_3$) δ 7.76 – 7.70 (m, 2H, H2', H6'), 7.35 (dd, *J* = 8.0, 0.9 Hz, 1H, H5'), 6.85 (d, *J* = 2.0 Hz, 1H, H8), 6.59 (d, *J* = 2.0 Hz, 1H, H6), 5.58 – 5.23 (m, 60H, 30 × CH=CH), 2.93 – 2.76 (m, 50H, 5 × H6'', 5 × H9'', 5 × H12'', 5 × H15'', 5 × H18''), 2.76 – 2.60 (m, 10H, 5 × H2''), 2.59 – 2.46 (m, 10H, 5 × H3''), 2.07 (qt, *J* = 7.5 Hz, 10H, 5 × H21''), 0.97 (t, *J* = 7.5 Hz, 15H, 5 × H22''). ^{13}C NMR (75 MHz, $CDCl_3$) δ 176.15, 170.36, 170.00 (2C), 169.95, 169.85, 161.69, 156.35, 155.89, 155.45, 144.64, 142.23, 132.21, 132.00 (5C), 130.05 (5C), 129.75 (5C), 128.50 (5C), 128.42 (5C), 128.28 (5C), 127.99 (5C), 127.81 (5C), 127.74 (5C), 127.48, 127.32 (5C), 127.09 (5C), 126.97 (5C), 126.54, 123.94 (2C), 108.74, 105.48, 101.11, 34.26, 33.91 (3C), 33.56, 25.60 (15C), 25.50 (10C), 22.52 (5C), 20.52 (5C), 14.25 (5C).

(9Z,12*Z*)-2-(3,4-dihydroxyphenyl)-5,7-dihydroxy-4-oxo-4*H*-chromen-3-yl octadeca-9,12-dienoate (Quer-3-LA, 13): To a solution of compound **10** (106 mg, 0.07 mmol) in *tert*-butylmethylether (7.5 mL) was added *n*-BuOH (0.3 mL) and supported lipozyme® from *Mucor miehei* (1:1 w/w, 106 mg). The resulting mixture was stirred at 45 °C for 6 days, supported enzyme was filtrated and rinsed several times with EtOAc and Et_2O. The filtrate was evaporated under reduced pressure. Purification of the residue was performed by silica column chromatography using CH_2Cl_2/MeOH (95:5 v/v) as eluent to give compound **13** (20 mg, 54%) as yellow solid. R_f (pentane/EtOAc, 50:50) = 0.25. Mp 193.8 °C. ^{1}H NMR (500 MHz, MeOD) δ 7.33 (d, *J* = 2.2 Hz, 1H, H2'), 7.28 (dd, *J* = 8.4, 2.2 Hz, 1H, H6'), 6.89 (d, *J* = 8.4 Hz, 1H, H5'), 6.43 (d, *J* = 2.1 Hz, 1H, H8), 6.24 (d, *J* = 2.1 Hz, 1H, H6), 5.41 – 5.27 (m, 4H, 2 × CH=CH), 2.77 (t, *J* = 6.5 Hz, 2H, H11''), 2.63 (t, *J* = 7.3 Hz, 2H, H2''), 2.12 – 2.00 (m, 4H, H8'', H14''), 1.70 (qt, *J* = 7.3 Hz, 2H, H3''), 1.43 – 1.24 (m, 14H, H4'', H5'', H6'', H7'', H15'', H16'', H17''), 0.89 (t, *J* = 6.8 Hz, 3H, H18''). ^{13}C NMR (125 MHz, MeOD) δ 177.22, 172.55, 166.30, 163.13, 158.68, 158.45, 150.37, 146.64, 131.43, 130.91, 130.90, 129.07, 129.06, 122.13, 121.97, 116.45, 116.18, 105.27, 100.14, 95.03, 34.61, 32.65, 30.63, 30.48, 30.19, 30.13, 29.98, 28.15, 28.12, 26.53, 25.80, 23.63, 14.42. HRMS (ESI-I): calculated for $C_{33}H_{39}O_8$ $[M-H]^-$ 563.2645; found 563.2654.

(9Z,12Z,15Z)-2-(3,4-dihydroxyphenyl)-5,7-dihydroxy-4-oxo-4*H*-chromen-3-yl octadeca-9,12,15-trienoate (Quer-3-ALA, 14): To a solution of compound **11** (46 mg, 29 µmol) in *tert*-butylmethylether (3.11 mL) was added *n*-BuOH (0.13 mL) and supported lipozyme® from *Mucor miehei* (1:1 w/w, 46 mg). The resulting mixture was stirred at 45 °C for 8 days, supported enzyme was filtrated and rinsed several times with EtOAc and Et_2O. The filtrate was evaporated under reduced pressure. Purification of the residue was performed by silica column chromatography using CH_2Cl_2/MeOH (95:5 v/v) as eluent to give compound **14** (16 mg, 96%) as yellow solid. R_f (CH_2Cl_2/MeOH, 95:5) = 0.25. Mp 180.0 °C. ^{1}H NMR (500 MHz, MeOD) δ 7.33 (d, *J* = 2.1 Hz, 1H, H2'), 7.28 (dd, *J* = 8.4, 2.1 Hz, 1H, H6'), 6.89 (d, *J* = 8.4 Hz, 1H, H5'), 6.43 (d, *J* = 1.9 Hz, 1H, H8), 6.23 (d, *J* = 1.9 Hz, 1H, H6), 5.42 – 5.18 (m, 6H, 3 × CH=CH), 2.85 – 2.75 (m, 4H, H11'', H14''), 2.63 (t, *J* = 7.1 Hz, 2H, H2''), 2.12 – 2.02 (m, 4H, H8'', H17''), 1.70 (qt, *J* = 7.1 Hz, 2H, H3''), 1.41 – 1.26 (m, 8H, H4'', H5'', H6'', H7''), 0.95 (t, *J* = 7.5 Hz, 3H, H18''). ^{13}C NMR (125 MHz, MeOD) δ 177.21, 172.56, 166.26, 163.12, 158.67, 158.42, 150.35, 146.63, 132.71, 131.45, 131.13, 129.23, 129.17, 128.82, 128.23, 122.16, 121.99, 116.46, 116.20, 105.30, 100.14, 95.05, 34.63, 30.63, 30.20, 30.14, 30.00, 28.14, 26.52, 26.40, 25.81, 21.48, 14.66. HRMS (ESI-I): calculated for $C_{33}H_{37}O_8$ [M-H]$^-$ 561.2488; found 561.2491.

(4Z,7Z,10Z,13Z,16Z,19Z)-2-(3,4-dihydroxyphenyl)-5,7-dihydroxy-4-oxo-4*H*-chromen-3-yl docosa-4,7,10,13,16,19-hexaenoate (Quer-3-DHA, 15): To a solution of compound **12** (39 mg, 21 µmol) in *tert*-butylmethylether (2.7 mL) was added *n*-BuOH (0.1 mL) and supported lipozyme® from *Mucor miehei* (1:1 w/w, 39 mg). The resulting mixture was stirred at 45 °C for 8 days, supported enzyme was filtrated and rinsed several times with EtOAc and Et_2O. The filtrate was evaporated under reduced pressure. Purification of the residue was performed by silica column chromatography using CH_2Cl_2/MeOH (97:3 v/v) as eluent to give compound **15** (8 mg, 62%) as sticky yellow solid. R_f (CH_2Cl_2/MeOH, 95:5) = 0.20. Mp 191.3 °C. ^{1}H NMR (300 MHz, MeOD) δ 7.34 (d, *J* = 2.2 Hz, 1H, H2'), 7.28 (dd, *J* = 8.4, 2.2 Hz, 1H, H6'), 6.89 (d, *J* = 8.4 Hz, 1H, H5'), 6.43 (d, *J* = 2.1 Hz, 1H, H8), 6.24 (d, *J* = 2.1 Hz, 1H, H6), 5.51 – 5.16 (m, 12H, 6 × CH=CH), 2.89 – 2.74 (m, 10H, H6'', H9'', H12'', H15'', H18''), 2.70 (t, *J* = 7.3 Hz, 2H, H2''), 2.48 (q, *J* = 7.3 Hz, 2H, H3''), 2.04 (qt, *J* = 7.5 Hz, 2H, H21''), 0.94 (t, *J* = 7.5 Hz, 3H, H22''). ^{13}C NMR (75 MHz, MeOD) δ 177.15, 171.95, 166.27, 163.13, 158.67, 158.38, 150.38, 146.67, 132.73, 131.46, 130.61 (2C), 129.38 (3C), 129.12 (2C), 129.06, 128.89, 128.68, 128.18, 122.19, 121.96, 116.47, 116.20, 105.30, 100.14, 95.04, 34.68, 26.55 (4C), 26.39, 23.67, 21.46, 14.65. HRMS (ESI-I): calculated for $C_{37}H_{39}O_8$ [M-H]$^-$ 611.2645; found 611.2651.

3-(*tert*-butyldimethylsilyloxy)-2-(3,4-bis-(*tert*-butyldimethylsilyloxy)phenyl)-5,7-dihydroxy-4*H*-chromen-4-one (16): To a solution of dried quercetin (0.5 g, 1.65 mmol) in anhydrous CH_2Cl_2 (5 mL) stirred under N_2, was added distilled DBU (0.87 mL, 5.79 mmol) and TBDMSCl (0.77 g, 5.13 mmol). The resulting mixture was stirred at room temperature for 45 min, diluted in CH_2Cl_2 and washed several times with water. The organic layer was dried over $MgSO_4$, filtered and evaporated under reduced pressure. Purification of the residue was performed by silica column chromatography using CH_2Cl_2 as eluent to give compound **16** (240 mg, 22%) as yellow oil, isolated among penta- and tetra-derivatives. R_f (CH_2Cl_2/MeOH, 99:1) = 0.37. ^{1}H NMR (500 MHz, $CDCl_3$) δ 7.45 (dd, J = 8.5, 2.5 Hz, 1H, H6'), 7.33 (d, J = 2.5 Hz, 1H, H2'), 6.89 (d, *J* = 8.5 Hz, 1H, H5'), 6.35 (d, *J* = 2.0 Hz, 1H, H8), 6.26 (d, *J* = 2.0 Hz, 1H, H6), 1.81 (bs, 1H, OH), 1.00 (s, 9H, 3 × CH_3*t*Bu), 0.99 (s, 9H, 3 × CH_3*t*Bu), 0.83 (s, 9H, 3 × CH_3*t*Bu), 0.23 (s, 6H, 2 × CH_3Si), 0.20 (s, 6H, 2 × CH_3Si), 0.11 (s, 6H, 2 × CH_3Si). ^{13}C NMR (125 MHz, $CDCl_3$) δ 178.18, 162.03, 161.64, 156.68, 153.39, 149.24, 146.74, 135.49, 124.26, 123.28, 121.60, 120.76, 105.53, 98.53, 93.68, 25.89 (3C), 25.87 (3C), 25.64 (3C), 18.55, 18.53, 18.41, -4.09 (2C), -4.17 (2C), -4.20 (2C). HRMS (ESI-I): calculated for $C_{33}H_{51}O_7Si_3$ [M-H]$^-$ 643.2943; found 643.2949.

(9Z,12Z,15Z)-3-(*tert*-butyldimethylsilyloxy)-2-(3,4-bis-(*tert*-butyldimethylsilyloxy)phenyl)-5-hydroxy-4-oxo-4*H*-chromen-7-yl octadeca-9,12,15-trienoate (17): To a solution of α-linolenic acid (94 mg, 0.34 mmol), dicyclohexylcarbodiimide (77 mg, 0.37 mmol) and 4-dimethylaminopyridine (21 mg, 0.173 mmol) in anhydrous CH_2Cl_2 (5 mL) stirred under N_2, was added a solution of compound **16** (218 mg, 0.34 mmol) in anhydrous CH_2Cl_2 (4 mL). The resulting mixture was stirred at room temperature

for 1 h and cooled to 4 °C for 1 h to afford filtration of the precipitate. The filtrate was washed with water and brine, dried over $MgSO_4$, filtered and evaporated under reduced pressure. Purification of the residue was performed by silica column chromatography using pentane/EtOAc (99:1 v/v) as eluent to give compound **17** (126 mg, 41%) as yellow oil. R_f (pentane/EtOAc, 90:10) = 0.67. ^{1}H NMR (500 MHz, $CDCl_3$) δ 7.45 (dd, *J* = 8.4, 2.2 Hz, 1H, H6'), 7.34 (d, *J* = 2.2 Hz, 1H, H2'), 6.90 (d, *J* = 8.4 Hz, 1H, H5'), 6.69 (d, *J* = 2.0 Hz, 1H, H8), 6.49 (d, *J* = 2.0 Hz, 1H, H6), 5.46 – 5.26 (m, 6H, 3 × CH=CH), 2.81 (t, *J* = 5.9 Hz, 4H, H11'', H14''), 2.58 (t, *J* = 7.5 Hz, 2H, H2''), 2.12 – 2.03 (m, 4H, H8'', H17''), 1.76 (qt, *J* = 7.5 Hz, 2H, H3''), 1.45 – 1.31 (m, 8H, H4'', H5'', H6'', H7''), 1.00 (s, 9H, 3 × CH_3*t*Bu), 1.00 (s, 9H, 3 × CH_3*t*Bu), 0.97 (t, *J* = 7.5 Hz, 2H, H18''), 0.83 (s, 9H, 3 × CH_3*t*Bu), 0.23 (s, 6H, 2 × CH_3Si), 0.20 (s, 6H, 2 × CH_3Si), 0.11 (s, 6H, 2 × CH_3Si). ^{13}C NMR (125 MHz, $CDCl_3$) δ 178.49, 171.46, 161.66, 155.52, 155.50, 154.23, 149.54, 146.82, 135.98, 131.95, 130.20, 128.28, 128.22, 127.77, 127.08, 123.99, 123.37, 121.75, 120.83, 108.84, 104.28, 100.66, 34.38, 29.55, 29.13, 29.07, 29.01, 27.17, 25.91 (3C), 25.88 (3C), 25.62 (3C), 25.60, 25.51, 24.79, 20.54, 18.58, 18.56, 18.41, 14.28, -4.06 (2C), -4.16 (2C), -4.17 (2C). HRMS (ASAP+I): calculated for $C_{32}H_{43}O_4$ $[M + H]^+$ 905.5239; found 905.5228.

(9*Z*,12*Z*,15*Z*)-2-(3,4-dihydroxyphenyl)-3,5-dihydroxy-4-oxo-4*H*-chromen-7-yl octadeca-9,12,15-trienoate (Quer-7-ALA, 18): To a solution of compound **17** (115 mg, 0.13 mmol) in anhydrous THF (9.5 mL) stirred under N_2, was added dropwise triethylamine trihydrofluoride (186 µL, 1.14 mmol). The resulting mixture was stirred at room temperature for 35 min, diluted in EtOAc and washed with water and brine. The organic layer was dried over $MgSO_4$, filtered and evaporated under reduced pressure. Purification of the residue was performed by silica column chromatography using pentane/EtOAc (90:10 to 80:20 v/v) as eluent to give compound **18** (30 mg, 61%) as bright yellow solid. R_f (pentane/EtOAc, 50:50) = 0.61. Mp 235.8 °C. ^{1}H NMR (500 MHz, MeOD) δ 7.77 (d, *J* = 2.0 Hz, 1H, H2'), 7.66 (dd, *J* = 8.5, 2.0 Hz, 1H, H6'), 6.88 (d, *J* = 8.5 Hz, 1H, H5'), 6.81 (d, *J* = 1.9 Hz, 1H, H8), 6.46 (d, *J* = 1.9 Hz, 1H, H6), 5.45 – 5.21 (m, 6H, 3 × CH=CH), 2.85 – 2.73 (m, 4H, H11'', H14''), 2.58 (t, *J* = 7.4 Hz, 2H, H2''), 2.13 – 2.01 (m, 4H, H8'', H17''), 1.72 (qt, *J* = 7.4 Hz, 2H, H3''), 1.48 – 1.30 (m, 8H, H4'', H5'', H6'', H7''), 0.96 (t, *J* = 7.5 Hz, 3H, H18''). ^{13}C NMR (125 MHz, MeOD) δ 177.63, 172.84, 161.97, 157.20, 156.76, 149.32, 149.21, 146.26, 138.03, 132.71, 131.05, 129.20, 129.18, 128.90, 128.23, 123.71, 122.01, 116.26, 116.23, 108.50, 104.83, 101.87, 35.02, 30.66, 30.23, 30.16, 30.09, 28.14, 26.52, 26.40, 25.80, 21.48, 14.67. HRMS (ESI-I): calculated for $C_{33}H_{37}O_8$ $[M-H]^-$ 561.2488; found 561.2490.

__**Figure S1** : Graphical representation of *in vitro* evaluations of lipophenols.__

TOX = cytotoxicity assays represented in cell survival (%) depending on derivative concentration (µM). Results are normalized with non-treated control cells as 100% survival (red line).

ROS = Inhibition of ROS assays represented in ROS production (%) depending on derivative concentration (µM). Results are normalized with non-treated control cells exposed to stressor as 100% of ROS production (red line).

	TOX	ROS
phloroglucinol 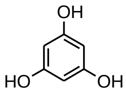	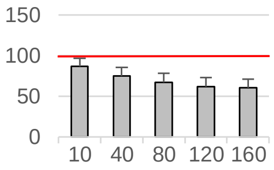	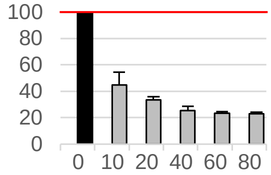
phloro-LA (**3**) 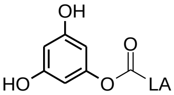	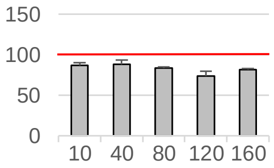	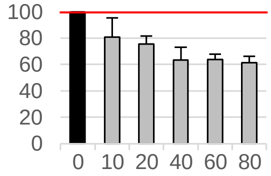
resveratrol 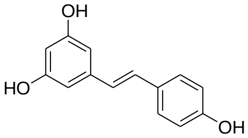	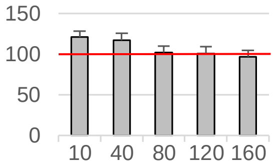	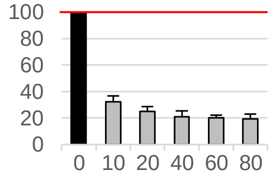
resv-4'-LA (**8**) 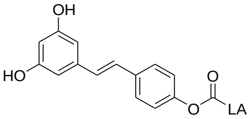	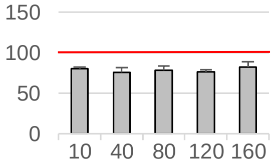	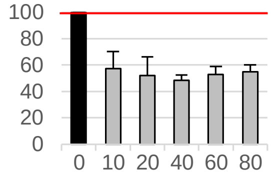
(+)-catechin 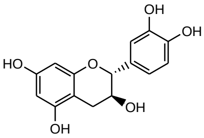	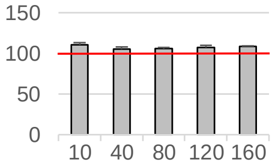	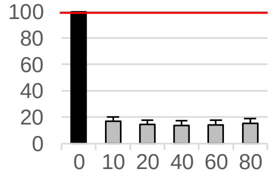
cat-3-LA (**9**) 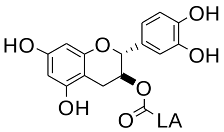	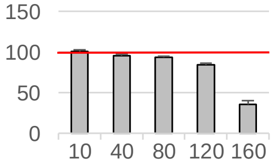	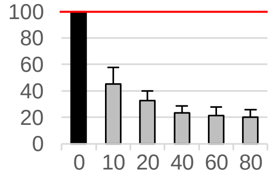

	TOX	ROS
quercetin 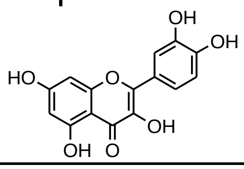	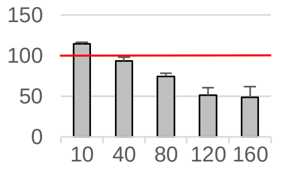	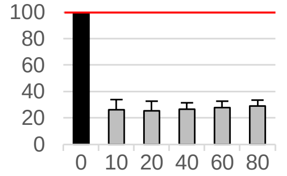
quer-3-LA (**13**) 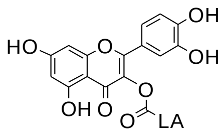	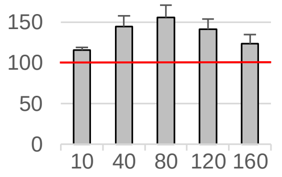	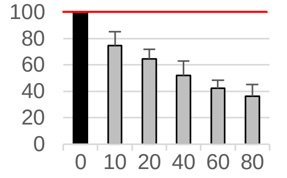
quer-3-DHA (**15**) 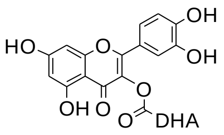	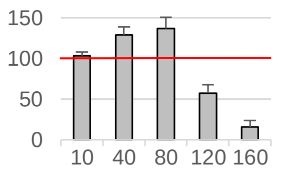	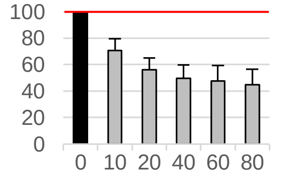
quer-3-ALA (**14**) 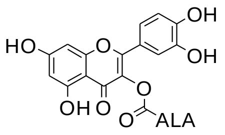	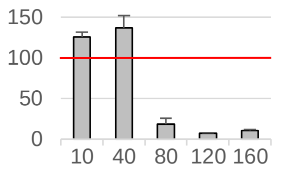	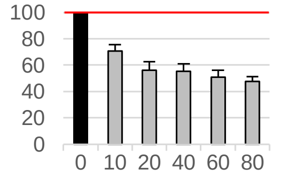
quer-7-ALA (**18**) 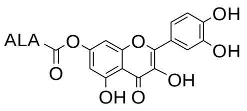	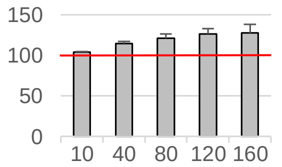	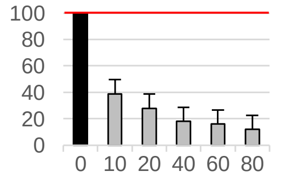

Figure S2: Full NMR spectra characterization of derivatives from 2 to 18 (^{1}H and ^{13}C).

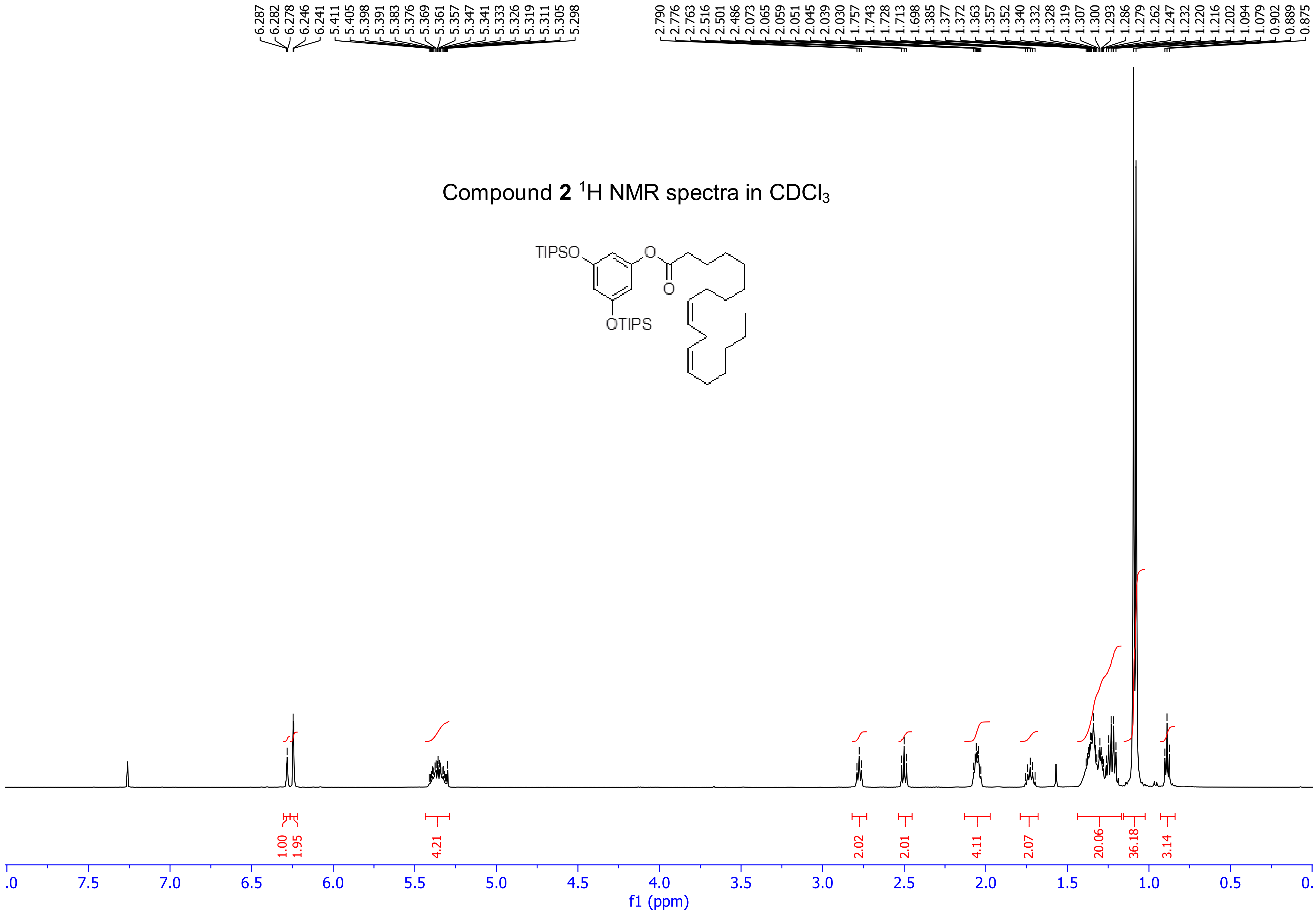
Compound 2 1H NMR spectra in $CDCl_3$
TIPSO
OTIPS
f1 (ppm)

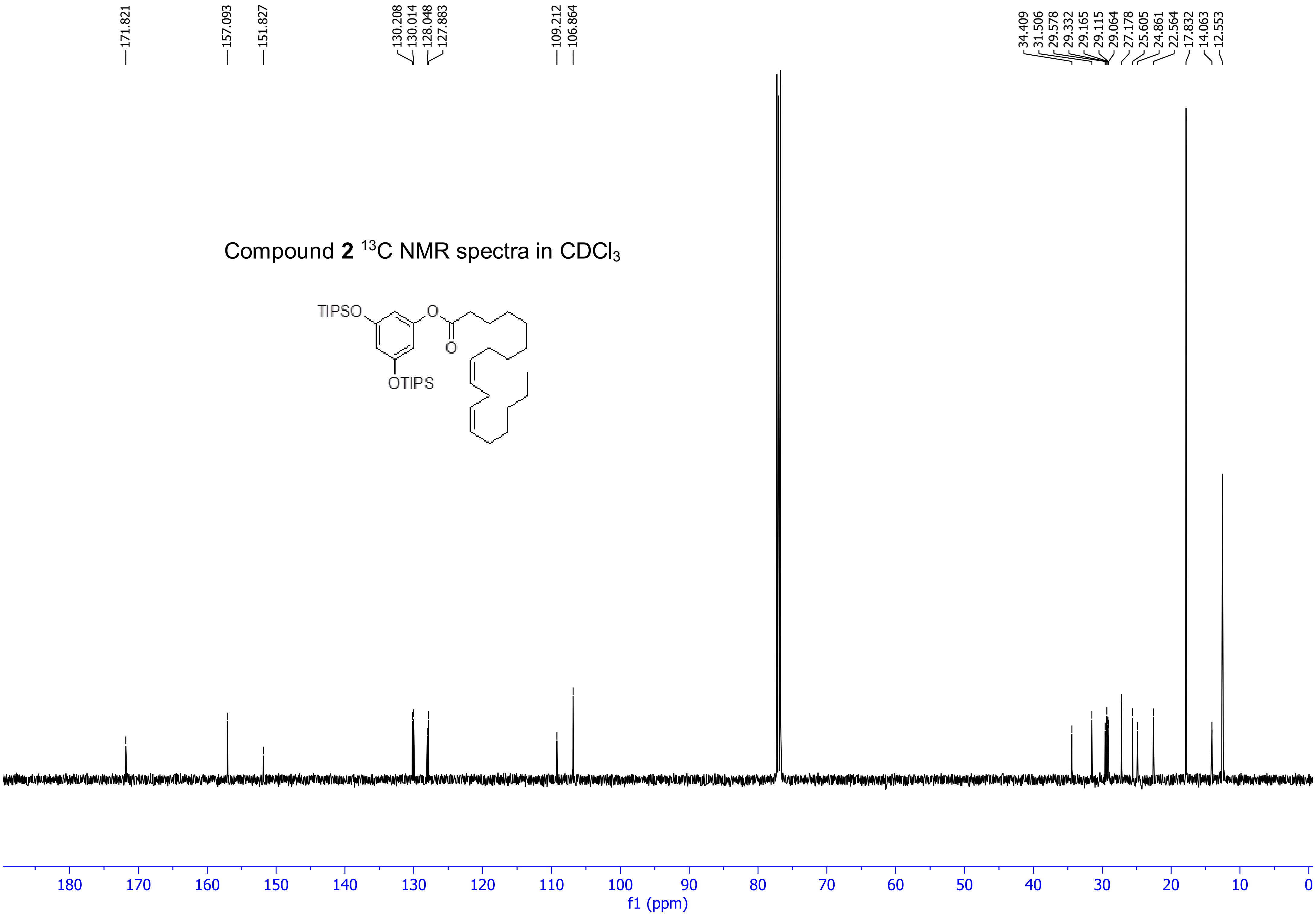
171.821
157.093
151.827
130.208
130.014
128.048
127.883
109.212
106.864
34.409
31.506
29.578
29.332
29.165
29.115
29.064
27.178
25.605
24.861
22.564
17.832
14.063
12.553
Compound 2 13C NMR spectra in CDCl3
TIPSO
O
O
OTIPS
180
170
160
150
140
130
120
110
100
90
80
70
60
50
40
30
20
10
0
f1 (ppm)

Phloro-LA (compound **3**) ^{1}H NMR spectra in $CDCl_3$

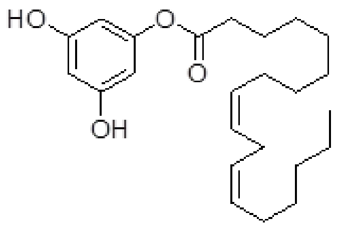

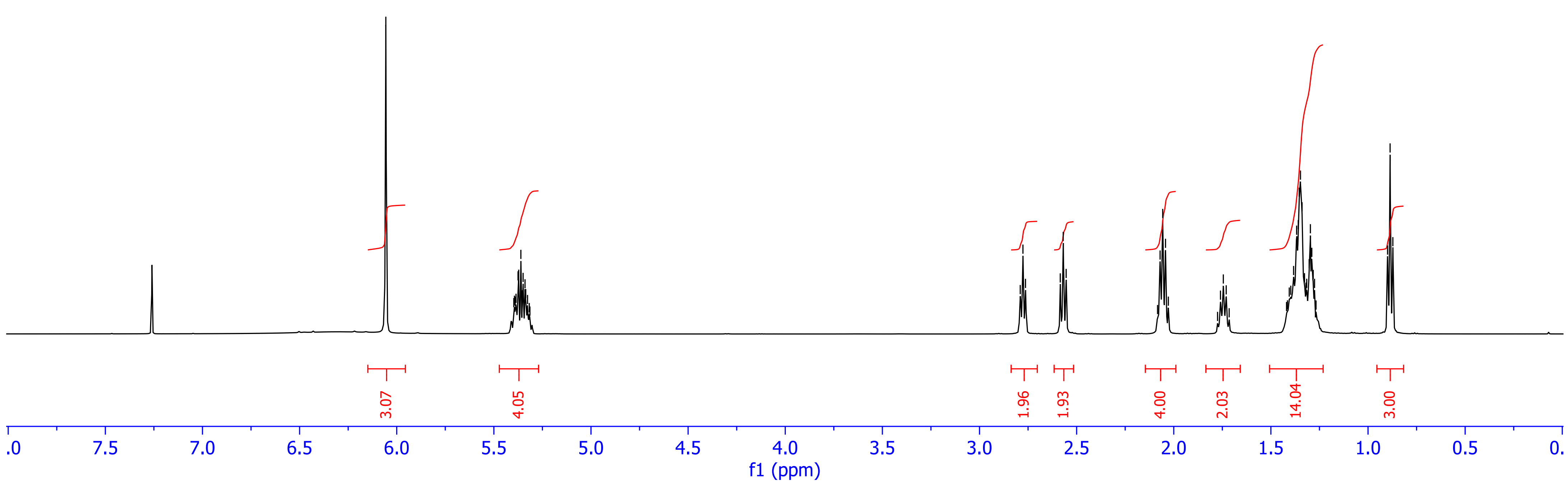

Phloro-LA (compound **3**) ^{13}C NMR spectra in $CDCl_3$

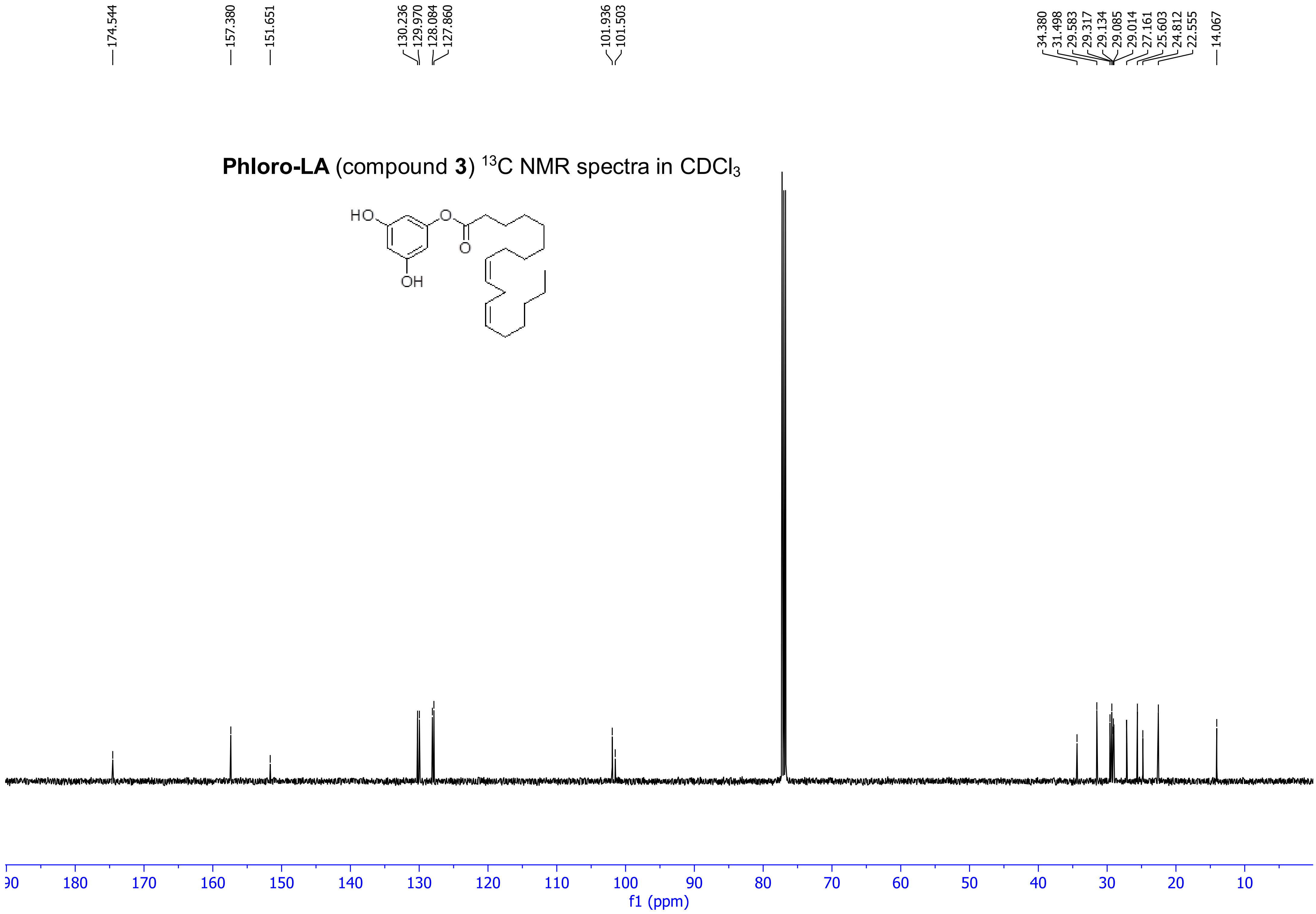

Compound **4** ^{1}H NMR spectra in CD_3OD

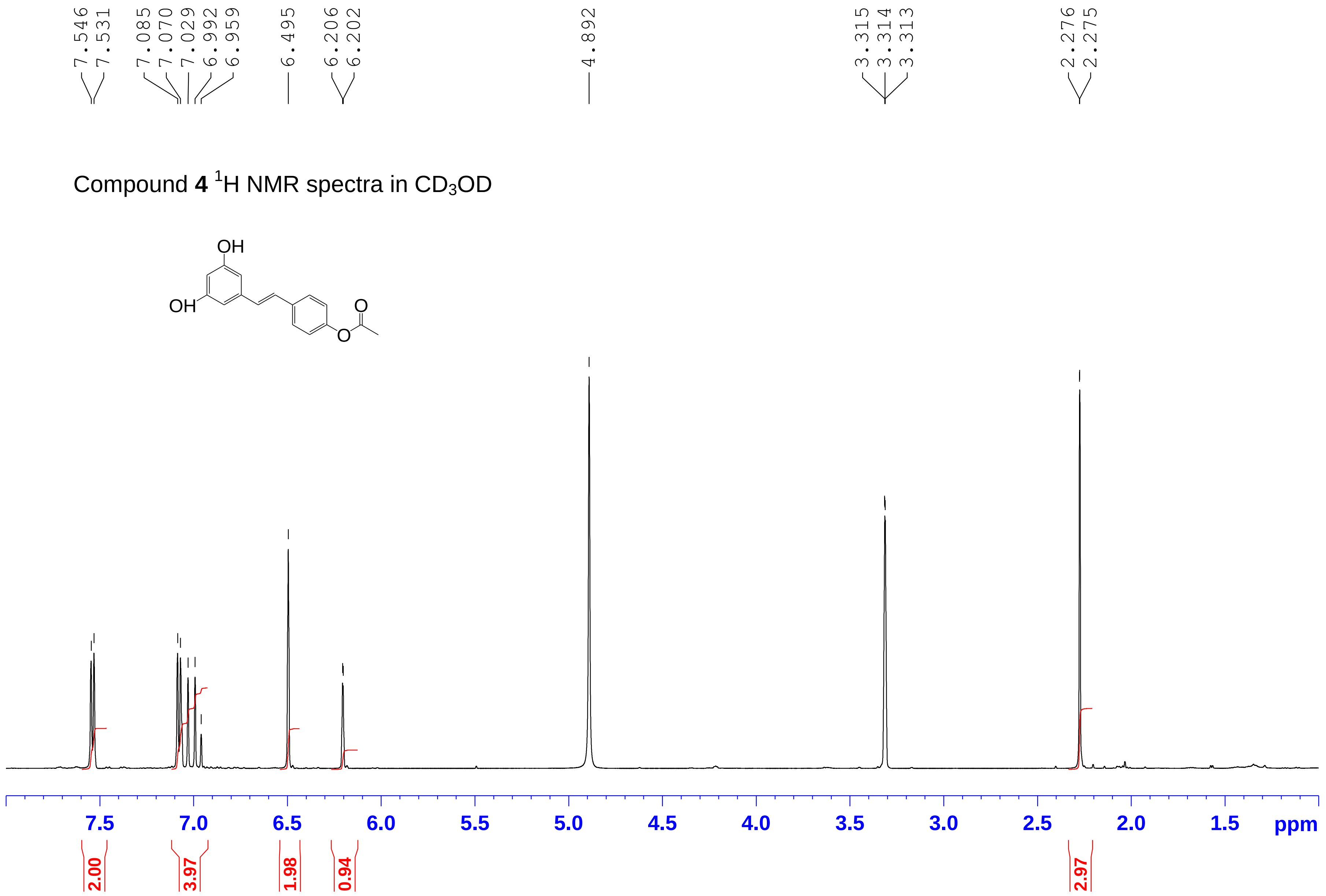

Compound **4** ^{13}C Jmod NMR spectra in CD_3OD

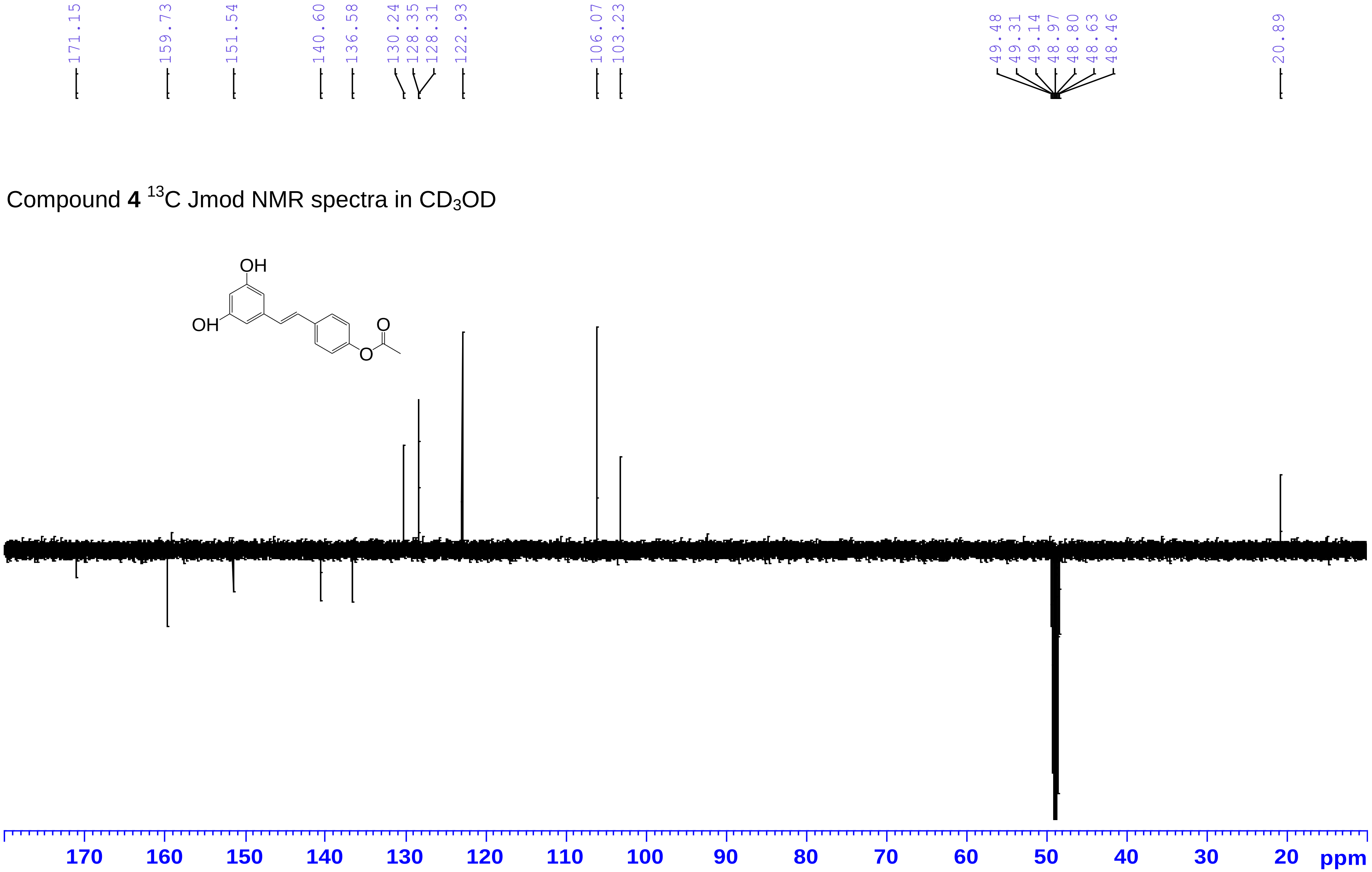

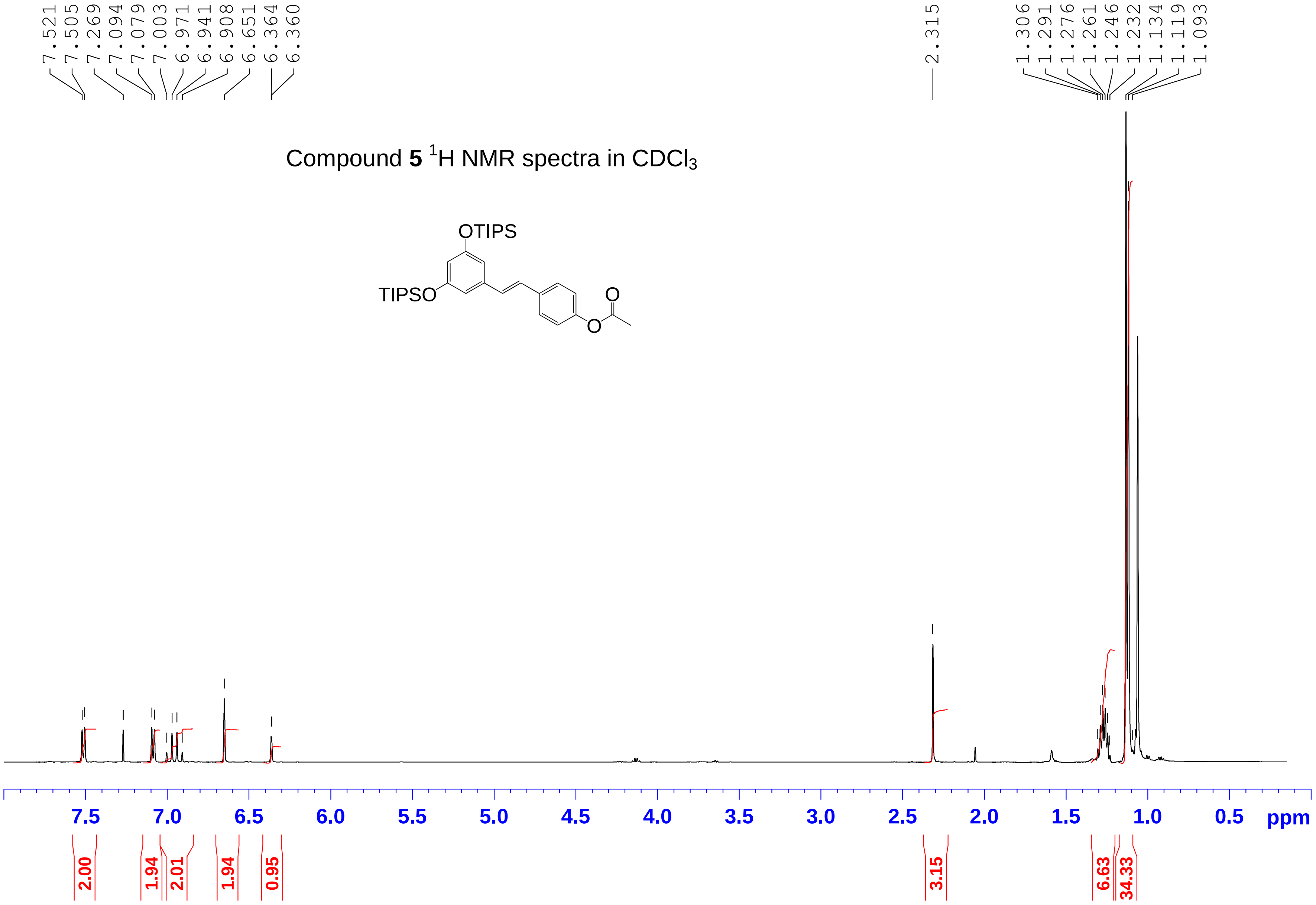
Compound 5 ^{1}H NMR spectra in $CDCl_3$
OTIPS
TIPSO
O
O
7.521
7.505
7.269
7.094
7.079
7.003
6.971
6.941
6.908
6.651
6.364
6.360
2.315
1.306
1.291
1.276
1.261
1.246
1.232
1.134
1.119
1.093
7.5
7.0
6.5
6.0
5.5
5.0
4.5
4.0
3.5
3.0
2.5
2.0
1.5
1.0
0.5
ppm
2.00
1.94
2.01
1.94
0.95
3.15
6.63
34.33

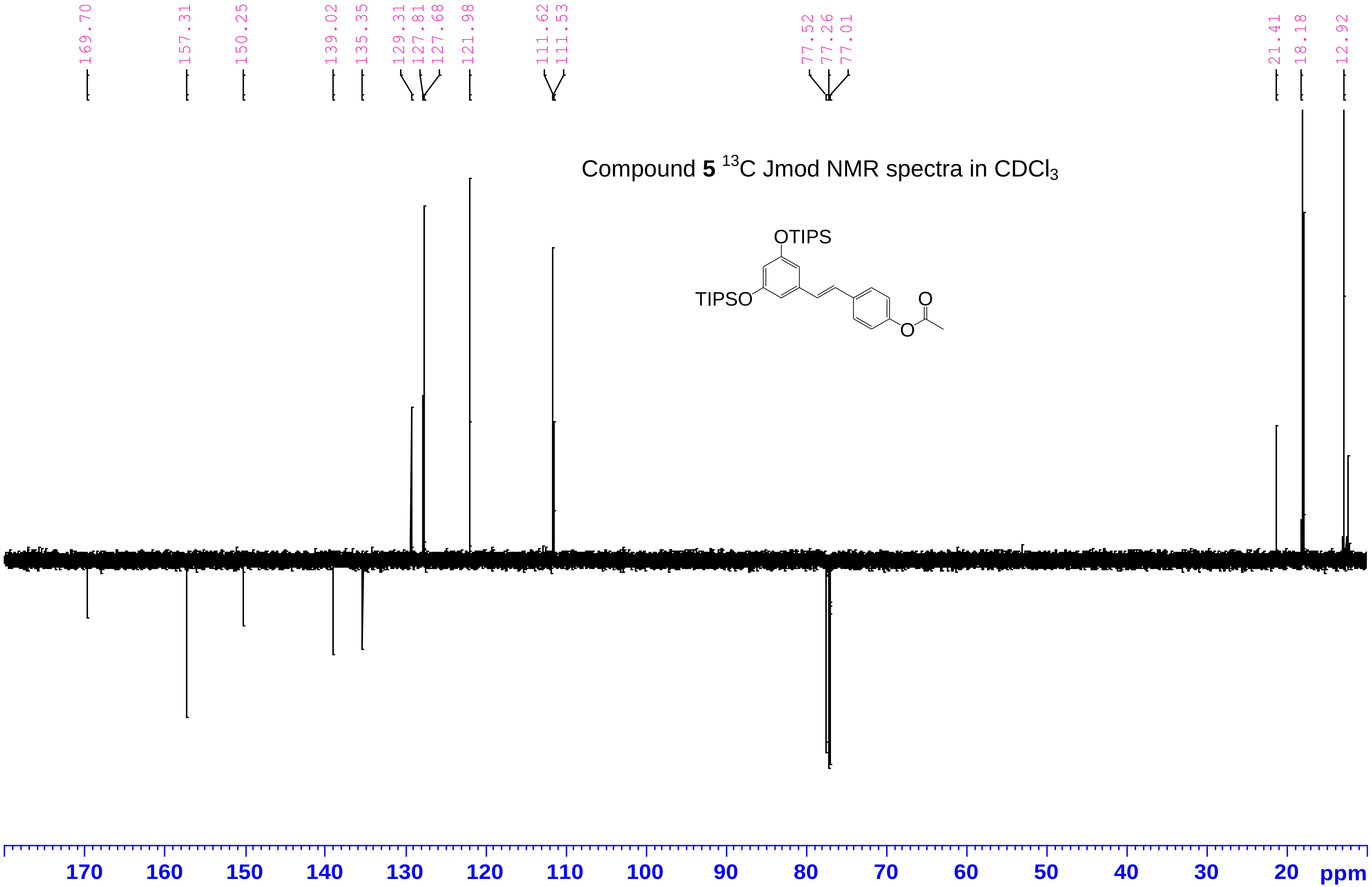
Compound 5 13C Jmod NMR spectra in CDCl3
OTIPS
TIPSO
O
O
169.70
157.31
150.25
139.02
135.35
129.31
127.81
127.68
121.98
111.62
111.53
77.52
77.26
77.01
21.41
18.18
12.92
170
160
150
140
130
120
110
100
90
80
70
60
50
40
30
20
ppm

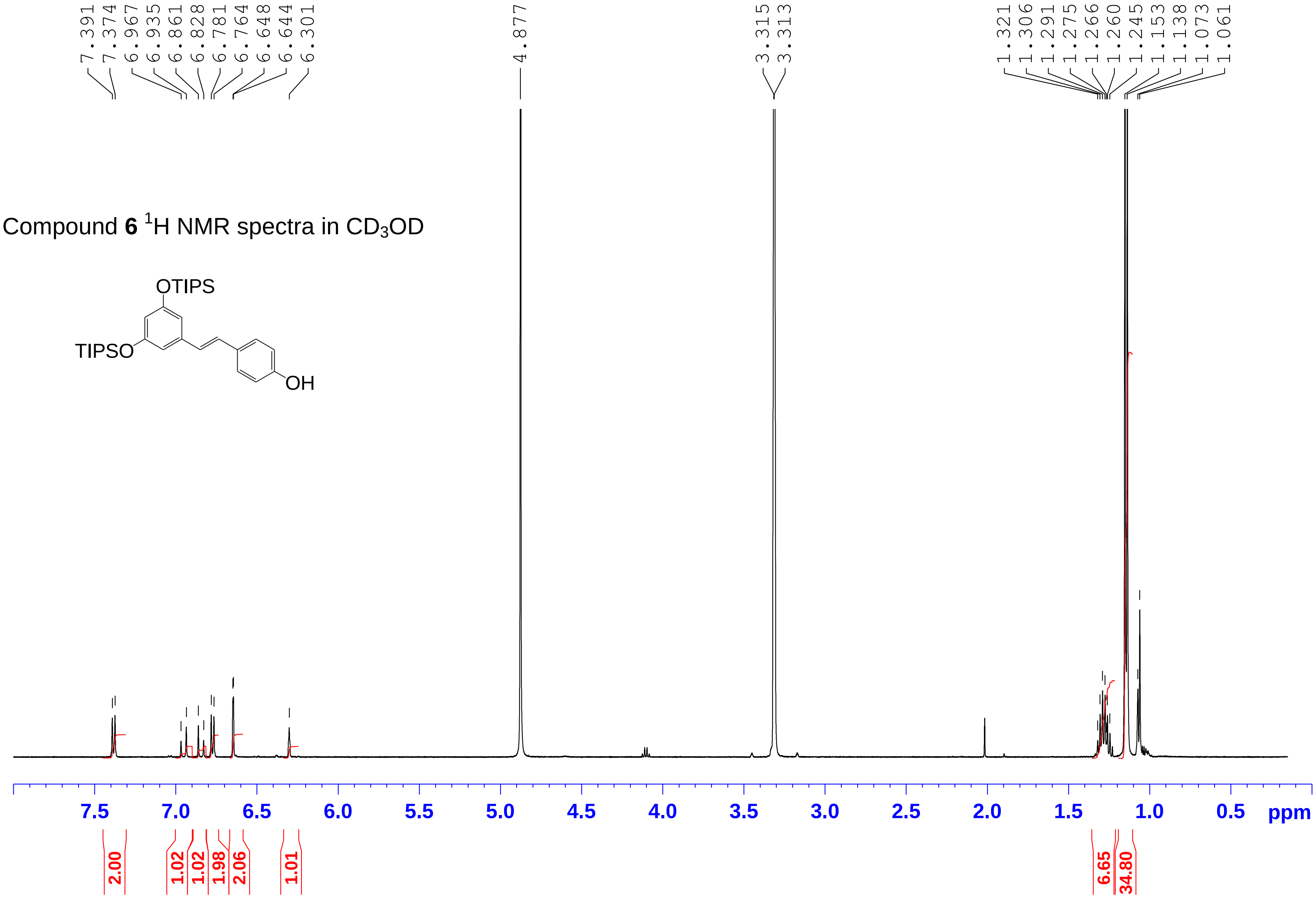
Compound 6 1H NMR spectra in CD3OD
OTIPS
TIPSO
OH
7.391
7.374
6.967
6.935
6.861
6.828
6.781
6.764
6.648
6.644
6.301
4.877
3.315
3.313
1.321
1.306
1.291
1.275
1.266
1.260
1.245
1.153
1.138
1.073
1.061
7.5
7.0
6.5
6.0
5.5
5.0
4.5
4.0
3.5
3.0
2.5
2.0
1.5
1.0
0.5
ppm
2.00
1.02
1.02
1.98
2.06
1.01
6.65
34.80

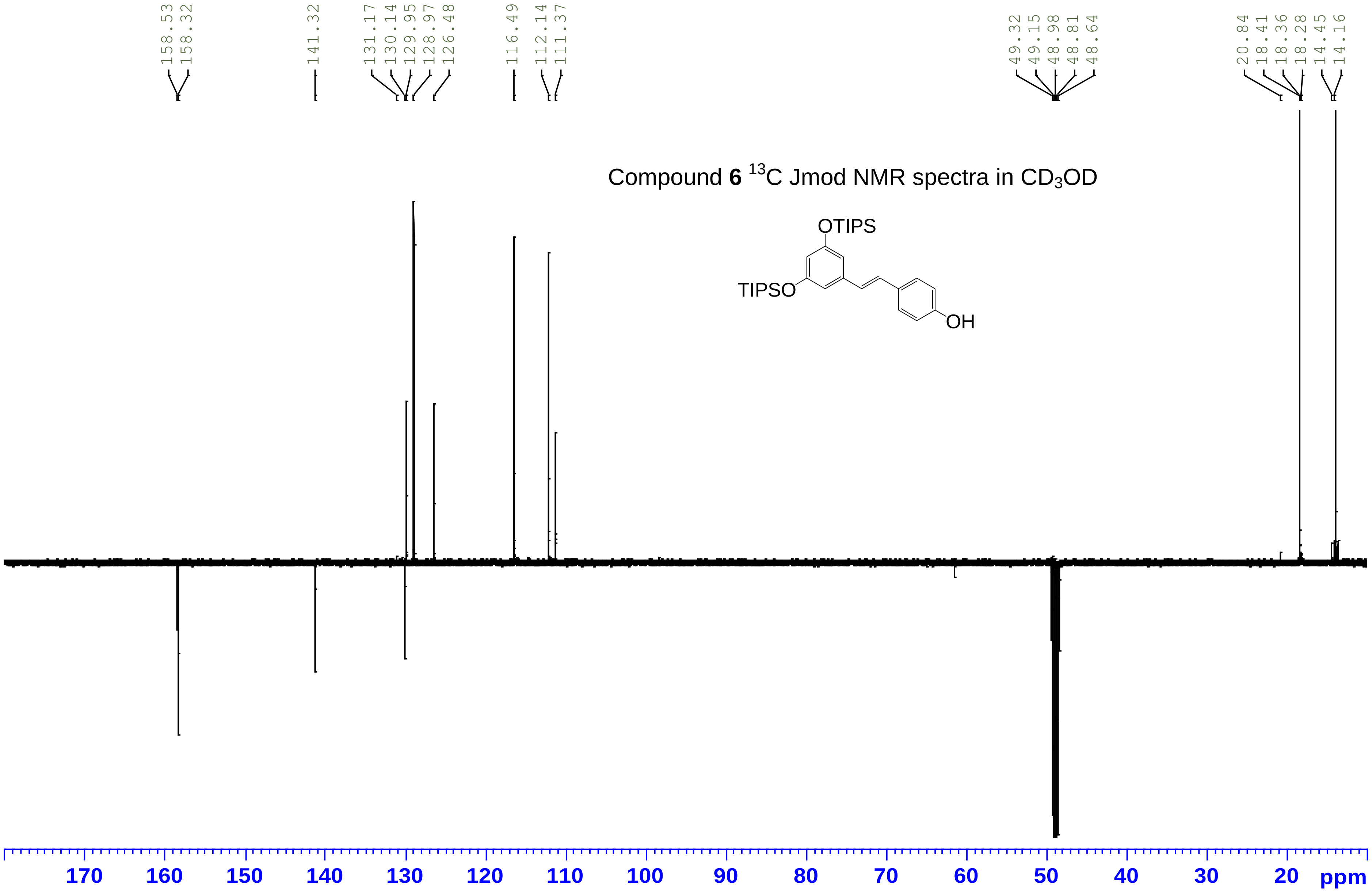
158.53
158.32
141.32
131.17
130.14
129.95
128.97
126.48
116.49
112.14
111.37
49.32
49.15
48.98
48.81
48.64
20.84
18.41
18.36
18.28
14.45
14.16
Compound 6 13C Jmod NMR spectra in CD3OD
OTIPS
TIPSO
OH
170
160
150
140
130
120
110
100
90
80
70
60
50
40
30
20
ppm

Compound **7** ^{1}H NMR spectra in $CDCl_3$

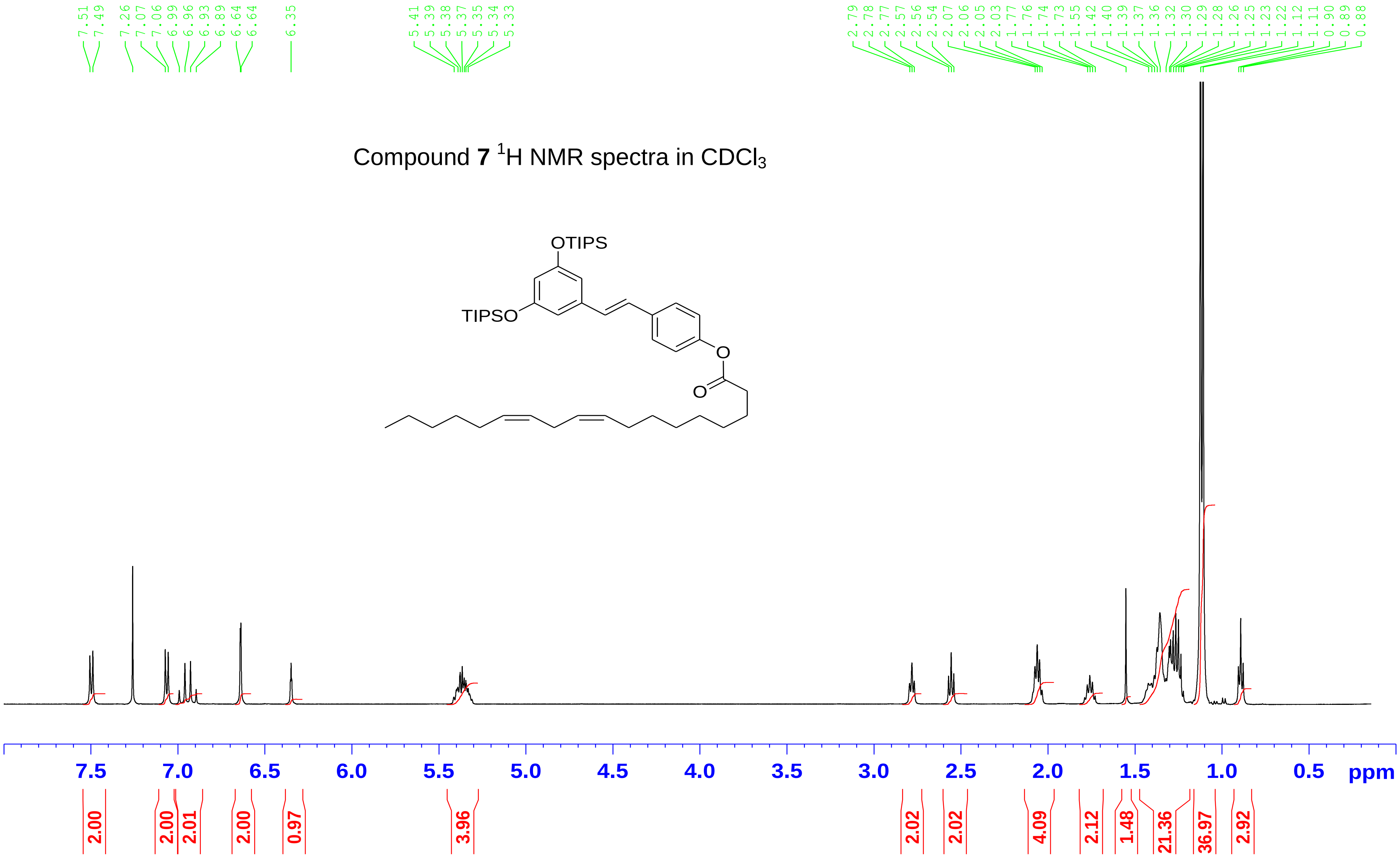

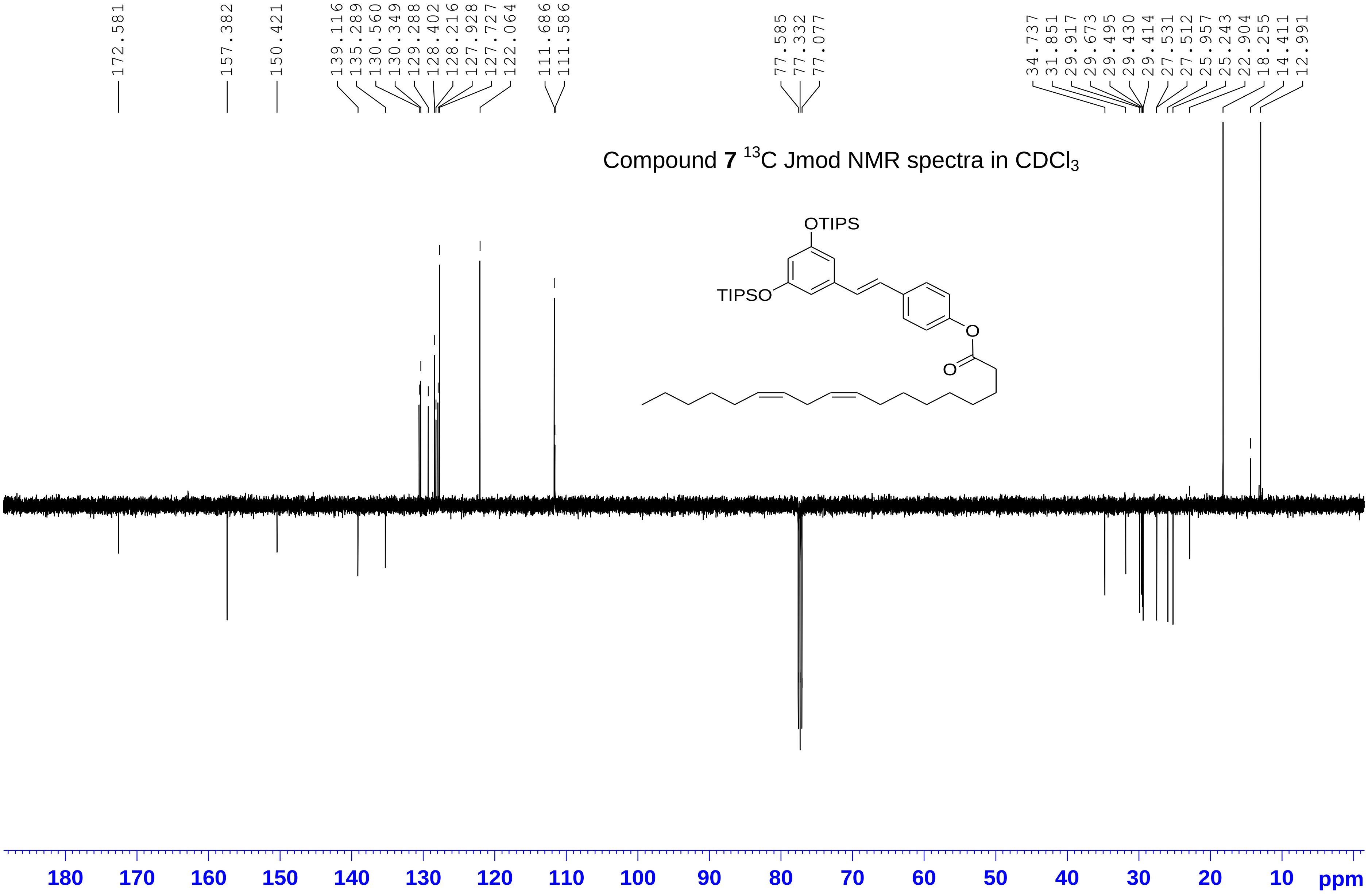
172.581
157.382
150.421
139.116
135.289
130.560
130.349
129.288
128.402
128.216
127.928
127.727
122.064
111.686
111.586
77.585
77.332
77.077
34.737
31.851
29.917
29.673
29.495
29.430
29.414
27.531
27.512
25.957
25.243
22.904
18.255
14.411
12.991
Compound **7** ^{13}C Jmod NMR spectra in $CDCl_3$
OTIPS
TIPSO
O
O
180 170 160 150 140 130 120 110 100 90 80 70 60 50 40 30 20 10 ppm

Resve-4'-LA (Compound **8**) ^1H NMR spectra in $CDCl_3$

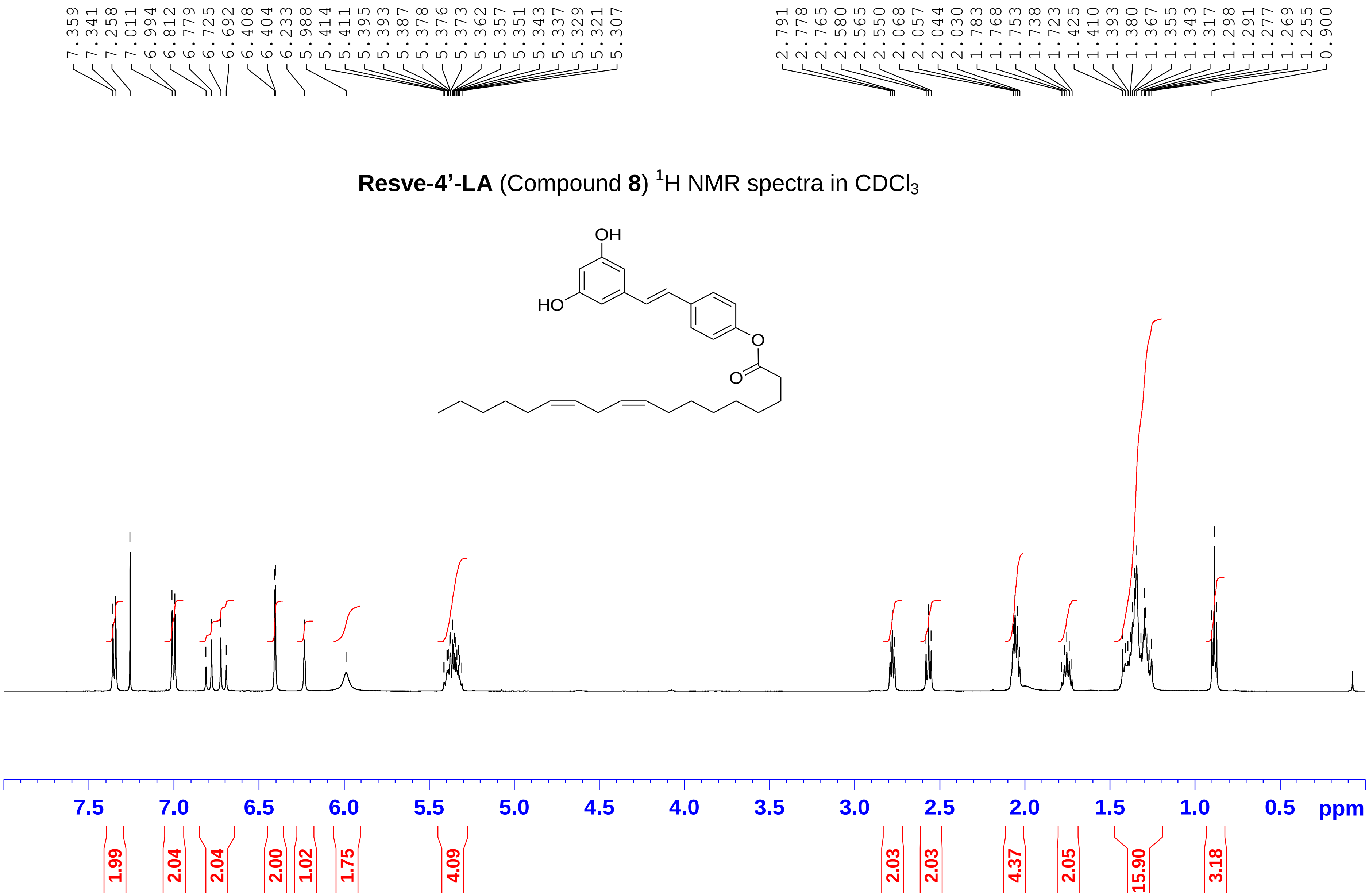

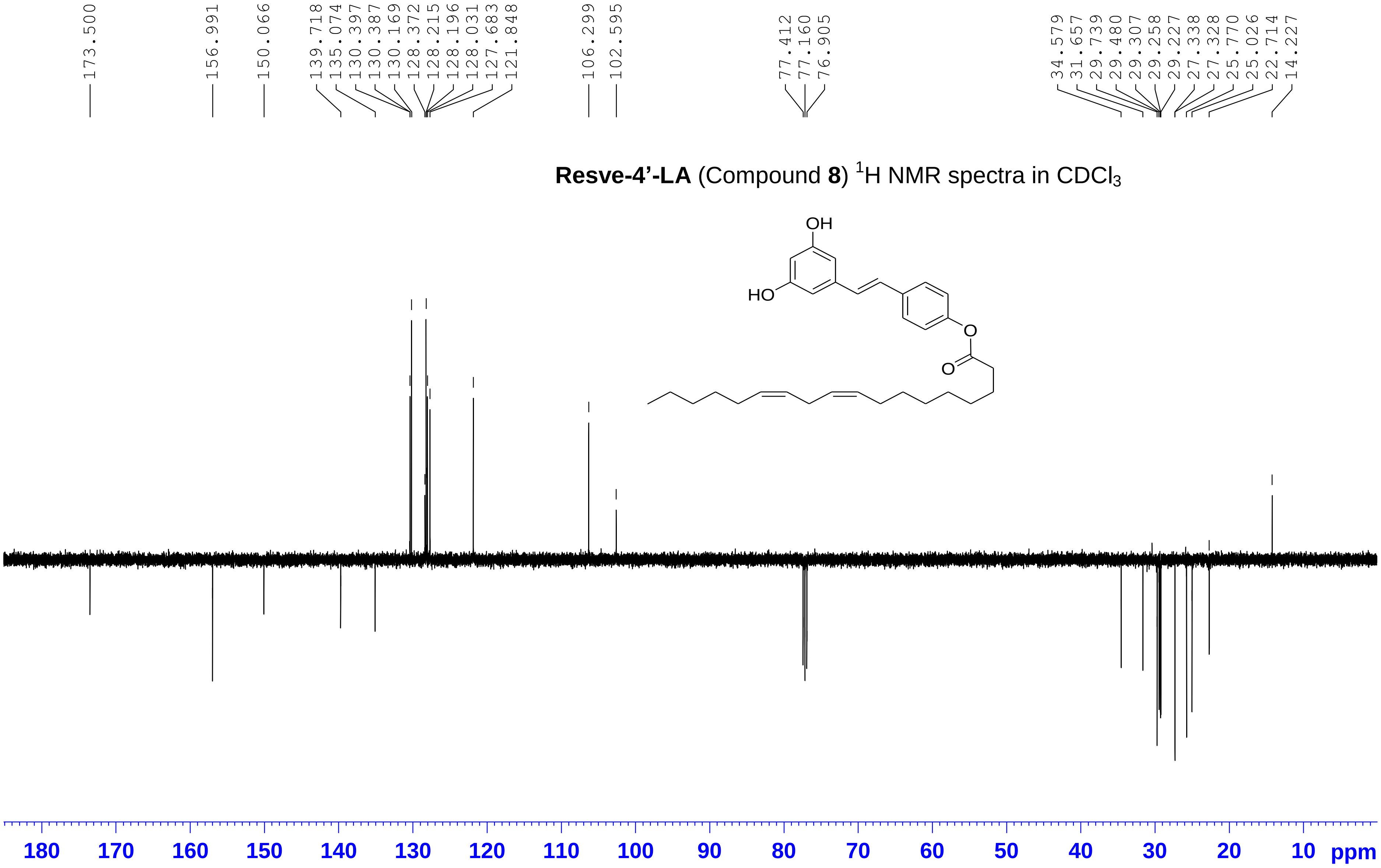

173.500
156.991
150.066
139.718
135.074
130.397
130.387
130.169
128.372
128.215
128.196
128.031
127.683
121.848
106.299
102.595
77.412
77.160
76.905
34.579
31.657
29.739
29.480
29.307
29.258
29.227
27.338
27.328
25.770
25.026
22.714
14.227
Resve-4'-LA (Compound **8**) ^{1}H NMR spectra in $CDCl_3$
OH
HO
O
O
180 170 160 150 140 130 120 110 100 90 80 70 60 50 40 30 20 10 ppm

Cat-3-LA (compound **9**) ^{1}H NMR spectra in MeOD

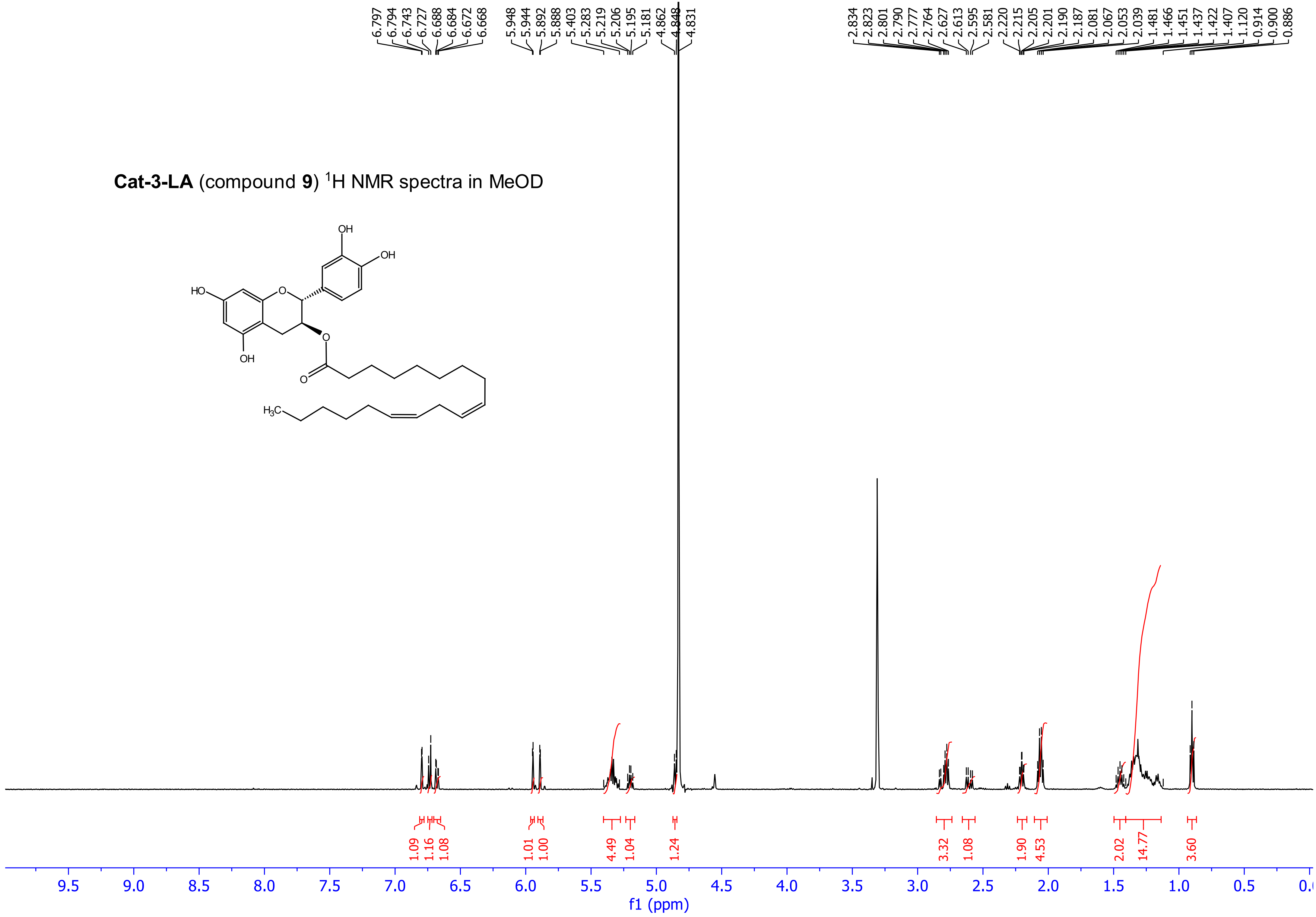

Cat-3-LA (compound **9**) ^{13}C Jmod NMR spectra in MeOD

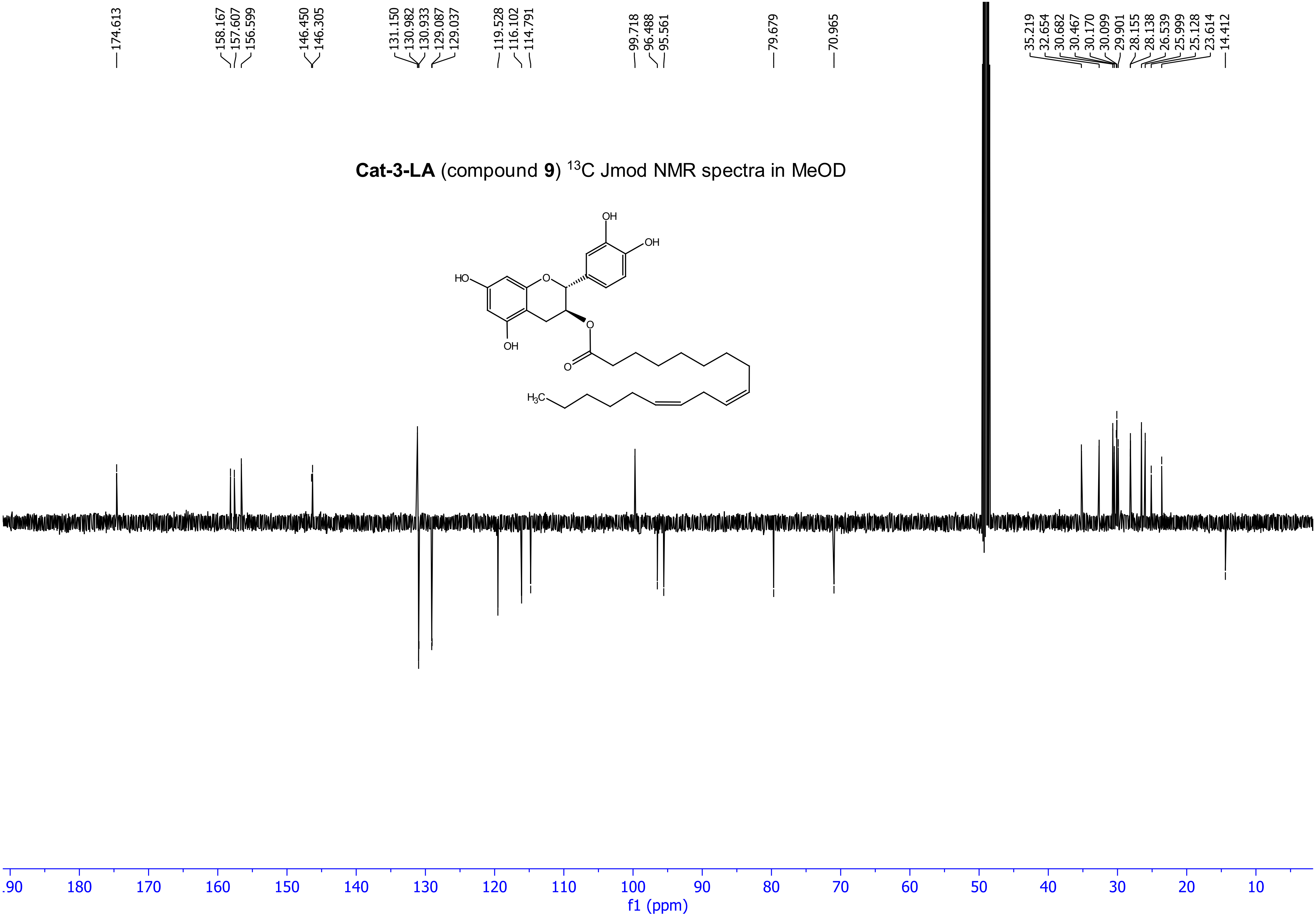

Compound **10** ^{1}H NMR spectra in $CDCl_3$

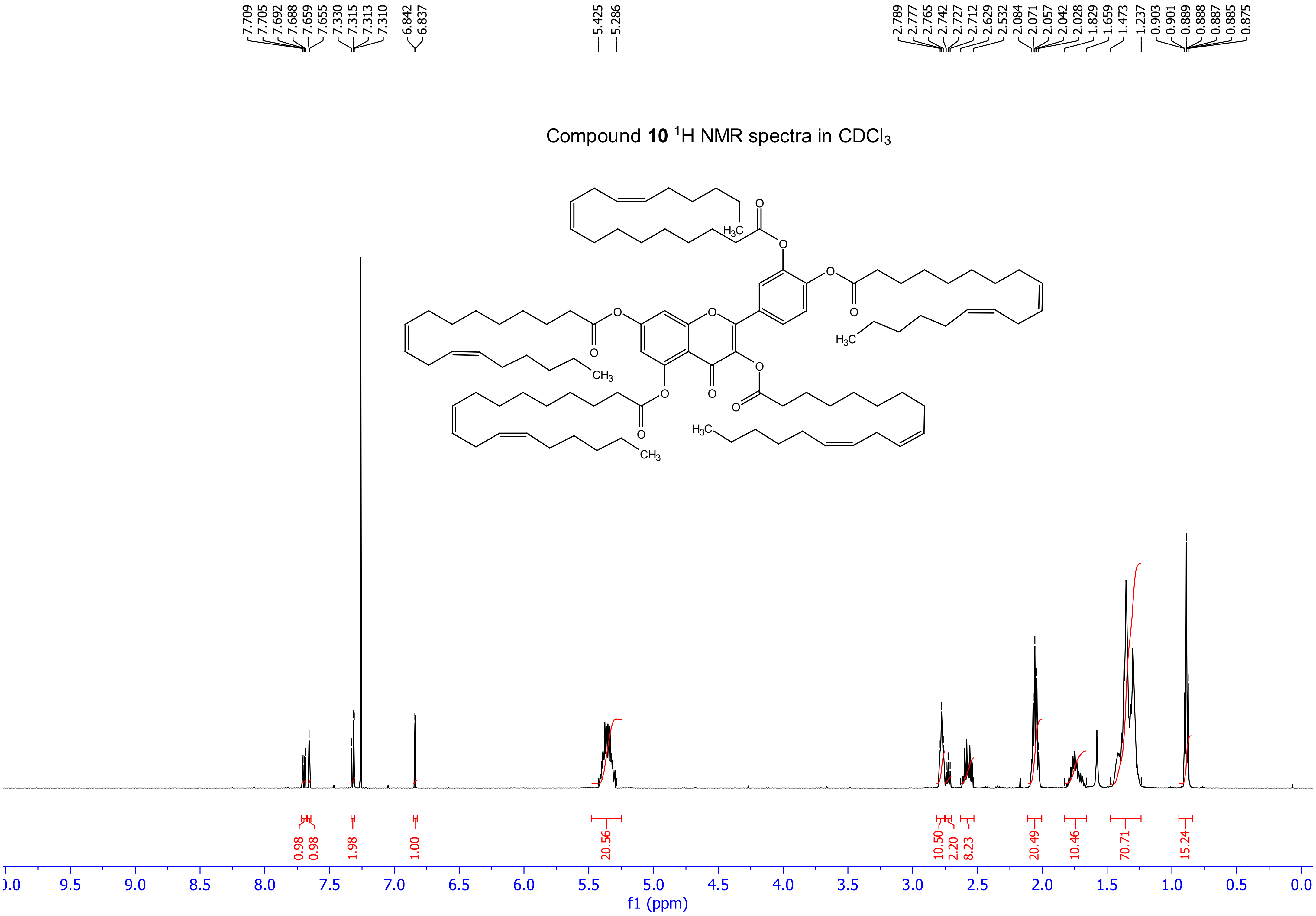

Compound **10** ^{13}C NMR spectra in $CDCl_3$

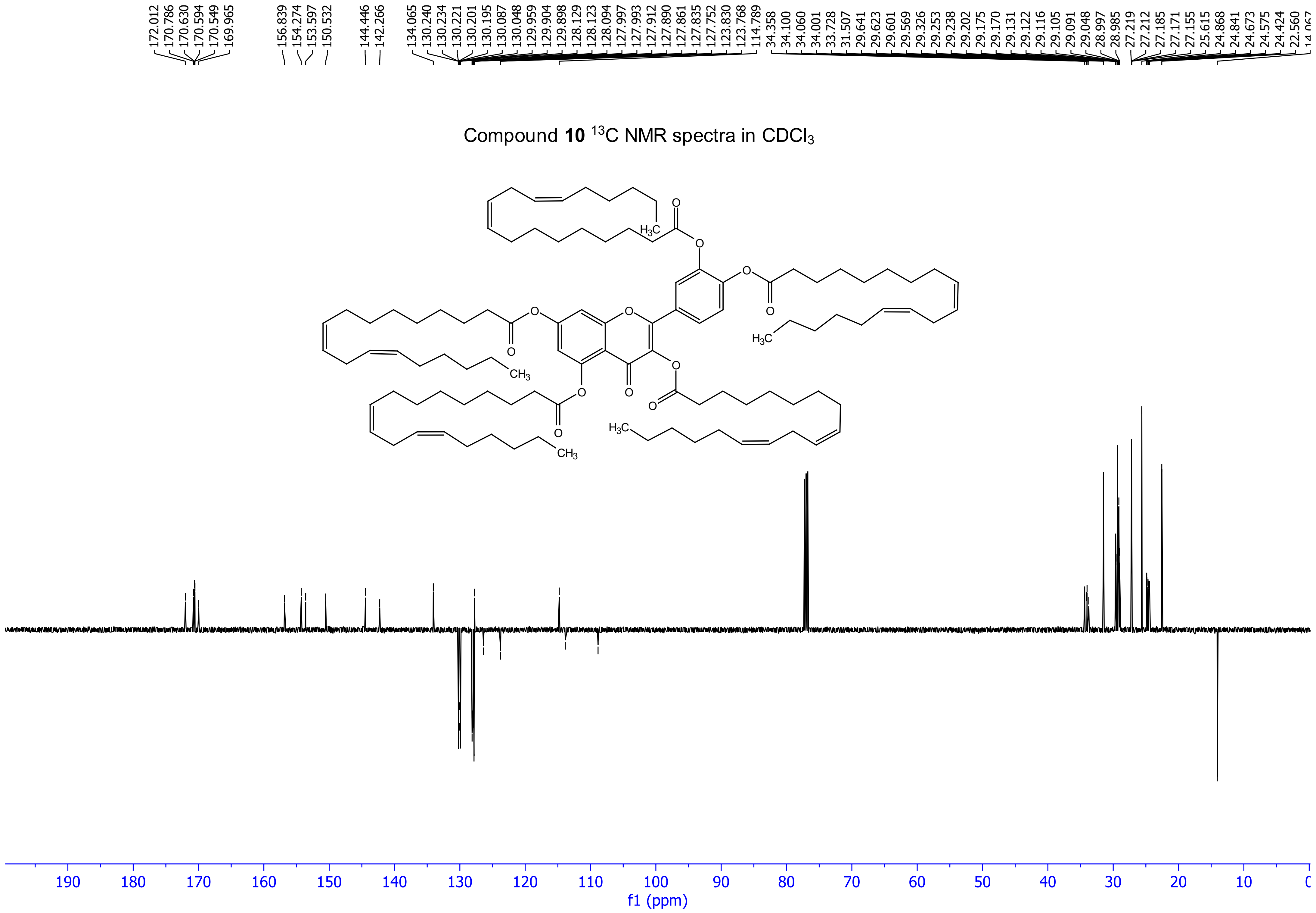

Compound **11** ^{1}H NMR spectra in $CDCl_3$

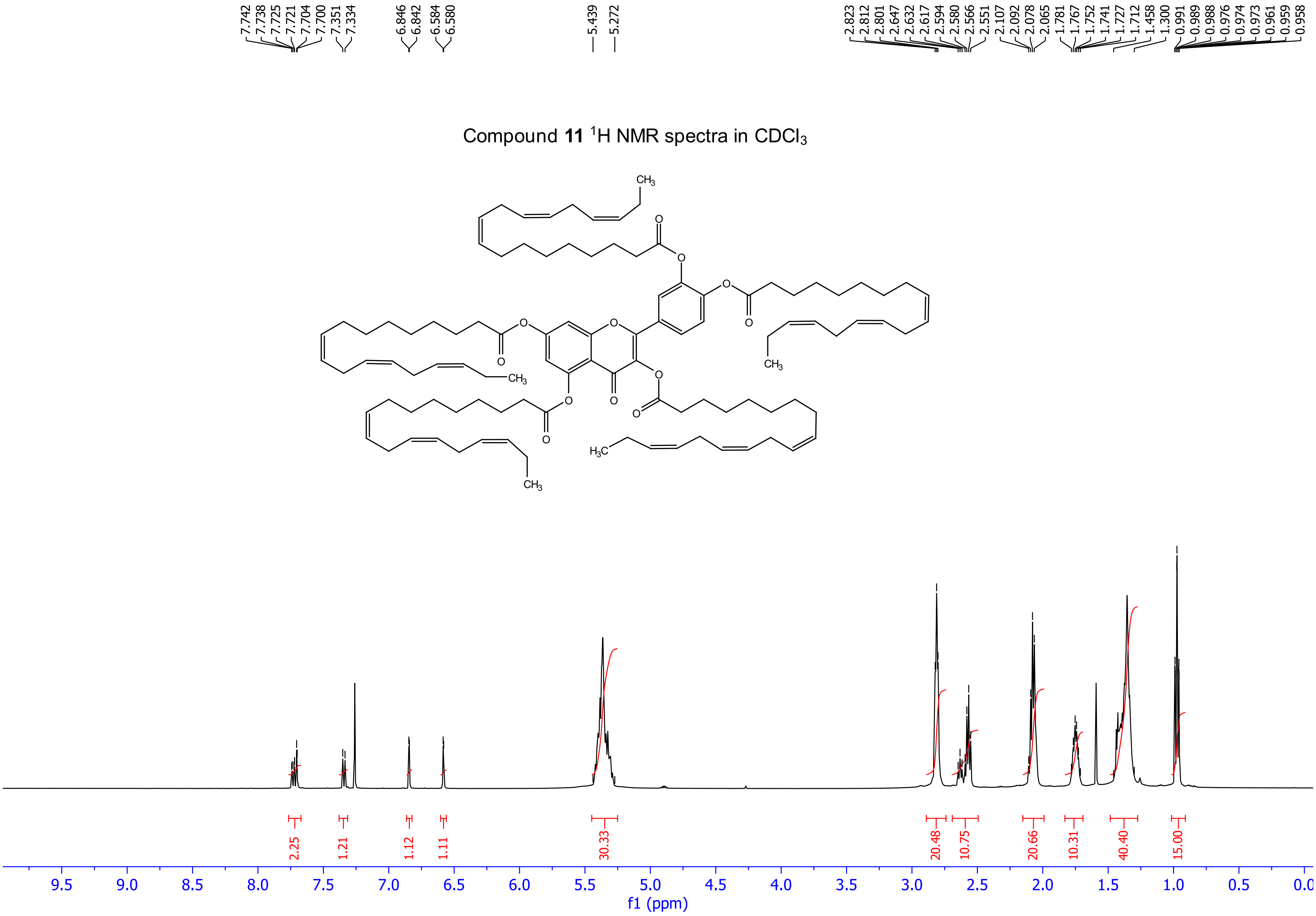

176.257, 171.070, 170.622, 170.584, 170.511, 161.668, 156.407, 155.906, 155.472, 144.714, 142.304, 132.189, 131.928, 131.918, 130.237, 130.171, 130.094, 130.081, 128.289, 128.253, 128.253, 128.212, 128.192, 128.152, 127.804, 127.753, 127.685, 127.469, 127.071, 127.063, 127.044, 126.457, 123.952, 123.927, 108.711, 105.440, 101.122, 34.356, 34.031, 33.986, 33.684, 29.581, 29.570, 29.531, 29.215, 29.181, 29.105, 29.066, 29.034, 28.981, 28.953, 28.819, 27.188, 27.163, 25.585, 25.494, 24.843, 24.800, 24.711, 24.613, 20.522, 14.260

Compound **11** ^{13}C Jmod NMR spectra in $CDCl_3$

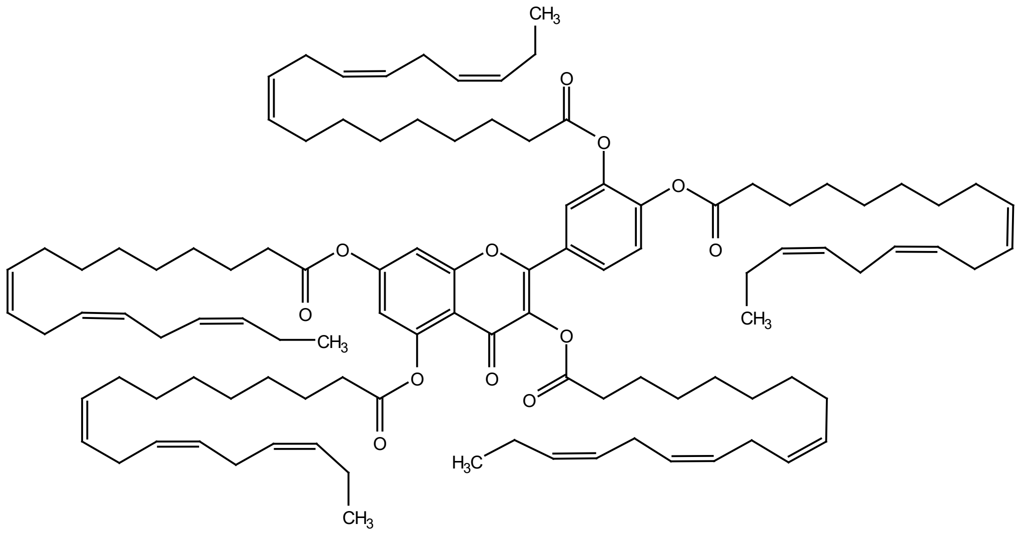

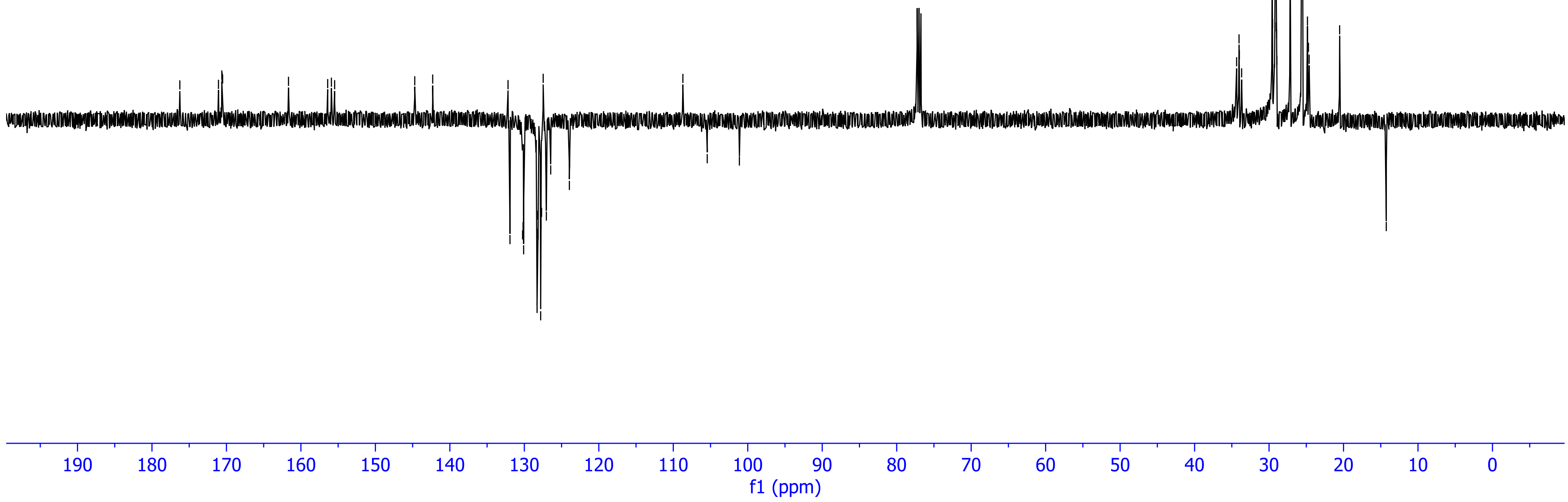

Compound **12** ^{1}H NMR spectra in $CDCl_3$

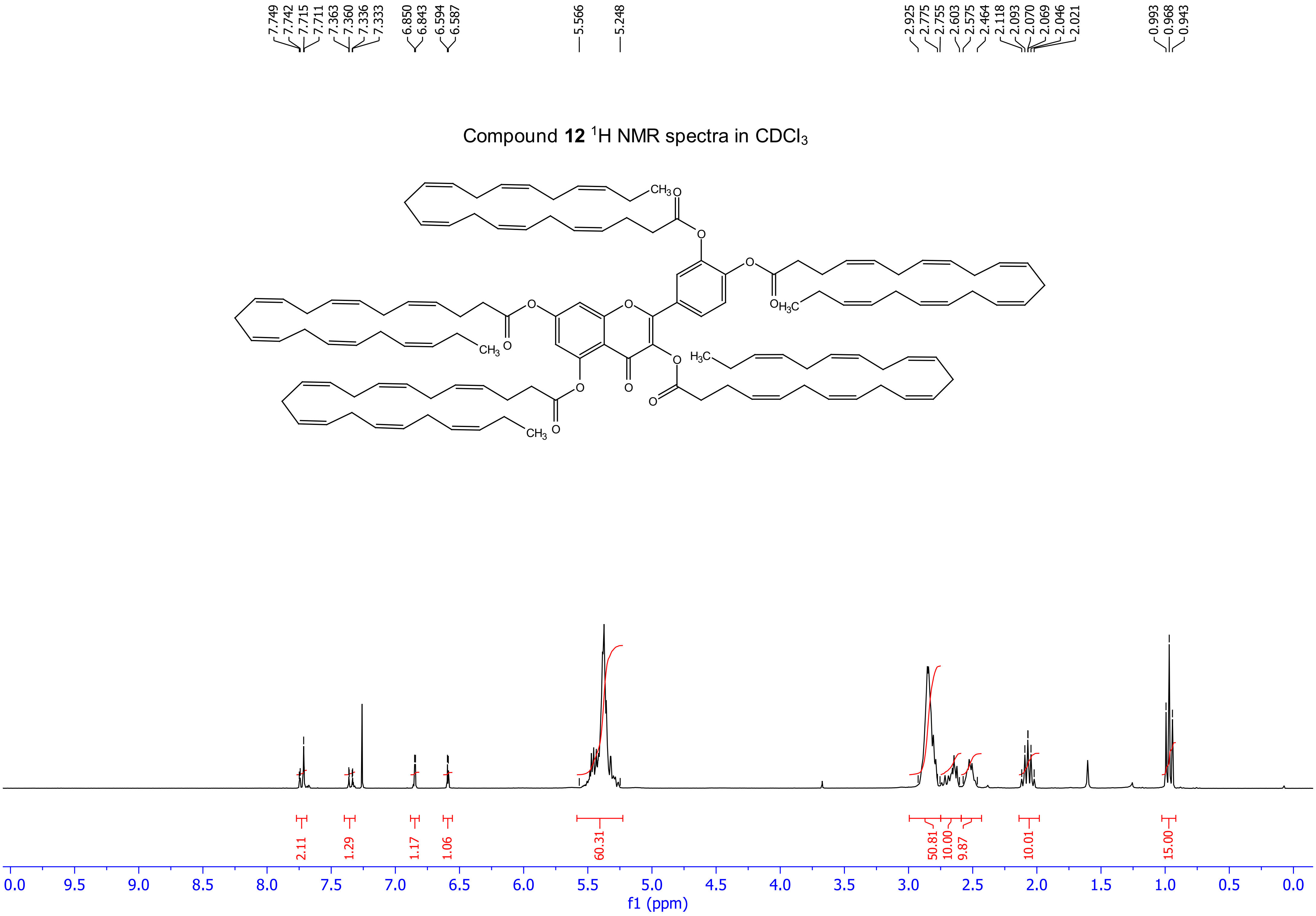

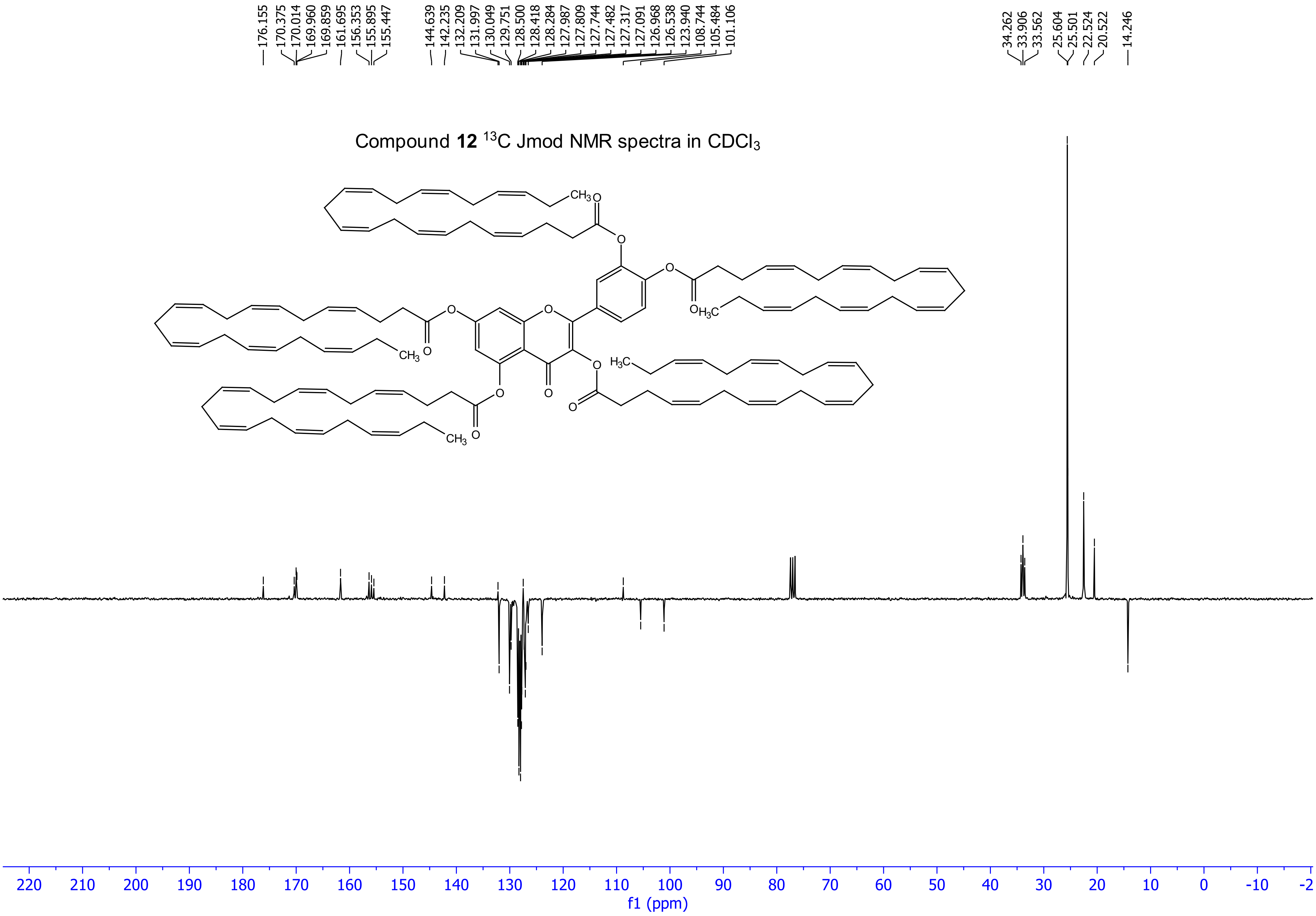
176.155
170.375
170.014
169.960
169.859
161.695
156.353
155.895
155.447
144.639
142.235
132.209
131.997
130.049
129.751
128.500
128.418
128.284
127.987
127.809
127.744
127.482
127.317
127.091
126.968
126.538
123.940
108.744
105.484
101.106
34.262
33.906
33.562
25.604
25.501
22.524
20.522
14.246
Compound **12** ^{13}C Jmod NMR spectra in $CDCl_3$
CH_3
O
H_3C
CH_3
f1 (ppm)
220 210 200 190 180 170 160 150 140 130 120 110 100 90 80 70 60 50 40 30 20 10 0 -10 -2

Quer-3-LA (compound **13**) ^{1}H NMR spectra in CD_3OD

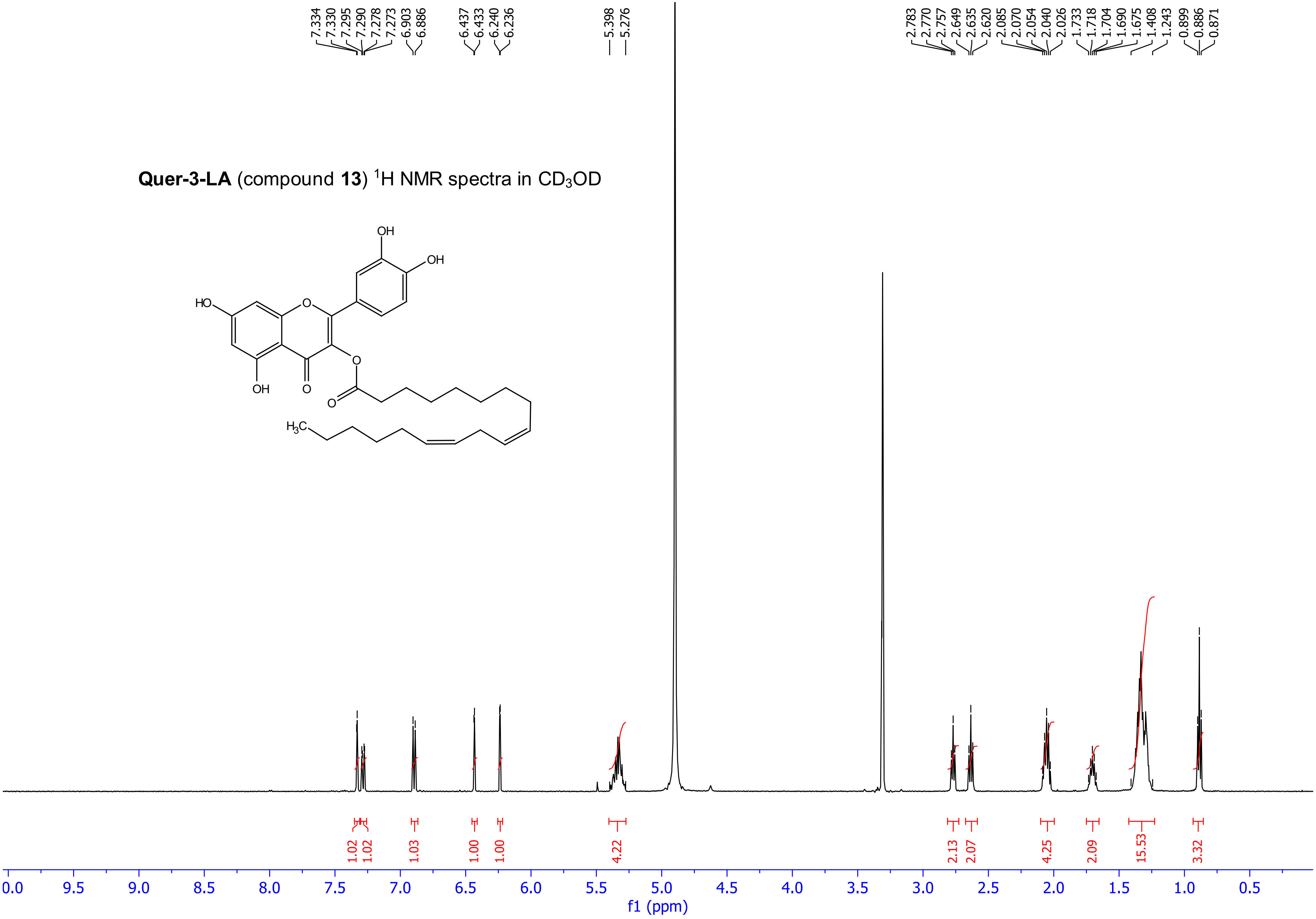

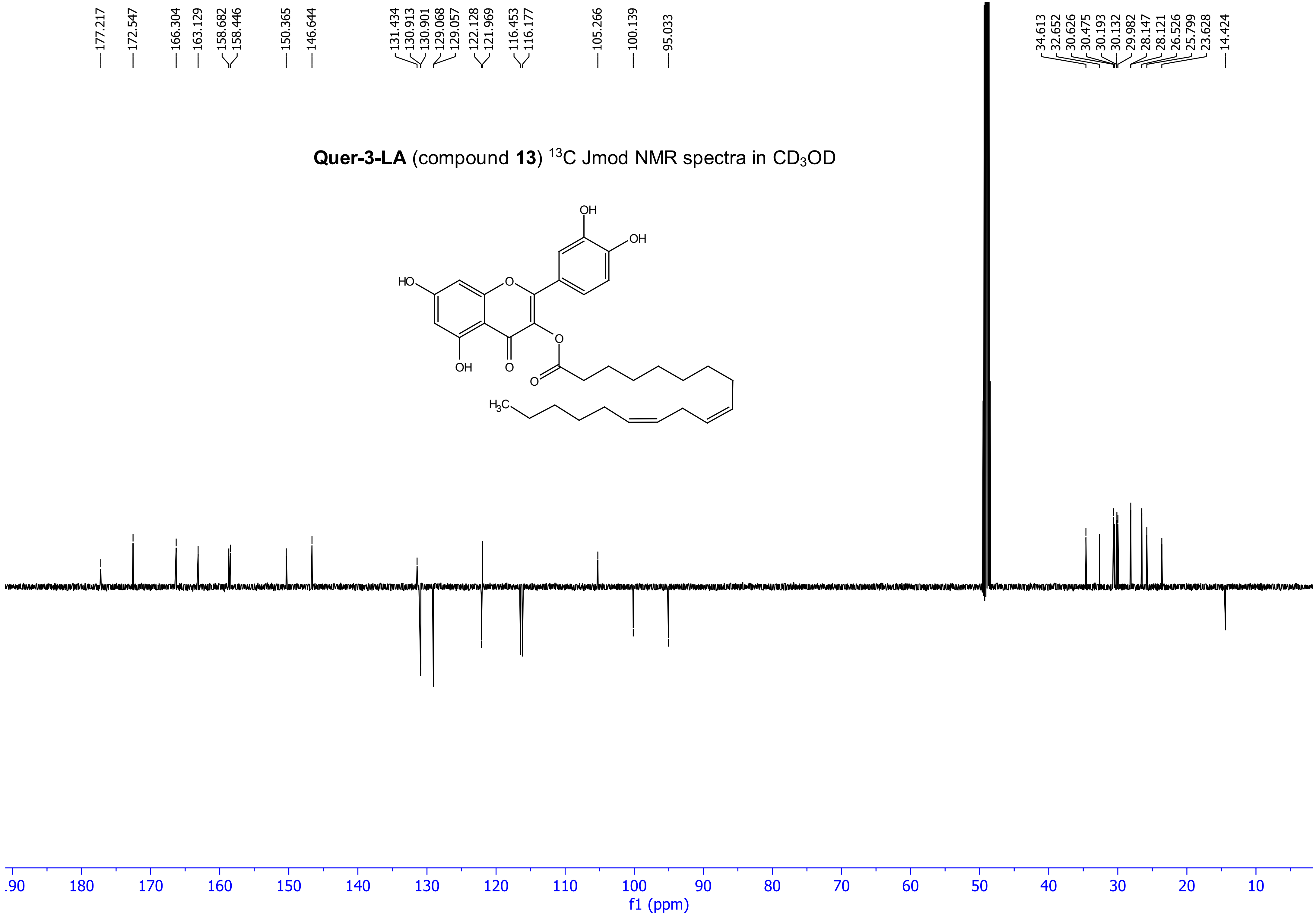
177.217
172.547
166.304
163.129
158.682
158.446
150.365
146.644
131.434
130.913
130.901
129.068
129.057
122.128
121.969
116.453
116.177
105.266
100.139
95.033
34.613
32.652
30.626
30.475
30.193
30.132
29.982
28.147
28.121
26.526
25.799
23.628
14.424
Quer-3-LA (compound 13) 13C Jmod NMR spectra in CD3OD
OH
OH
HO
O
O
OH
O
O
O
H3C
.90
180
170
160
150
140
130
120
110
100
90
80
70
60
50
40
30
20
10
f1 (ppm)

Quer-3-ALA (compound **14**) ^{1}H NMR spectra in CD_3OD

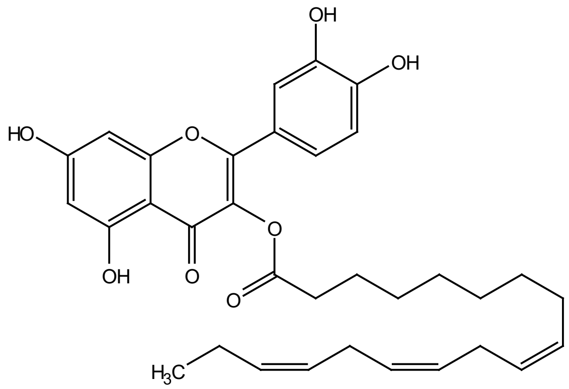

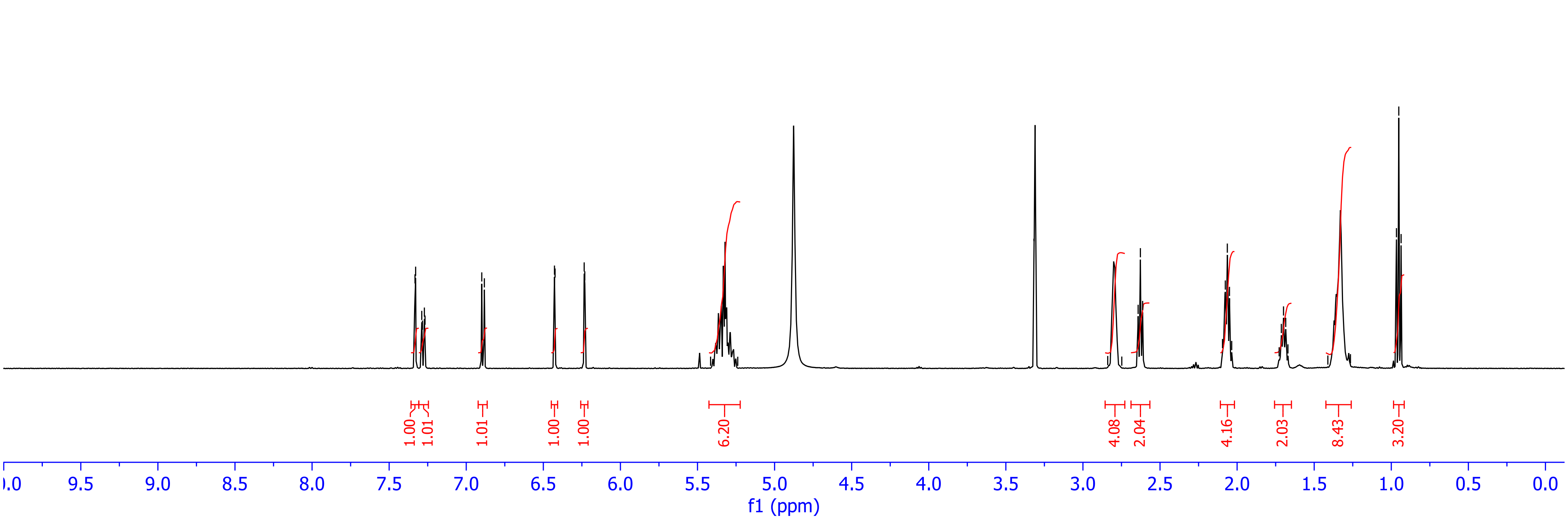

Quer-3-ALA (compound **14**) ^{13}C Jmod NMR spectra in CD_3OD

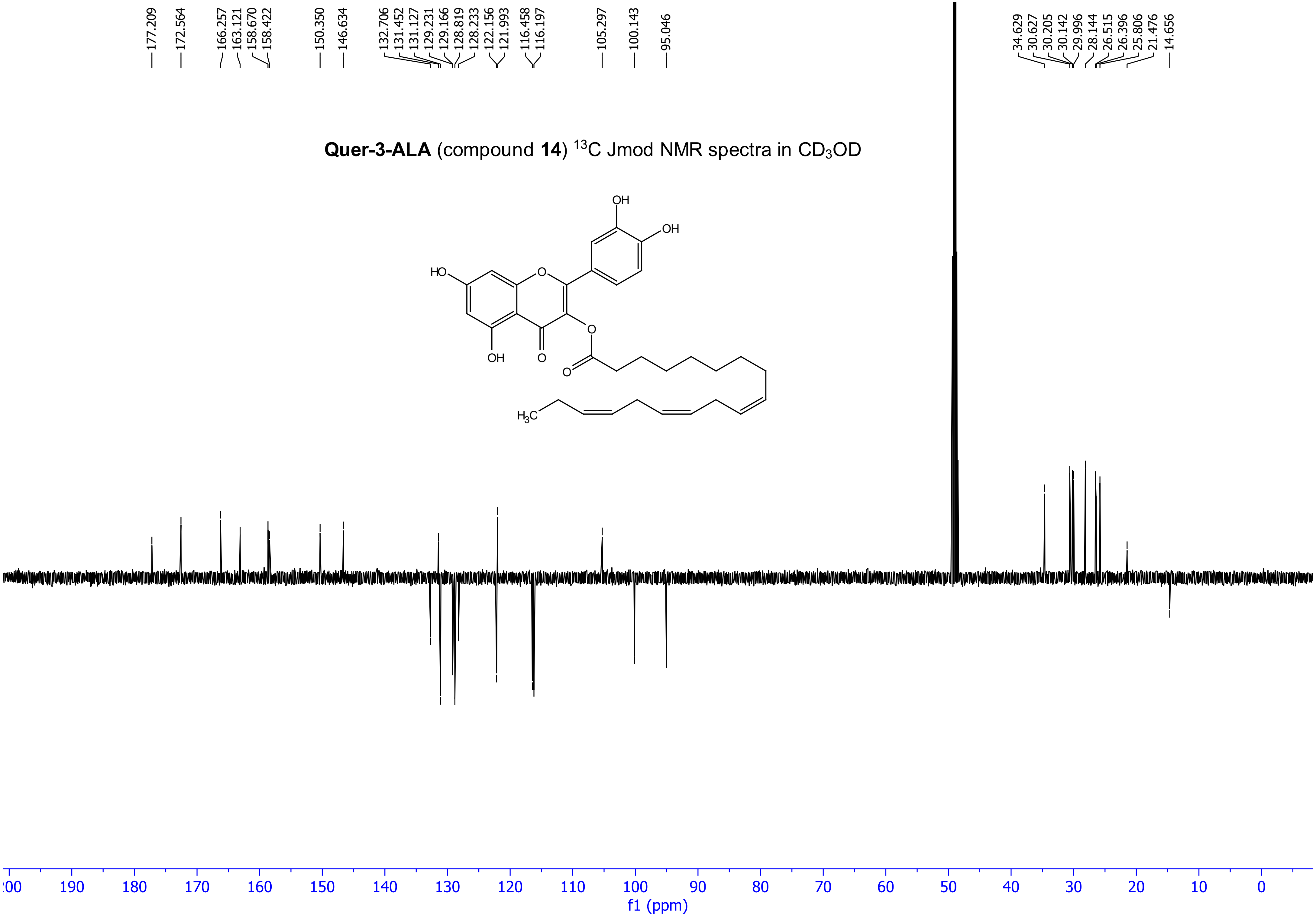

Quer-3-DHA (compound **15**) ^{1}H NMR spectra in CD_3OD

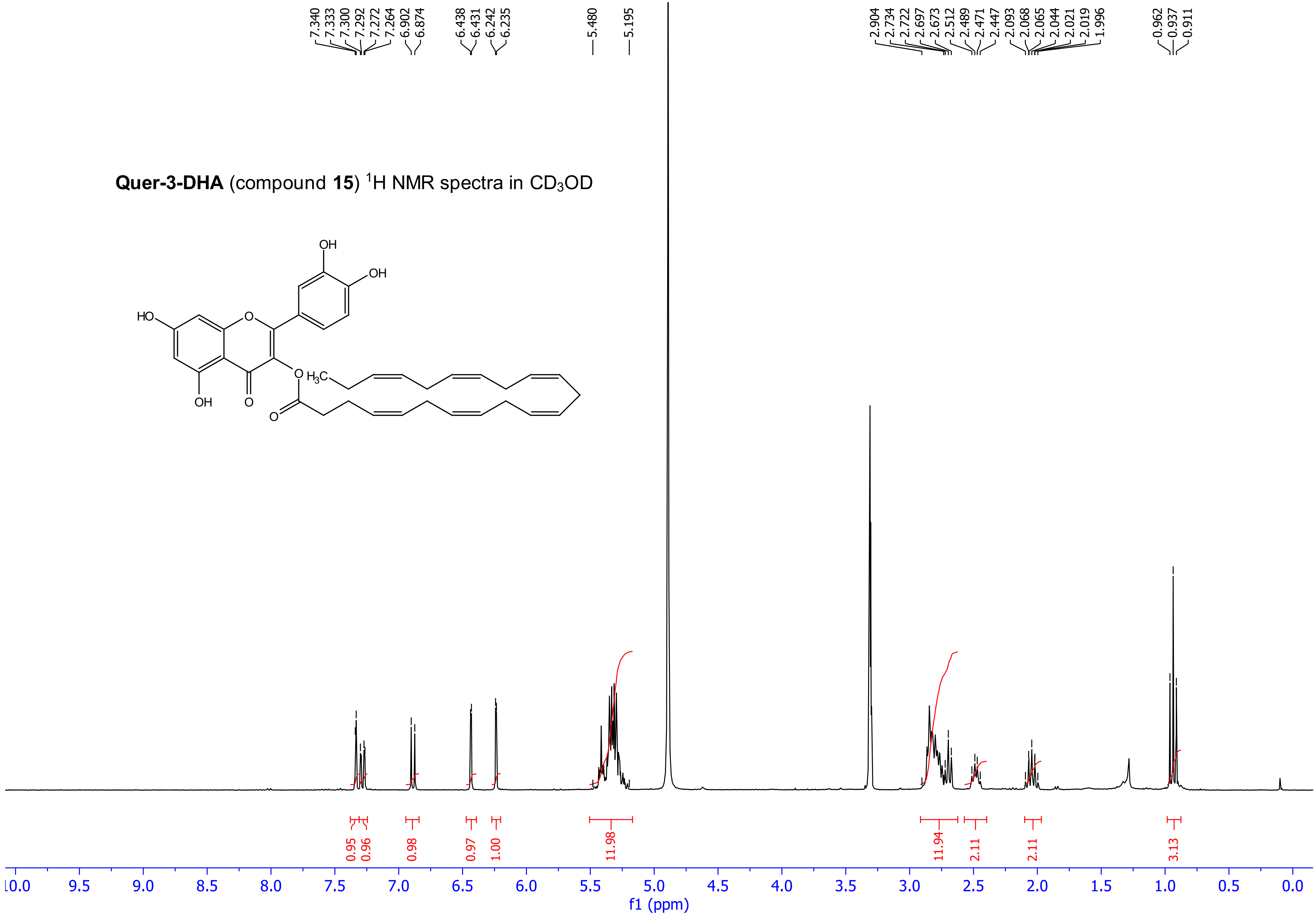

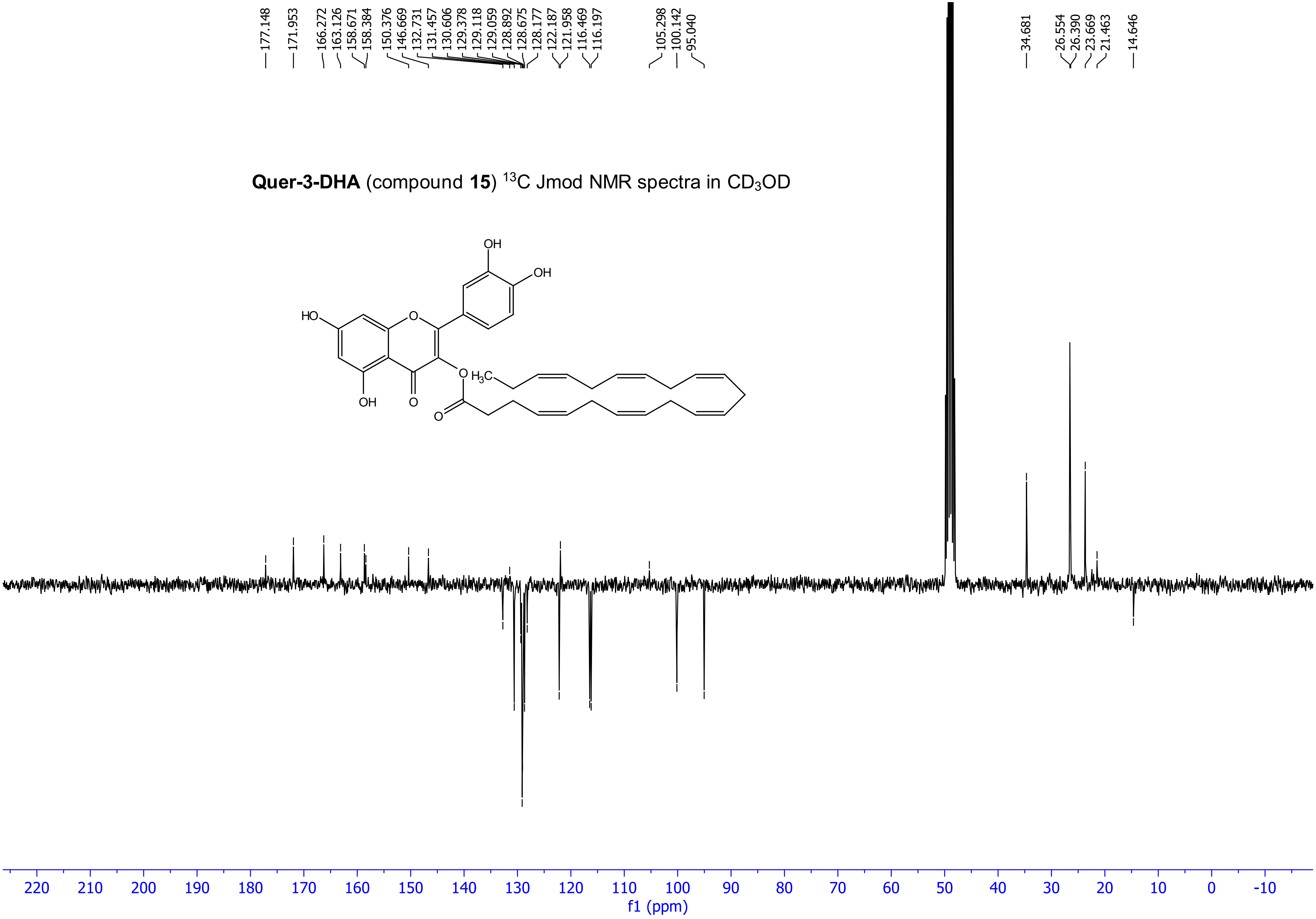

Quer-3-DHA (compound **15**) ^{13}C Jmod NMR spectra in CD_3OD

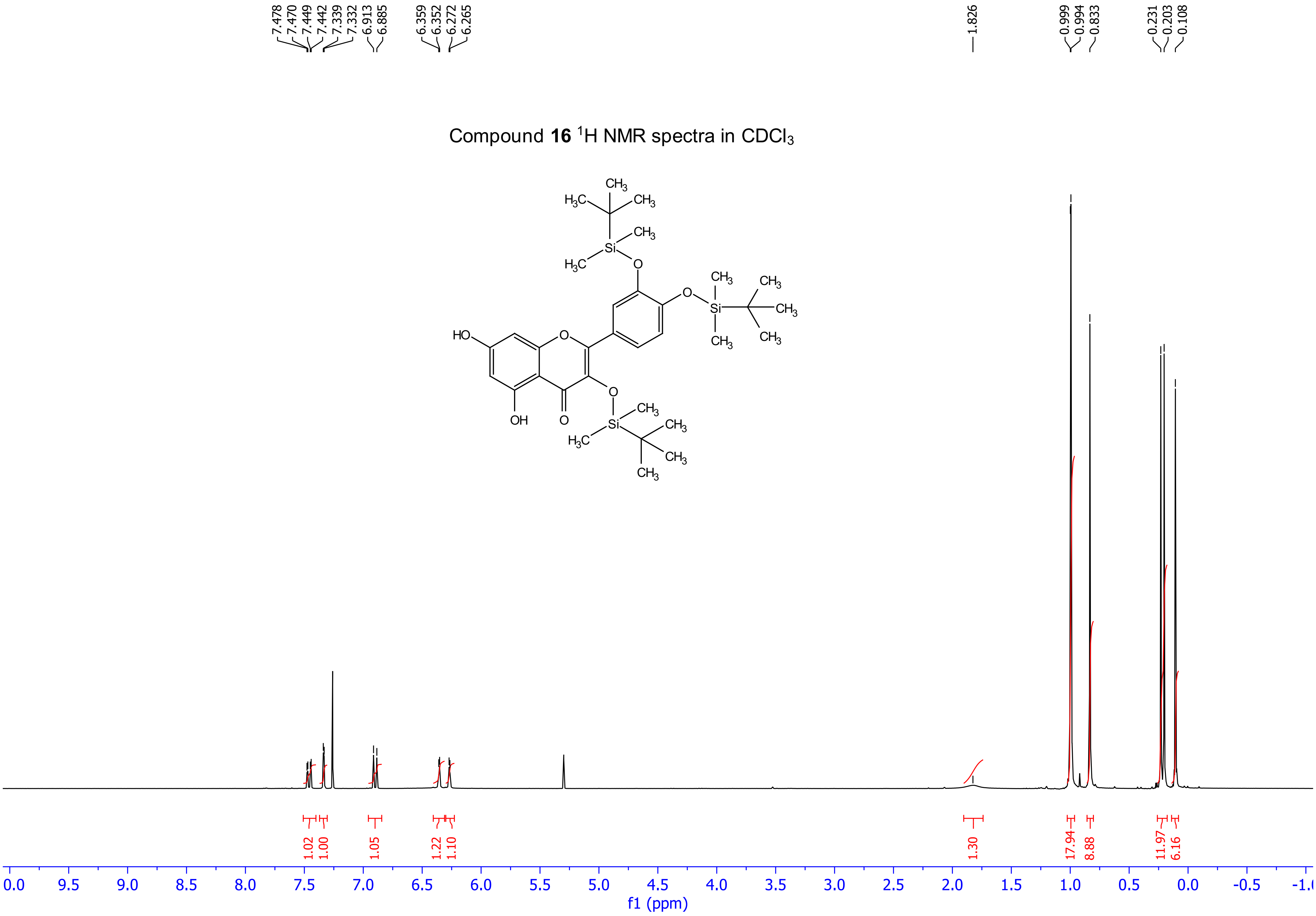
Compound **16** ^{1}H NMR spectra in $CDCl_3$
7.478
7.470
7.449
7.442
7.339
7.332
6.913
6.885
6.359
6.352
6.272
6.265
1.826
0.999
0.994
0.833
0.231
0.203
0.108
1.02
1.00
1.05
1.22
1.10
1.30
17.94
8.88
11.97
6.16
HO
OH
O
O
Si
CH3
H3C
0.0
9.5
9.0
8.5
8.0
7.5
7.0
6.5
6.0
5.5
5.0
4.5
4.0
3.5
3.0
2.5
2.0
1.5
1.0
0.5
0.0
-0.5
f1 (ppm)

Compound **16** ^{13}C Jmod NMR spectra in $CDCl_3$

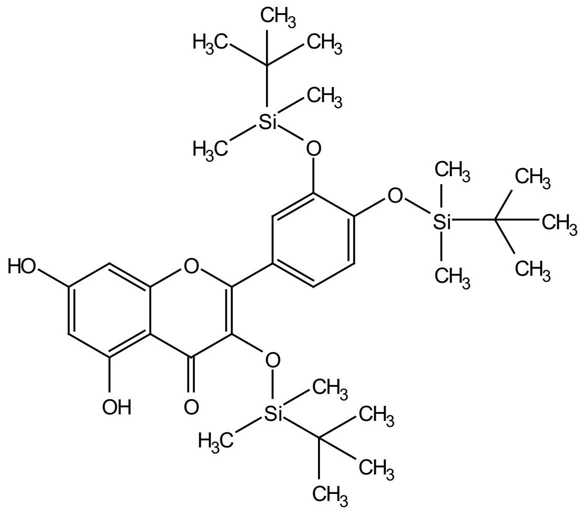

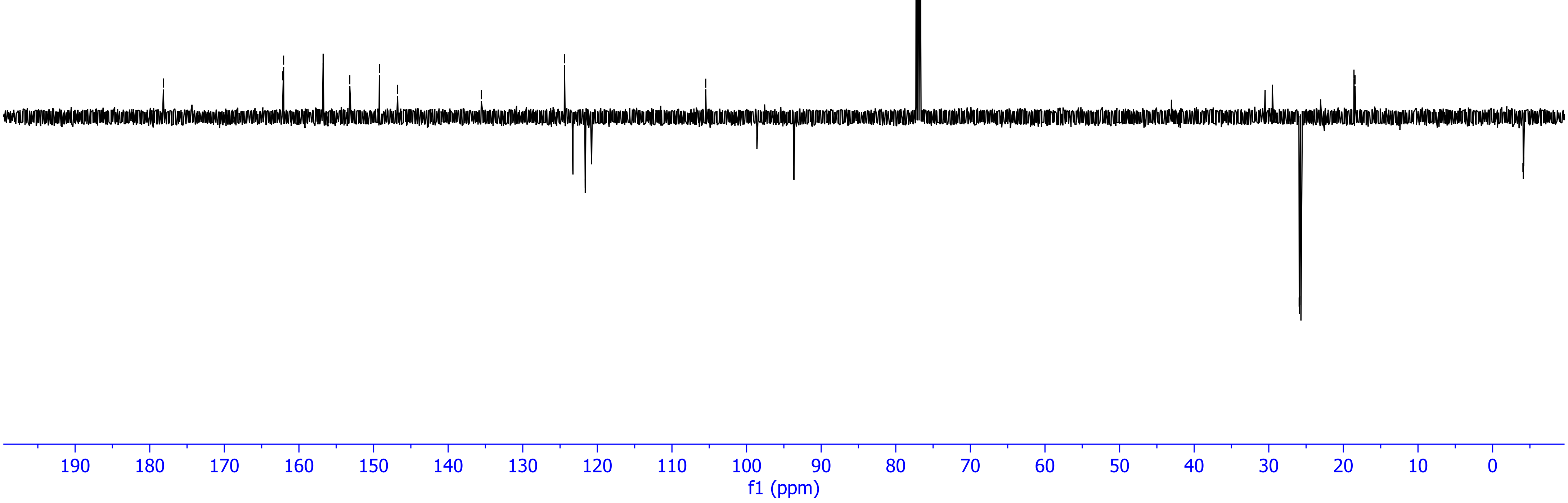

Compound **17** ^{1}H NMR spectra in $CDCl_3$

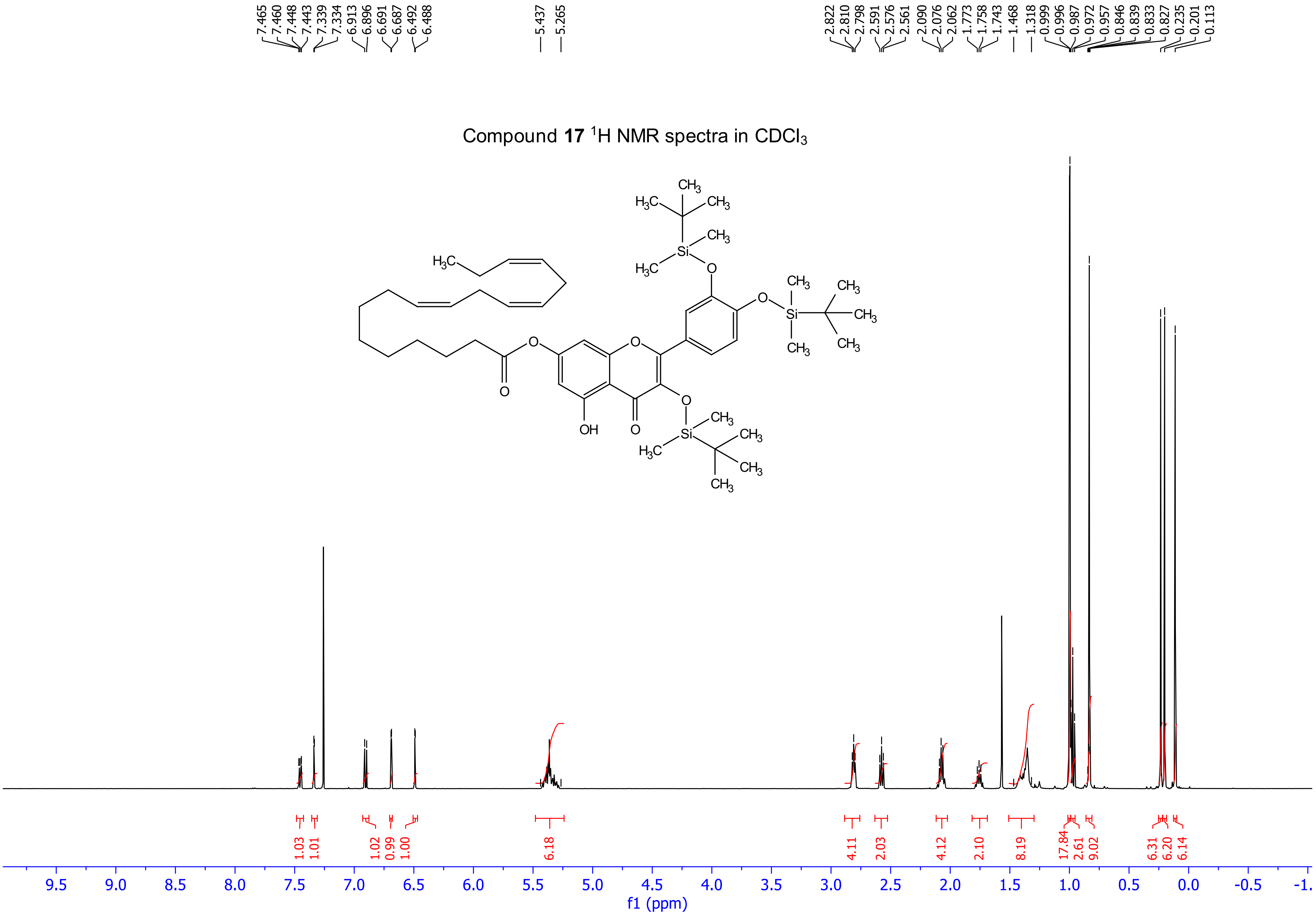

Compound **17** ^{13}C Jmod NMR spectra in $CDCl_3$

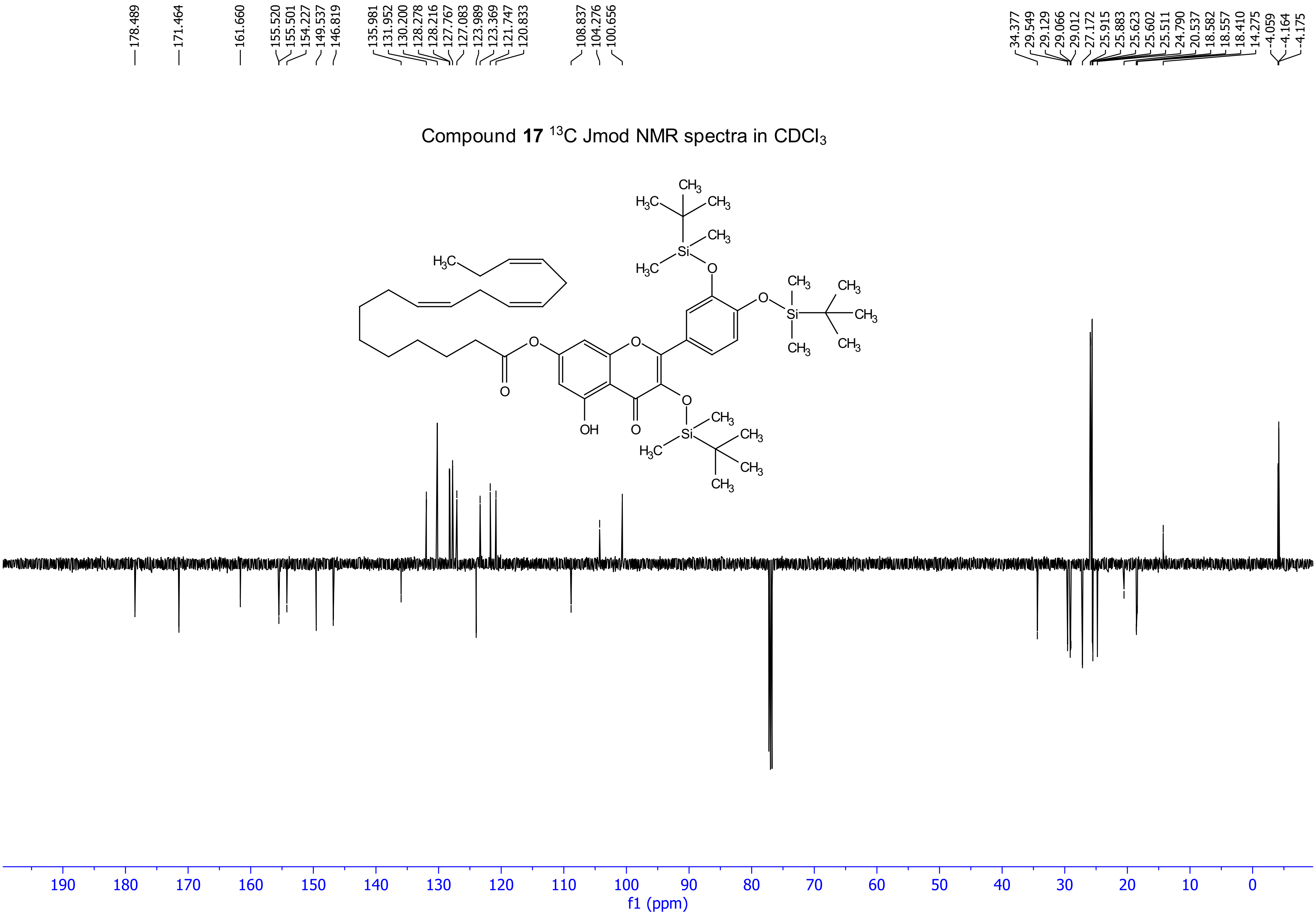

Quer-7-ALA (compound **18**) ^{1}H NMR spectra in CD_3OD

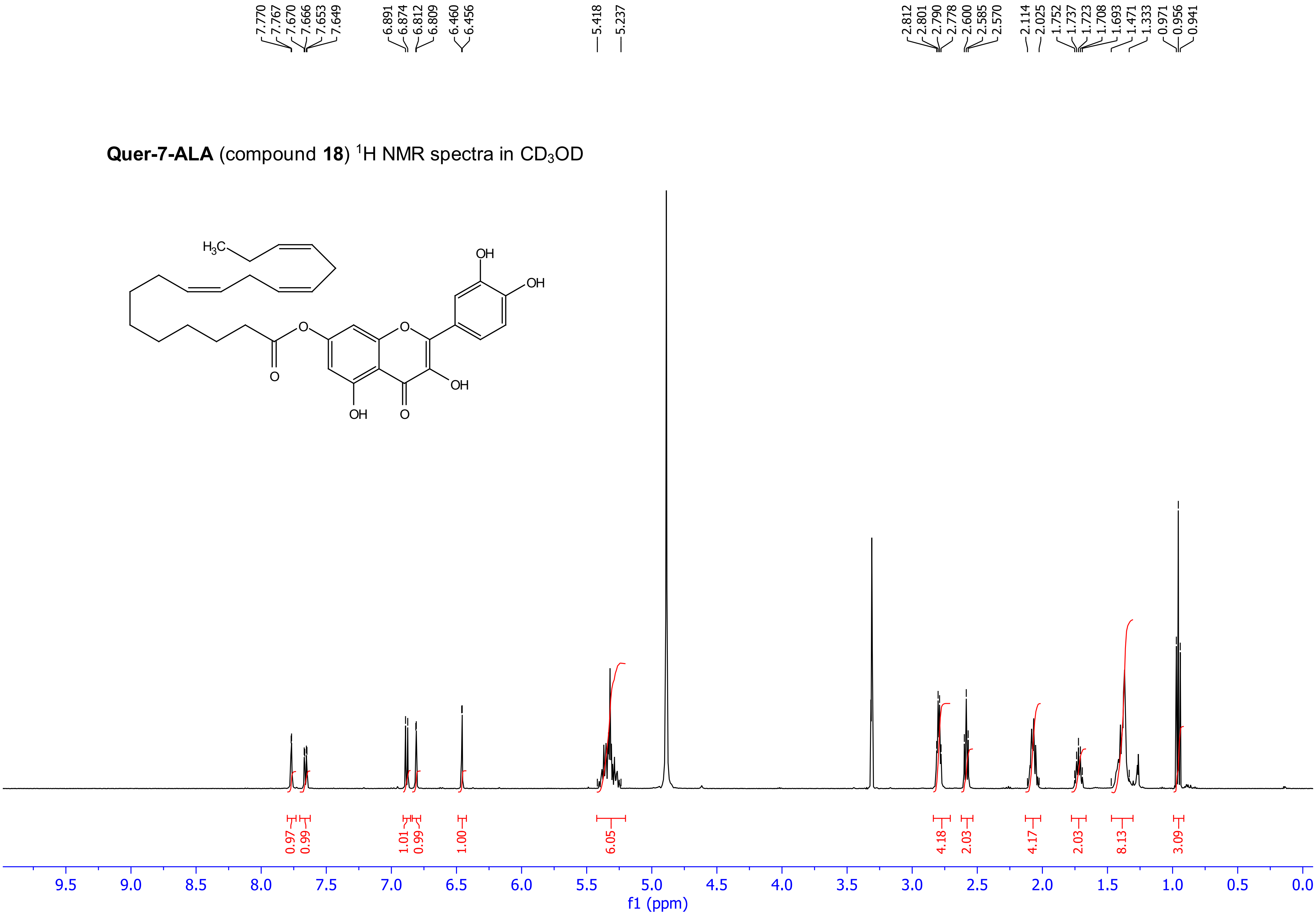

Quer-7-ALA (compound **18**) ^{13}C Jmod NMR spectra in CD_3OD

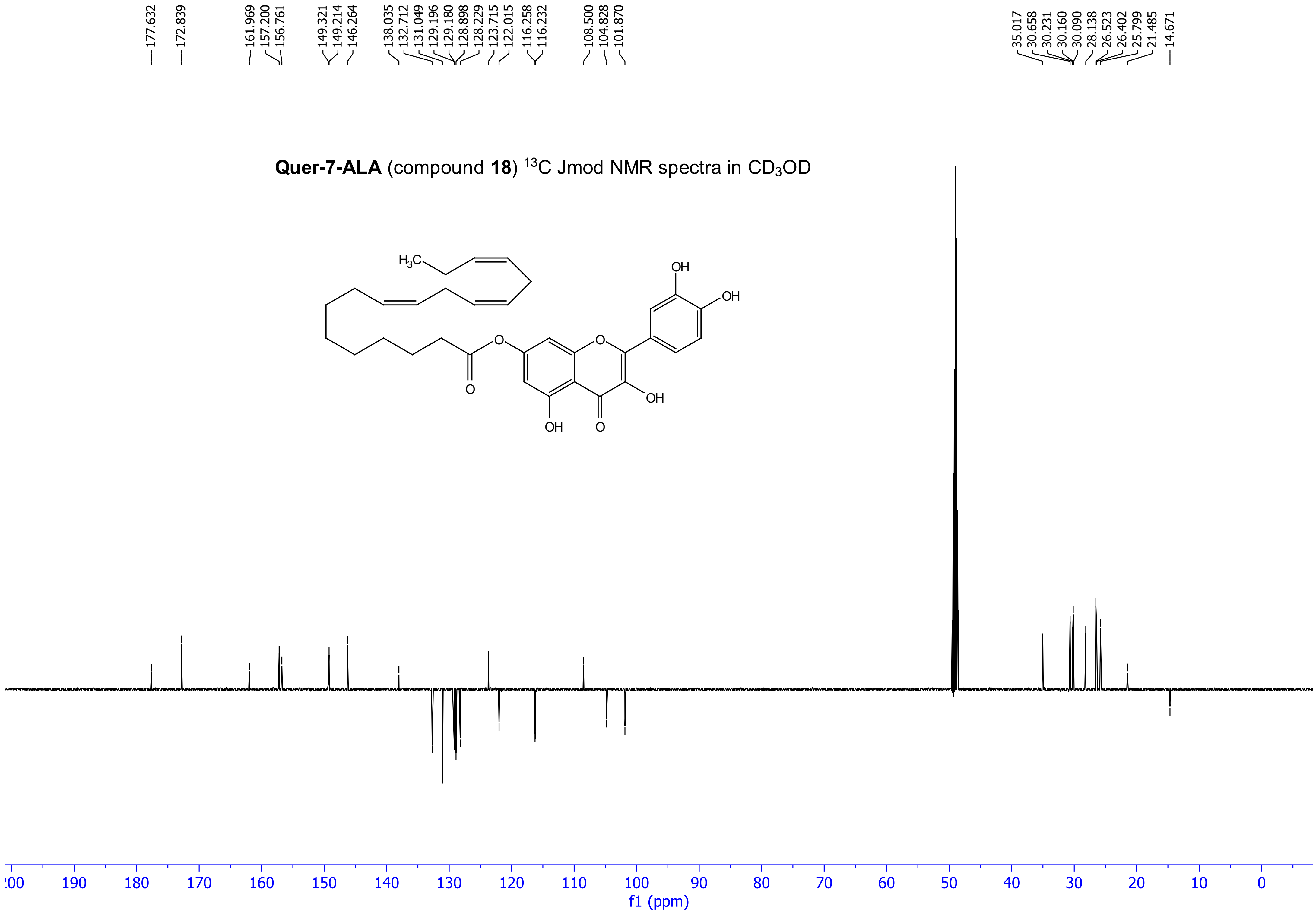

Supplement: Supplementary file 1 [file antioxidants-07-00197-s001.pdf]
